# Levofloxacin induces differential effects in the transcriptome between the gut, peripheral and axial joints in the Spondyloarthritis DBA/1 mice: Improvement of intestinal dysbiosis and the overall inflammatory process

**DOI:** 10.1371/journal.pone.0281265

**Published:** 2023-02-02

**Authors:** Susana Aideé González-Chávez, Joan S. Salas-Leiva, Dayana E. Salas-Leiva, Salma Marcela López-Loeza, Jasanai Sausameda-García, Erasmo Orrantia-Borunda, Rubén Burgos-Vargas, Maria Fernanda Alvarado-Jáquez, Mayra Torres-Quintana, Rubén Cuevas-Martínez, Eduardo Chaparro-Barrera, Carlos Marín-Terrazas, Gerardo Pável Espino-Solís, José Pablo Romero-López, Brian de Jesús Bernal-Alferes, César Pacheco-Tena

**Affiliations:** 1 Facultad de Medicina y Ciencias Biomédicas, Laboratorio PABIOM, Universidad Autónoma de Chihuahua, Chihuahua, México; 2 Departamento de Medio Ambiente y Energía, CONACyT-Centro de Investigación en Materiales Avanzados, Chihuahua, México; 3 Department of Biochemistry, University of Cambridge, Cambridge, United Kingdom; 4 Department of Biochemistry and Molecular Biology, Institute for Comparative Genomics (ICG), Dalhousie University, Halifax, NS, Canada; 5 Department of Rheumatology, Hospital General de México, "Dr. Eduardo Liceaga", Ciudad de México, México; 6 Translational Research Laboratory and National Laboratory of Flow Cytometry, Autonomous University of Chihuahua, Circuito Universitario, Campus II, Chihuahua, Mexico; 7 Laboratorio de Inmunología Clínica 1, Instituto Politécnico Nacional de México, Posgrado en Ciencias Quimicobiológicas, Escuela Nacional de Ciencias Biológicas, Ciudad de México, México; Universite Paris-Saclay, FRANCE

## Abstract

To analyze the effect of levofloxacin-induced intestinal microbiota modifications on intestinal, joint, and systemic inflammation in the DBA/1 mice with spontaneous arthritis. The study included two groups of mice, one of which received levofloxacin. The composition and structure of the microbiota were determined in the mice’s stool using 16S rRNA sequencing; the differential taxa and metabolic pathway between mice treated with levofloxacin and control mice were also defied. The effect of levofloxacin was evaluated in the intestines, hind paws, and spines of mice through DNA microarray transcriptome and histopathological analyses; systemic inflammation was measured by flow cytometry. Levofloxacin decreased the pro-inflammatory bacteria, including Prevotellaceae, Odoribacter, and Blautia, and increased the anti-inflammatory Muribaculaceae in mice’s stool. Histological analysis confirmed the intestinal inflammation in control mice, while in levofloxacin-treated mice, inflammation was reduced; in the hind paws and spines, levofloxacin also decreased the inflammation. Microarray showed the downregulation of genes and signaling pathways relevant in spondyloarthritis, including several cytokines and chemokines. Levofloxacin-treated mice showed differential transcriptomic profiles between peripheral and axial joints and intestines. Levofloxacin decreased the expression of TNF-α, IL-23a, and JAK3 in the three tissues, but IL-17 behaved differently in the intestine and the joints. Serum TNF-α was also reduced in levofloxacin-treated mice. Our results suggest that the microbiota modification aimed at reducing pro-inflammatory and increasing anti-inflammatory bacteria could potentially be a coadjuvant in treating inflammatory arthropathies.

## Introduction

Spondyloarthritis (SpA) is a heterogeneous group of rheumatic diseases characterized by enthesitis, arthritis, and axial skeleton involvement. They are linked to genetic susceptibility, primarily to HLA-B27 [1]. Several environmental factors can trigger the onset or relapse of these diseases; infection by arthritogenic bacteria is among the most important [2]. The relationship between immune response and microbiome is relevant to better understanding the pathogenesis of several diseases and identifying potential therapeutic targets [3,4].

SpA pathogenesis is associated with intestinal inflammation and arthritis/enthesitis, and is linked to the severity of both processes [5]. Since intestinal inflammation (mostly ileitis) is associated with changes in the microbiome and microbiota [6,7], it is plausible to establish a connection between these changes and intestinal inflammation and the severity and course of SpA. Despite these parallels, there is some independence in these processes; intestinal inflammation is not necessarily associated with musculoskeletal abnormalities (i.e., non-arthritic inflammatory bowel disease). Furthermore, axial component involvement does not necessarily correlate with the presence, severity, or progression of peripheral arthritis/enthesitis. Additional evidence for the heterogeneity of the inflammatory profile is the limited efficacy of some therapies on specific disease domains. For example, interleukin (IL)-17 antagonists show no benefit for intestinal inflammation [8], and IL-12/23 antagonists are not effective in the axial domain in axial SpA [9,10].

Several rodent models resemble human SpA [11]. Housing rodents in germ-free conditions precludes the onset of arthritis; however, when the rodents grow in a non-sterile environment, arthritis develops. Instead of active infections by pathogenic bacteria, arthritis has been linked to specific profiles in the commensal microbiota [12–14]. Specific bacteria allegedly play either a favorable or detrimental role in arthritis; however, in animal models of arthritis, the results vary significantly between different animal models of arthritis [15–17]. Moreover, inconsistencies are found even within the same model. Despite the heterogeneity in the results, it is widely accepted that bacteria can establish a specific relationship with host barriers, which may influence the presence or absence of inflammation [18–21].

Young male mice of the DBA/1 strain develop a spontaneous arthritic process if caged in a confined tight space, which resembles human SpA. They develop peripheral arthritis and enthesitis with secondary bone overgrowth, which also affects the axial skeleton [22–24]. Furthermore, because the confined young males fight aggressively, bacterial dissemination from the resultant skin wounds could possibly induce or perpetuate arthritis/enthesitis. To our knowledge, intestinal inflammation has not been described in DBA/1 mice [12]. Given the pathogenic connection between intestinal inflammation, dysbiosis, and joint inflammation in patients with SpA, a detailed exploration of this link in the DBA/1 mice is essential to assess the influence of bacterial communities in the gut and joints.

The role of microbiota in physiological and pathological processes has been widely assessed through bacterial depletion using antibiotics [15,16,25]. Since bacterial dissemination from wounds was a potential pathogenic mechanism in DBA/1 mice with spontaneous arthritis, a systemic, rather than an intraluminal, antibiotic was chosen. Levofloxacin is a quinolone with a broad bactericidal spectrum, including gram-positive and gram-negative bacteria. It is used to treat respiratory, urinary, and gastrointestinal infections, and is assumed to induce a significant modification of the gastrointestinal microbiota.

As the underlying mechanisms linking alterations in the gut microbiome to joint inflammation in SpA have not been fully understood, we aimed to analyze the effects of levofloxacin-induced intestinal microbiota modifications on intestinal, joint, and systemic inflammation in the DBA/1 mice with spontaneous arthritis through DNA microarray transcriptome analyses, histopathology, and flow cytometry.

## Methods

### Animals

The study included 12 male DBA/1 mice aged nine weeks, randomly divided into two groups of six mice each, one received levofloxacin (experimental group), and the other did not (control group). Spontaneous arthritis was induced as described by Braem et al., [22] using standard breeding cages adapted to promote the dimensions of 56 cm2 per mouse. The experimental group received levofloxacin in drinking water at a 1 mg/ml concentration for 20 weeks. Mice from both groups were kept in the same building under controlled luminosity (12 h light / 12 h dark) and temperature (23 ± 2°C); they were handled by the same people and received the same type of bedding, food, and water (ad libitum). This study complied with the Official Mexican Standard NOM-062-ZOO-1999, technical specifications for producing, caring for, and using laboratory animals. The research was approved by the Ethics Committee and Institutional Animal Care and Use Committee (IACUC) from the Faculty of Medicine and Biomedical Sciences of the Autonomous University of Chihuahua (ID number: CI-036-20).

### Microbiota identification in mice’s stool

Before sacrifice, stool samples from mice of both groups (six mice per group) were individually collected in sterile containers using RNAlater™ (Qiagen, Hilden, Germany) for nucleic acid preservation. The samples were stored at -80°C until DNA extraction.

Following Illumina’s standard amplicon protocols, sample preparation and DNA sequencing were performed at the Microbial Genomics Laboratory (CIAD, Mexico). The V3 region of the 16S rRNA gene was amplified with universal primers 338F (5′-ACTCCTACGGGAGGCAGCAG-3′) and 533R (5′-TTACCGCGGCTGCTGGCAC-3′). The products were prepared according to the protocol recommended by Illumina. First, samples were quantified on Qubit, mixed in an equimolar group, and sequenced on the Illumina Miniseq platform under standard conditions (300 cycles, 2x150 pair-end). Next, low-quality adapters and bases were removed from the sequences and processed with the Quantitative Insights Into Microbial Ecology (QIIME2) software pipeline [26] via Microbiome Helper [27], following the Divisive Amplicon Denoising Algorithm (DADA2) pipeline designed for amplicon standard operating procedure screening, assembling, and elimination of chimeric 16S rRNA sequences. The resulting sequences were grouped into operational taxonomic units (OTU) using the 97% identity criterion with Sklearn and SILVA database v138 (https://www.arb-silva.de/). Then, low confidence OTUs (0.1%) were removed, and the resulting file “feature-table_w_tax.biom” was used to estimate diversity with Simpson and Shannon indices and Chao-1 richness estimator for each sample employing MicrobiomeAnalyst software [28]. For this purpose, the data were filtered and normalized according to the recommendations of Chong et al. [28] (i.e., data filtered by a minimum count of 4, the prevalence in samples of at least 20%, and with a low variance of 10% based on interquartile range (IQR)). Subsequently, the data were normalized by testing two methods separately: total sum scale (TSS) and relative log expression (RLE).

The relative abundances (Arel) of the taxa were used to depict the composition and structure of the community at the level of phylum and class using bar diagrams. A principal coordinate analysis (PCoA) was performed by applying the Bray-Curtis index dissimilarity distance, and the groups were compared with Permutational multivariate analysis of variance (PERMANOVA) and analysis of similarities (ANOSIM) (p<0.05) to determine beta diversity. To assess which taxa were differently abundant between the control and levofloxacin-treated group, three methods implemented in the MicrobiomeAnalyst platform were applied: i) Mann-Whitney/Kruskal-Wallis, ii) Zero-inflated Gaussian fit, and iii) EdgeR. False Discovery Rate (FDR) was considered at p<0.05. Subsequently, a Venn diagram (https://bioinfogp.cnb.csic.es/tools/venny/index.html) was obtained to identify the taxonomic groups that were common to all three methods (p<0.05). These analyses were done at the genus level. Still, in those cases where there was no taxonomic classification for that level, we report the last rank for which the classification was known.

Finally, a predictive functional analysis of the metabolic pathways based on the diversity of OTUs was carried out with the pipeline of Phylogenetic Investigation of Communities by Reconstruction of Unobserved States (PICRUSt2 v. 2.3.0b) with default configuration [29]. With the Nephele implementation of PICRUSt2, we inferred the abundance of metabolic pathways based on the MetaCyC database (https://biocyc.org) using the enzyme commission numbers (EC) (https://nephele.niaid.nih.gov/show_picrust2_details/). Once the metabolic pathways were identified, a linear discriminant analysis was performed together with the effect size (LEfSe) (LDA two2 and p<0.05) (https://huttenhower.sph.harvard.edu/galaxy/) [30] to identify the most relevant routes metabolic in each evaluated group.

### Histological analysis

After mice’s euthanasia with isoflurane (Laboratorios PISA, S.A. de C.V, Mexico), the ileocolic junction, hind paws, and spines were dissected and fixed in 10% phosphate-buffered formalin for 48 hours, then the hind paws and spines were demineralized using 5% nitric acid for 24 hours. All tissues were subsequently dehydrated in graded ethanol and embedded in paraffin. Sections of 5μm in thickness were obtained and placed on adhesive-coated glass slides [31].

Histological evaluation was performed using hematoxylin & eosin (H&E) stains. The images were acquired using a digital camera (AmScope MU1803) coupled with an optical microscope (AxioStar Plus, Carl Zeiss). The arthritis severity was evaluated in hind paws using the semiquantitative scale as 0: absent; 1: mild; 2: moderate; or 3: severe to describe inflammatory infiltrate, synovial hyperplasia, cartilage damage, enthesitis, cartilage neoformation, bone neoformation, and ankylosis. The mean score was calculated for each group.

The mice’s gut inflammation was evaluated as described by Erben et al. [32]. In the H&E staining of ileocolic junctions, inflammatory severity was scored as 1: minimal <10%, 2: mild 10–25%, 3: moderate 26–50%, or 4: marked ≥ 51%; infiltration extent as grade 1: mucosal, 2: mucosal and submucosal, or 3: transmural. Epithelial changes were assessed by scoring the hyperplasia, goblet cell loss, and erosion as 1: minimal <10%, 2: mild 10–25%, 3: moderate 26–50%, or 4: marked ≥ 51%. Cryptitis, crypt abscesses, and ulceration were scored as 0: absent or 3: present. The mucosal architecture was evaluated according to the presence of granulation tissue, irregular crypts, and crypt loss as 0: absent or 4: present. Villous blunting was classified as 1: mild (villous to crypt length ratio of 2:1 a 3:1), 2: moderate (villous to crypt length ratio of 1:1), or 3: villous atrophy.

### DNA microarray and bioinformatic analysis

RNA was purified from mice’s ileocolic junction, hind paws, and spines using the RNeasy® Mini Kit (Qiagen, Hilden, Germany) extraction kit, following the manufacturer’s protocol. RNA quantity and quality were verified in the Qubit4 fluorometer (Thermo Fisher). RNA from each mouse’s ileocolic junction, hind paws, and spine was mixed in equimolar quantities to conform pools used in the DNA microarrays.

The DNA microarrays were performed at the Institute of Cellular Physiology, Autonomous University of Mexico (UNAM), Mexico. Briefly, the complementary DNAs (cDNAs) were synthesized by reverse transcription-polymerase chain reaction (RT-PCR) and labeled with Cy5 (control group) or Cy 3 (levofloxacin-treated groups). Hybridization was done using an M22K_01 (UNAM, Mexico) chip containing 22,000 genes from the mouse genome. The scan and acquisition of the signal were developed using the ScanArray 4000 (Packard BioChips Technologies, Billerica, Massachusetts, USA). The analysis of the microarray scanning was made using GenArise Microarray Analysis Tool software (UNAM, Mexico), and the lists of differentially expressed genes (DEGs) (Z-score ≥ 1.5 SD) in mice treated with levofloxacin respect control group were obtained.

The bioinformatics analysis was carried out in the STRING 11.0 database (https://string-db.org/) to integrate the direct and indirect protein-protein interactions (IPP) based on functional associations [33]. The DEGs (Z-score ≥ 1.5 SD) were loaded, and the interactions were selected with minimal confidence (interaction score > 0.4). The obtained IPP networks were analyzed to obtain primary clusters of subnetworks (highly interconnected regions) using the Cytoscape software v. 3.7.0 [34] with the Molecular Complex Detection (MCODE) complement [35,36] (degree cutoff = 2 and node score cutoff = 0.3). The genes of the first three clusters, both down-expressed and up-expressed, were again analyzed on STRING to obtain the associated KEGG (Kyoto Encyclopedia of Genes and Genomes) signaling pathways. In the IPP networks, those pathways related to inflammatory and metabolic processes of interest in arthritis were selected and marked with different colors to identify the genes with the highest number of interactions.

In the STRING database, the PPI enrichment p-value is calculated and indicates that the nodes are not random and that the observed number of edges is significant; moreover, in the analyses of the associated-KEGG pathways, the expected proportion of type I errors is defined as FDR = E(V/R | R > 0) P(R > 0) [37]. In Cytoscape-MCODE, the complex score is defined as the product of the complex subgraph, C = (V, E), density, and the number of vertices in the complex subgraph (DC × |V|) [36].

### Immunohistochemistry

Immunohistochemical (IHC) was performed with specific polyclonal antibodies against TNF-α (sc-52746), IL-23a (sc-271349), IL-17a (sc-374218), and Jak3 (sc-513) (Santa Cruz Biotechnology, CA, USA). Tissue sections were deparaffinized in two changes of xylene and dehydrated in descending concentrations of ethanol until water. Antigen retrieval was performed with 0.001 M EDTA at 80°C; the slides were then treated with 0.2% Triton-X100 (Bio-Rad, Hercules, CA, USA). After blocking with 10% bovine serum albumin (BSA) (Sigma Life Science, MO, USA), the tissues were incubated with the primary antibody diluted 1:200 in 1% BSA at 4°C overnight. The corresponding isotype’s biotin-streptavidin-conjugated secondary antibodies (Jackson ImmunoResearch Laboratories, Inc, PA, USA) were used in a 1:400 dilution. Immunodetection was performed using Pierce® streptavidin horseradish peroxidase-conjugated (Jackson ImmunoResearch Laboratories, Inc, PA, USA) and Diaminobenzidine (DAB) as the chromogen. The primary antibody was replaced with phosphate-buffered saline (PBS) to establish the negative controls. Images were acquired using a digital camera (AmScope MU1803) coupled with an optical microscope (AxioStar Plus, Carl Zeiss), taking at least 20 microscopic fields from each slide. The expression of each marker was quantified with the ImageJ program and the IHC toolbox. The DAB color was extracted from each image, and the maximum and mean gray values were obtained. Each image’s optical density (OD) was obtained with log10 (maximum gray value / mean gray value). The OD means, and standard deviations were calculated and graphed for each study group.

### Quantification of serum cytokines by flow cytometry

Serum samples were obtained from mice previous to sacrifice for the measurement of TNF-α, IFNγ, IL-6, IL-10, and monocyte chemoattractant protein-1 (MCP-1) by flow cytometry using the Mouse Inflammation Kit: Cytometric Bead Array (Becton Dickinson, CA, USA). Briefly, precoated microbeads with capture antibodies were mixed and incubated with non-diluted murine serum and phycoerythrin-conjugated detection antibodies. Then, beads were resuspended in a 300 μl volume and tested using FL4 laser for clustering detection and FL2 laser for reporter detection using the Accuri C6 Plus flow cytometer (Becton Dickinson, CA, USA). The data obtained were analyzed using FCAP Array Software.

### Statistical analysis

For histological analysis, IHC, and flow cytometry, statistical analysis was made in SPSS statistics v22 software (SPSS Science Inc., Chicago, IL, USA). Measures of central tendency and dispersion were estimated for each variable. The t-test was used to determine the effect of levofloxacin by comparing the treated and non-treated groups. Differences were considered significant when p≤0.05. The statistical analyses of the microbiome sequencing and microarrays are detailed in the corresponding sections.

## Results

### Levofloxacin decreases the pro-inflammatory prokaryotic community and deregulates its metabolic pathways in the stool of DBA/1 mice with spontaneous arthritis

High-throughput sequencing methods for microbial characterization in a culture-independent manner have made it increasingly clear that the microbiome plays an essential role in human health and disease. Therefore, our study focused on evaluating the effects of the levofloxacin-induced intestinal microbiota modifications on intestinal, joint, and systemic inflammation in a murine model of SpA. Initially, the stool microbiomes of levofloxacin-treated and untreated mice were sequenced, and bioinformatic analysis determined the structure and diversity of the prokaryotic community in each group and the differences between groups.

The microbiome of stool samples was analyzed by sequencing the V3 region of the 16S rRNA gene. A total of 12 libraries were sequenced; six corresponded to the control group and six to mice treated with levofloxacin. A total of 477,136 assembled sequences were obtained. The number of sequences in the control samples ranged from 32,487 to 45,078 with an average of 39,957 ± 4,482 (mean ± SD); the levofloxacin-treated samples ranged from 34,165 to 45,800 with an average of 39,566 ± 4,527. These sequences were assigned to 325 OTUs according to a 97% similarity rule. The microbiome sequences were registered in the Sequence Read Archive (SRA) database of the National Center for Biotechnology Information (NCBI), ID PRJNA887434).

**Fig 1:**
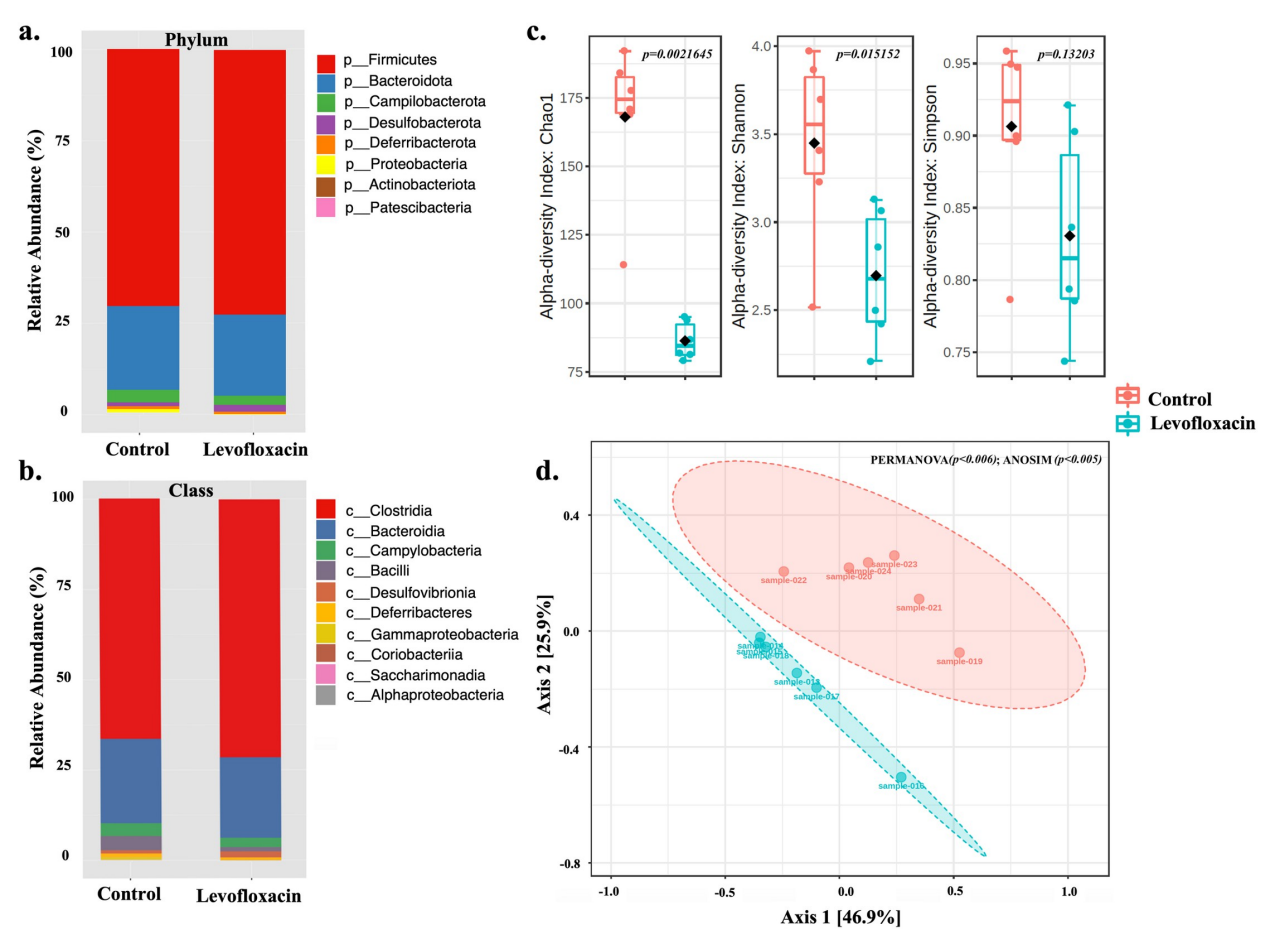
Composition and structure of the stool microbial community from control and levofloxacin-treated mice (n = 6 mice per group) at the a. Phylum and b. Class level; c. Richness (Chao-1) and alpha diversity (Shannon and Simpson) indices, Statistical test Mann-Whitney (p<0.05); d. Beta diversity at the OTU level is represented spatially by a principal coordinate diagram (PCoA) using Bray Curtis dissimilarity distances, Statistical test PERMANOVA and ANOSIM (p<0.05).

The microbiota consisted of eight phyla, with Firmicutes being the dominant phylum in the two evaluated groups (Fig 1A). This taxon constituted at least 65% of the Arel of the entire microbial community of the analyzed samples. The second phylum with the highest Arel was Bacteroidota, which constituted 18 and 20% of the taxa in the control and treated groups. In the control group, higher Arel for Proteobacteria and Campylobacterota was observed than in the levofloxacin-treated group (Fig 1A). Ten classes were detected in the two groups analyzed at the class level. The Clostridia and Bacteroidia groups had the highest Arel representing at least 87.5% of the community (Fig 1B). The control group observed greater Arel for the Bacilli, Gammaproteobacteria, and Campylobacteria classes than those found in the levofloxacin-treated group (Fig 1B). The abundance-based species richness estimator (Chao-1) was higher in the control group than in the levofloxacin-treated group (p<0.05) (Fig 1C). The treatment with levofloxacin exhibits a low richness near half of that observed in the control. Regarding diversity, on average, the Shannon index was lower in the stool microbiota of mice treated than in the control group (p<0.05). The Simpson index presented similar behavior to that of Shannon; however, these were not statistically different, suggesting that the two groups have similar dominance of species (p> 0.05) (Fig 1C). The PCoA analysis indicated that 72.8% of the variability of the taxonomic profiles of the studied samples could be explained by two axes (Axis1 46.9% and Axis2 25.9%) (Fig 1D). In this analysis, two well-defined groups were observed, one containing the microbial profiles of the control and another one with levofloxacin-treated samples (Fig 1D). ANOSIM and PERMANOVA statistical analyses indicated a significant difference between and within groups (p<0.05). Control samples showed a broader spatial distribution than the treated samples, in which a grouping of 83% of the samples is observed between the coordinates of Axis1 (-0.5; 0.0) and Axis2 (-0.2 and 0.0) (Fig 1D).

**Fig 2:**
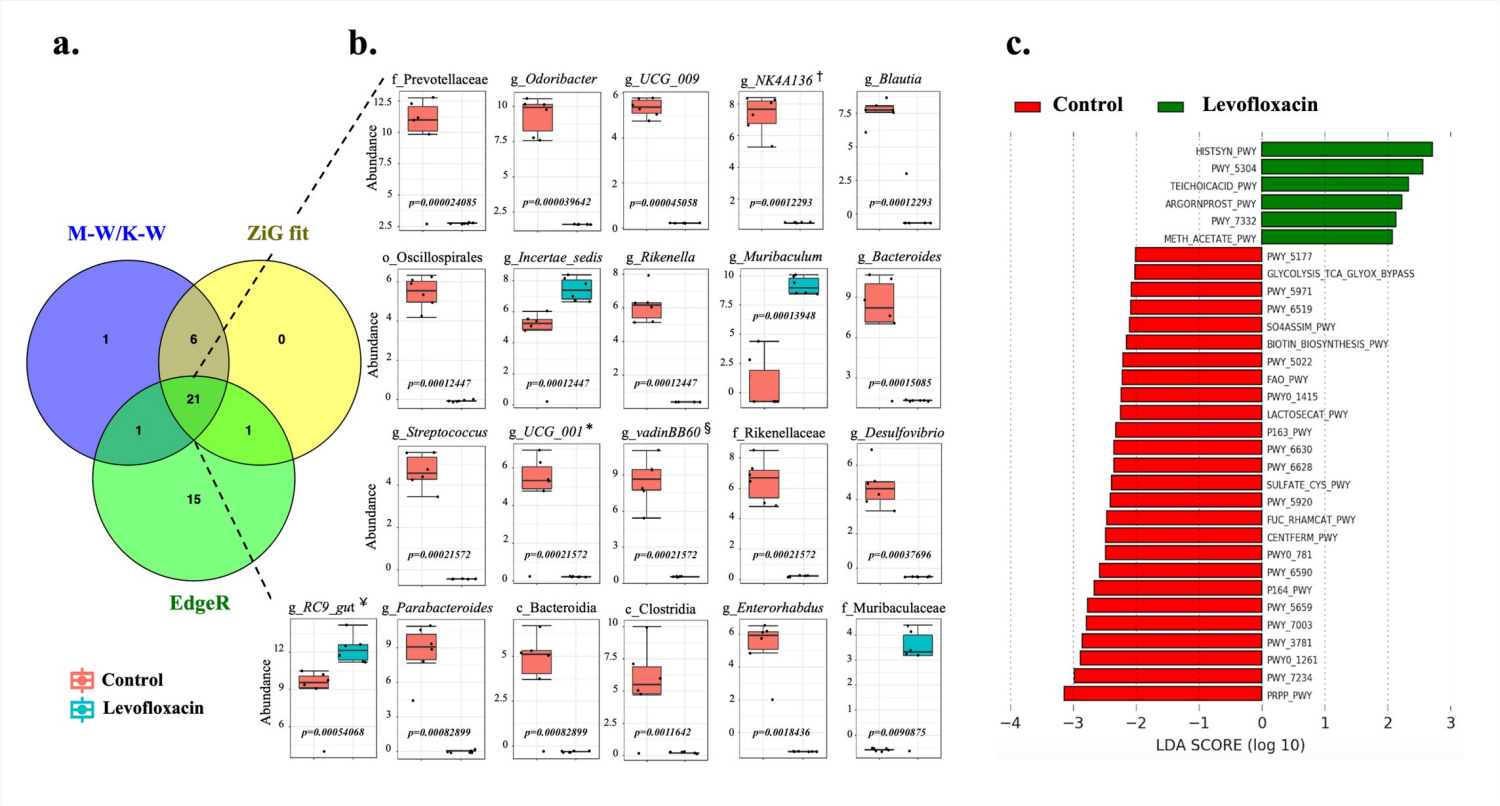
a. Venn diagram indicating the significantly different taxa in the stool microbiota of control and levofloxacin-treated mice (p<0.05) (n = 6 mice per group). Mann-Whitney/Kruskal-Wallis (M-W/K-W), Zero-inflated Gaussian fit (ZiG fit), and EdgeR analysis methods. b. Each box indicates the taxon’s abundance represented with a logarithmic transformation (Log-transformed Count). Full name obtained from Silva database ^†^: Lachnospiraceae_NK4A136_group, *: Prevotellaceae_UCG_001, ^§^: Clostridia_vadinBB60_group, ^¥^: Rikenellaceae_RC9_gut_group. The last available classification level was used when no genus-level identification was obtained. The letter before the name indicates the taxonomic level reached in identification (*i*.*e*., c: class, o: order, f: family, g: genus). c. Linear discriminant analysis effect size (LEfSe) of the metabolic pathways obtained in PICRUSt between control and levofloxacin-treated mice (LDA> 2; p<0.05). The annotation of the metabolic pathways was performed in MetaCyC, available at https://biocyc.org.

Comparisons of the control and levofloxacin-treated mice by Mann-Whitney/Kruskal-Wallis, Zero-inflated Gaussian fit, and EdgeR methods generated 29, 28, and 38 significant differences, respectively at the genus level (Fig 2A). The Venn diagram indicated that these three statistical methods had 21 significantly different taxa in common (Fig 2A, S1 and S2 Tables). 80% of the taxa (17 taxa) had higher Arel in the control group than the treated group. Taxa that showed the most remarkable differences were an unknown genus of the *Prevotellaceae* family, *Odoribacter* (*Marinifilaceae*), UCG_009 (*Butyricicoccaceae*), NK4A136_group, and *Blautia* (*Lachnospiraceae*) (Fig 2B). Seventeen taxa with high Arel in control had low or near zero Arel in the treated mice group (Fig 2B). The remaining four taxa (20%) that presented high Arel in the levofloxacin-treated group were *Incertae sedi* (*Ruminococcaceae*), *Muribaculum* (*Muribaculaceae*), RC9_gut (*Rikenellaceae*), and unknown genus of the *Muribaculacea*e family (Fig 2B).

Based on the prediction obtained with PICRUSt2, the mouse stool microbiota is associated with 348 metabolic pathways. LEfSe analysis determined that 9.1% (32) of the total routes presented significant differences (p<0.05) (Fig 2C). Of the 32 different pathways, 26 (81.25%) were present in the control group and the remaining 6 (18.75%) in the treated mice (Fig 2C). In the control group, the metabolic pathways with the lowest and highest LDA scores were glutaryl-CoA degradation (PWY-5177) and the pathway of histidine, purine, and pyrimidine biosynthesis (PRPP-PWY), respectively. On the other hand, in the levofloxacin group, the metabolic pathways with the lowest and highest LDA scores were methanogenesis from acetate (METH_ACETATE_PWY) and L-histidine biosynthesis (HISTSYN_PWY), respectively (Fig 2C). Most of the different pathways that are abundantly represented in the microbiota of the control group are absent or significantly reduced in the levofloxacin group, most showing drastic reduction except P163-PWY, PWY-5659, PWY0-781, SO4ASSIM_PWY, and SULFATE_CYS_PWY pathways. On the other hand, in the levofloxacin-treated group, the route with the highest representation for the control was PWY-7332 (S3 Table).

These results confirm that, as expected, given its antimicrobial effect, levofloxacin modified the microbiota profile, reducing bacterial abundances and diversities. Interestingly, most of the reduced bacteria belonged to genera considered proinflammatory, so the effect of the antibiotic was mainly beneficial.

### Confirmation of intestinal inflammation in the DBA/1 mice with spontaneous arthritis and the potential of levofloxacin to decrease it

To our knowledge, intestinal inflammation had not been previously described in the DBA/1 mice. However, we have confirmed through histopathology that mice with spontaneous arthritis have intestinal inflammation, which is a valuable finding because it broadens their similarities with the human SpA and gives this model higher value for investigating the relationship between the microbiota, intestinal inflammation, and the consequences in the musculoskeletal system.

**Fig 3:**
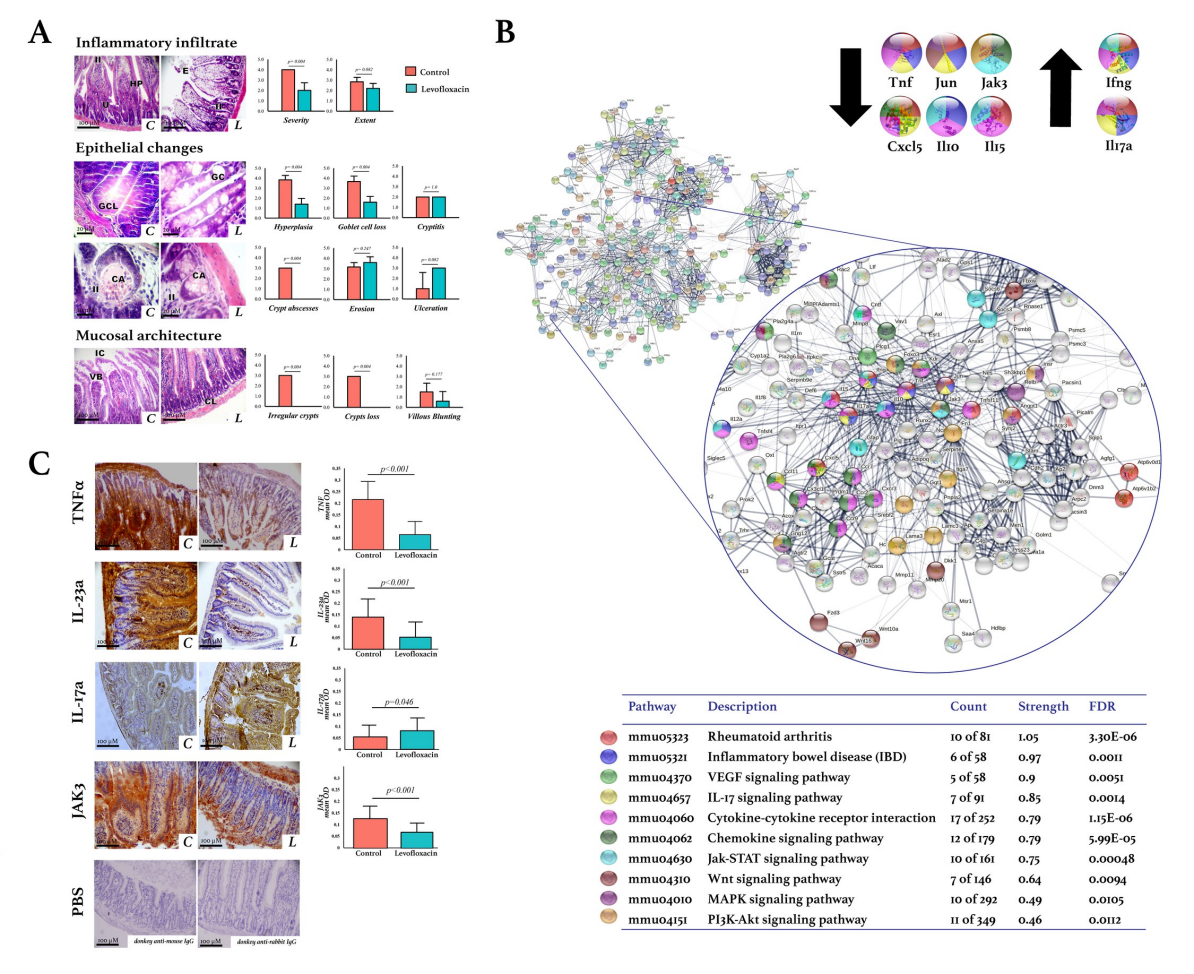
Effects of levofloxacin on the intestines of DBA/1 mice. A. Histological findings in the ileocolic junction by H&E staining (n = 6 mice per group). Inflammatory cell infiltration was scored according to severity and extent. Epithelial changes were scored by quantifying hyperplasia, goblet cell loss, crypt abscesses, erosion, and ulceration. The mucosal architecture was evaluated according to granulation tissue, irregular crypts, crypt loss, and villous blunting. The student’s t-test was used to determine statistically significant differences when p<0.05. The images were acquired with 10X and 40X amplification using a digital camera (AmScope MU1803) and an optical microscope (AxioStar Plus, Carl Zeiss). II: Inflammatory infiltrate; HP: Hyperplasia; E: Epithelial erosion; U: Epithelial ulceration; GC: Goblet cell; GCL: Goblet cell loss; CA: Crypt abscesses; IC: Irregular crypts; CL: Crypt loss; VB: Villous blunting. B. Protein-protein interactions network of differentially expressed genes (DEG). The lists of the DEG (Z-score ≥ 1.5 SD) were analyzed on the STRING and Cytoscape platforms. The primary clusters of sub-networks were obtained using the Molecular Complex Detection (MCODE) complement (cutoff = 0.4). The network was obtained in STRING with the first three clusters for up- and down-expressed genes. The KEGG signaling pathways relevant to arthritis were selected (Table) and marked with different colors. Nodes with the most significant interaction in the pathways of interest are amplified. C. Expression of TNF-α, IL-23a, IL17-a, and JAK-3 in the ileocolic junction. Immunodetection was done using streptavidin-peroxidase conjugated and DAB as the chromogen. The images were acquired with 10X amplification using a digital camera (AmScope MU1803) coupled with an optical microscope (AxioStar Plus, Carl Zeiss); the scale bar corresponds to 100 μM. The quantification was carried out with the ImageJ program and the IHC toolbox in at least 20 microscopic field images from each study subject. The DAB color was extracted from each image, and the maximum and mean gray values were obtained. Each image’s optical density (OD) was obtained with *log*_*10*_ (maximum gray value / mean gray value). The OD means, and standard deviations were calculated and graphed by the study group. The student’s t-test was used to determine statistically significant differences when p<0.05. C: Control. L: Levofloxacin. FDR: False discovery rate.

The ileocolic junctions of DBA/1 mice were histologically evaluated using H&E staining by analyzing the inflammatory infiltrate and changes in epithelium and mucosal architecture. All the variables evaluated were abnormally altered in the control group (Fig 3A), demonstrating that in untreated conditions, the DBA/1 mice that develop spontaneous arthritis also develop intestinal inflammation. Levofloxacin treatment significantly decreased the severity of inflammatory infiltrate, hyperplasia, goblet cell loss, crypt abscesses, crypt loss, and crypt irregularity, as described in Fig 3A.

Transcriptomic analysis of the ileocolic junctions showed that levofloxacin induced the differential expression of 1,956 genes; 820 were up-expressed, and 1,136 were down-expressed (Z-score ≥ 1.5 SD). The datasets resulting from the microarray were registered in the Gene Expression Omnibus (GEO)—NCBI database with the accession number: GSE216174. The primary clusters of protein subnetworks for up-expressed and down-expressed genes that resulted from STRING and Cytoscape analyses are presented in S1 Fig.

In the ileocolic junctions, the DEGs were associated with the inflammatory KEGG pathways shown in Table 1. The signaling pathways of cytokine-cytokine receptor interaction, chemokine, Jak-STAT, and rheumatoid arthritis resulted in greater strength of gene association. The KEGG pathways related to metabolic processes are shown in Table 2; the metabolism of essential fatty acids, including arachidonic, linoleic, and alpha-linoleic, are highlighted.

**Table 1:** Inflammatory signaling pathways differentially expressed by levofloxacin in the intestines, hind paws, and spines of DBA/1 mice with spontaneous arthritis.

KEGG Pathway	Description	Intestine Count (FDR): Genes	Hind paws Count (FDR): Genes	Spine Count (FDR): Genes
mmu04010	MAPK signaling pathway	10 (0.0105): Angpt1, Gng12, Insr, Jun, Kdr, Pla2g4a, Pla2g4d, Rac2, Relb, Tnf,	11 (0.0275): Akt2, Akt3, Erbb4, Gng12, Hgf, Hspa8, Igf1r, Kdr, Nfkb2, Pla2g4, Prkaca	NA
mmu04060	Cytokine-cytokine receptor interaction	17 (1.15E^-6^): Ccl11, Ccr2, Ccr7, Ccr9, Cntf, Cx3cl1, Cxcl5, Cxcr3, Ifng, Il10, Il12a Il15, Il17a, Kdr, Tnf, Tnfsf11, Tnfsf4	17 (4.41E^-5^): Ccl17, Ccl28, Ccl5, Ccr2, Ccr3, Ccr8, Cd40Ig, Cxcl12, Cxcl14, Cxcr2, Hgf, Il12a, Il17a, Kdr, Tnfrsf18, Tnfrsf9, Tnfsf11	13 (0.0015): Ccl20, Ccl3, Ccr10, Ccr2, Ccr3, Ccr9, Epo, Il10, Il12a, Il13, Il23a, Kdr, Xcl1
mmu04062	Chemokine signaling pathway	12 (5.99E^-5^): Ccl11, Ccr2, Ccr7, Ccr9, Cx3cl1, Cxcl5, Cxcr3, Foxo3, Gng12, Jak3, Rac2, Vav1	16 (4.75E^-6^): Akt2, Akt3, Ccl17, Ccl28, Ccl5, Ccr2, Ccr3, Ccr8, Cxcl12, Cxcl14, Cxcr2, Gnai3, Gng12, Gng7, Plcb2, Prkaca	15 (6.81E^-6^): Adcy7, Ccl20, Ccl3, Ccr10, Ccr2, Ccr3, Ccr9, Gnb2, Lyn, Prkaca, Rac2, Stat3, Vav1, Vav3, Xcl1
mmu04064	NF-kappa B signaling pathway	NA	8 (0.0017): Birc2, Birc3, Cd40lg Cxcl12, Lck, Nfkb2, Plau, Tnfs11	NA
mmu04066	HIF-1 signaling pathway	6 (0.0081): Angpt1, Ifng, Insr, Plcg1, Serpine1, Trf	11 (5.04E^-5^): Akt2, Akt3, Egln1, Eno1, Eno3, Hk2, Igf1r, Ldha, Tceb1, Timp1, Trf	8 (0.0019): Eif4ebp1, Epo, Ldha, Plcg1, Stat3, Tfrc, Timp1, Trf
mmu04150	mTOR signaling pathway	6 (0.0262): Atp6v1b2, Fzd3, Insr, Tnf, Wnt10a, Wnt16	NA	NA
mmu04151	PI3K-Akt signaling pathway	11 (0.0112): Angpt1, Fn1, Foxo3, Gng12, Insr, Itga4, Itga7, Jak3, Kdr, Lama3, Lamc3	12 (0.0342): Akt2, Akt3, Chrm2, Erbb4, Gng12, Gng7, Hgf, Igf1r, Kdr, Lamb1, Lpar6, Plcb2	NA
mmu04152	AMPK signaling pathway	5 (0.0415): Acaca, Adipoq, Eef2, Foxo3, Insr	7 (0.0181): Akt2, Akt3, Ccna1, Fasn, Igf1r, Pfkm	NA
mmu04310	Wnt signaling pathway	7 (0.0094): Dkk1, Fbxw11, Fxd3, Jun, Rac2, Wnt10a, Wnt16	7 (0.0313): Fzd6, Plcb2, Prkaca, Psen1, Sfrp1, Tcf7l2, Wnt3	9 (0.0031): Apc, Fzd8, Nfatc2, Prkaca, Rac2, Wnt1, Wnt7a, Wnt8b, Wnt9b
mmu04370	VEGF signaling pathway	5 (0.0051): Kdr, Pla2g4a, Pla2g4d, Plcg1, Rac2	4 (0.0416): Akt2, Akt3, Kdr, Pla2g4	6 (0.0028): Kdr, Nfatc2, Pla2g4a, Pla2g4e, Plcg1, Rac2
mmu04380	Osteoclast differentiation	6 (0.0131): Ifng, Jun, Relb, Socs3, Tnf, Tnfs11	6 (0.0411): Akt2, Akt3, Gm14548, Lck, Nfkb2, Tnfs11	NA
mmu04610	Complement and coagulation cascades	5 (0.014): C4b, Hc, Plg, Serpina1e, Serpine1	5 (0.0411): C3ar1, F13b, F2rl2, F2rl3, Plau	5 (0.0369): Cfi, Clu, F13b, Fgg, Vwf
mmu04630	Jak-STAT signaling pathway	10 (4.8E^-4^): Cntf, Gfap, Ifng, Il10, Il15, Il17a, Jak3, Socs3, Socs6, Stam	7 (0.0423): Akt2, Akt3, Cish, Il12a, Ptpn6, Stam2, Stat5a	8 (0.0141): Epo, Il10, Il12a, Il13, Il23a, Ptpn6, Stam, Stat3
mmu04650	Natural killer cell mediated cytotoxicity	5 (0.0286): Ifng, Plcg1, Rac2, Tnf, Vav1	NA	7 (0.0091): Lck, Nfatc2, Plcg1, Ptpn6, Rac2, Vav1, Vav3
mmu04657	IL-17 signaling pathway	7 (0.0014): Ccl11, Cxcl5, Ifmg, Il17a, Jun, Srsf1, Tnf	NA	NA
mmu04658	Th1 and Th2 cell differentiation	5 (0.0132): Ifng, Il12a, Jak3, Jun, Plcg1	6 (0.0143): Cd247, H2-Eb1, IL12a, Lck, Runx3, Stat5a	7 (0.003): Dll3, Il12a, Il13, Lck, Nfatc2, Plcg1, Rbpj
mmu04659	Th17 cell differentiation	5 (0.021): Ifng, Il17a, Jak3, Jun, Plcg1	7 (0.0083): Cd247, H2-Eb1, Il17a, Irf4, Lck, Runx1, Stat5a	6 (0.0174): Foxp3, Il23a, Lck, Nfatc2, Plcg1, Stat3
mmu04660	T cell receptor signaling pathway	6 (0.0078): Ifng, Il10, Jun, Plcg1, Tnf, Vav1	7 (0.0083): Akt2, Akt3, Cd247, Cd28, Cd40lg, Lck, Ptpn6	7 (0.006): Il10, Lck, Nfatc2, Plcg1, Ptpn6, Vav1, Vav3
mmu04662	B cell receptor signaling pathway	NA	NA	6 (0.0051): Lyn, Nfatc2, Ptpn6, Rac2, Vav1, Vav3,
mmu04664	Fc epsilon RI signaling pathway	6 (0.0016): Pla2g4a, Pla2g4d, Plcg1, Rac2, Tnf, Vav1	NA	8 (2.6E^-4^): Il13, Lyn, Plcg1, Rac2, Vav1, Vav3
mmu04666	Fc gamma R-mediated phagocytosis	6 (0.0042): Arpc2, Pla2g4a, Pla2g6, Plcg1, Rac2, Vav1	NA	7 (0.0028): Lyn, Plcg1, Rac2, Vav1, Vav3
mmu04668	TNF signaling pathway	5 (0.0264): Cx3cl1, Il15, Il17a, Jun, Tnf	NA	NA
mmu04672	Intestinal immune network for IgA production	4 (0.0088): Ccr9, Il10, Il15, Itga4	6 (9.9E^-4^): Aicda, Ccl28, Cd28, Cd40lg, Cxcl12, H2-Eb1	4 (0.0163): Ccr10, Ccr9, Cd80, Il10
mmu04750	Inflammatory mediator regulation of TRP channels	8 (0.0012): Alox12, Cyp4a10, Cyp4a14, Itpr1, Pla2g4a, Pla2g4d, Pla2g6, Plcg1	NA	10 (0.00033): Adcy7, Cyp2c55, Cyp2j5, Cyp4a12a, Gnas, Hrh1, Pla2g4a, Pla2g4e, Plcg1, Prkaca
mmu05321	Inflammatory bowel disease (IBD)	6 (0.0011): Ifng, Il10, Il12a, Il17a, Jun, Tnf	NA	6 (0.0028): Foxp3, Il10, Il12a, Il13, Il23a, Stat3
mmu05322	Systemic lupus erythematosus	5 (0.0162): C4b, Hc, Ifng, Il10, Tnf	5 (0.0443): Cd28, Cd40lg, Grin2b, H2-Eb1, H3f3a	NA
mmu05323	Rheumatoid arthritis	10 (3.3E^-6^): Angpt1, Atp6v0d1, Atp6v1b2, Cxcl5, Ifng, Il15, Il17a, Jun, Tnf, Tnfsf11	6 (0.0119): Ccl5, Cd28, Cxcl12, H2-Eb1, Il17a, Tnfs11	NA

**Table 2:** Metabolic signaling pathways differentially expressed by levofloxacin in the intestines, hind paws, and spines of DBA/1 mice with spontaneous arthritis.

KEGG Pathway	Description	Intestine Count (FDR): Genes	Hind paws Count (FDR): Genes	Spine Count (FDR): Genes
mmu00010	Glycolysis / Gluconeogenesis	NA	8 (2.9E^-4^): Eno1, Eno3, Hk2, Ldha, Pck1, Pfkm, Pgam1, Pgm2	NA
mmu00052	Galactose metabolism	NA	4 (0.0117): B4galt1, Hk2, Pfkm, Pgm2	NA
mmu00190	Oxidative phosphorylation	NA	8 (0.0083): Atp5o, Cox6b1, Mt-Cytb, Ndufb4, Ndufc2, Ndufv3, Uqcr11, Uqcrfs1	NA
mmu00480	Glutathione metabolism	NA	4 (0.0451): Gpx2, Gsta1, Gstt3, Mgst3	NA
mmu00562	Inositol phosphate metabolism	4 (0.03): Itpkc, Minpp1, Plcg1, Synj2	NA	NA
mmu00565	Ether lipid metabolism	4 (0.0112): Itpkc, Minpp1, Plcg1, Synj2	NA	NA
mmu00590	Arachidonic acid metabolism	11 (1.15 E^-6^): Alox12, Cyp2b13, Cyp2b9, Cyp4a10, Cyp4a14, Ephx2, Ggt1, Pla2g10, Pla2g4a, Pla2g4d, Pla2g6	NA	6 (0.0116): Cyp2c55, Cyp2j5, Cyp4a12a, Pla2g1b, Pla2g4a, Pla2g4e
mmu00591	Linoleic acid metabolism	7 (8.31 E^-5^): Cyp1a2, Cyp3a13, Cyp3a41a, Pla2g10, Pla2g4a, Pla2g4d, Pla2g6	4 (0.0283): Pla2g1b, Cyp3a16, Pla2g4a, Cyp3a25	5 (0.0065): Cyp2c55, Cyp2j5, Pla2g1b, Pla2g4a, Pla2g4e
mmu00592	alpha-Linolenic acid metabolism	5 (4.1E^-4^): Acox1, Pla2g10, Pla2g4a, Pla2g4d, Pla2g6	NA	3 (0.0264): Pla2g1b, Pla2g4a, Pla2g4e
mmu01100	Metabolic pathways	31 (8.7E^-4^): Acaca, Acox1, Aldh1a7, Alox12, Asah1, Atp5c1, Atp6v0d1, Atp6v1b2, Cyp1a2, Cyp2b13, Cyp2b9, Cyp3a13, Cyp3a41a, Cyp4a10 Cyp4a14, Ephx2, Galns, Ggt1, Hao2, Itpkc, Lama3, Minpp1, Pla2g10, Pla2g4a, Pla2g4d, Pla2g6, Plcg1, Pnpla2, Pole, Prdx6, Synj2	36 (0.0059): Atp5o, B4galt1, Cox6b1, Cycs, Cyp3a16, Cyp3a25, Ehhadh, Eno1, Eno3, Eprs, Fasn, Ftcd, Hk2, Hmgcs2, Itpa, Ldha, mt-Cytb, Mthfd2, Ndufb4, Ndufc2, Ndufv3, Nos1, Ntpcr, Pck1, Pfkm, Pgam1, Pgm2, Pla2g1b, Pla2g4, Plcb2, Pola1, Pola2, Polr1d, Polr3k, Uqcr11, Uqcrfs1	29 (0.042): Aco1, Asah1, Cup19a1, Cyp11a1, Cyp2c55, Cyp2j5, Cyp4a12a, Eprs, Gad1, Gad2, Got1, Gpt, Hexb, Kmo, Ldha, Ldhb, Ndufa6, Ndufc2, Nt5c, Pck1, Pla2g1b, Pla2g4a, Pla2g4e, Plcg1, Pole3, Pole4, Pygl, Rgn, Treh
mmu03320	PPAR signaling pathway	5 (0.0131): Acox1, Adipoq, Apoa1, Cup4a14, Cyp4a10	5 (0.039): Ehhadh, Fabp1, Fabp5, Hmgcs2, Pck1	5 (0.0342): Cyp4a12a, Lpl, Pck1, Slc27a1, Ucp1
mmu04071	Sphingolipid signaling pathway	NA	6 (0.0396): Akt2, Akt3, Gnai3, Oprd1, Plcb2, S1pr3	NA
mmu04072	Phospholipase D signaling pathway	NA	7 (0.0313): Akt2, Akt3, Cxcr2, Dnm1, Lpar6, Pla2g4, Plcb2	8 (0.0092): Adcy7, Avpr1b, Gnas, Grm3, Lpar6, Pla2g4a, Pla2g4e, Plcg1
mmu04922	Glucagon signaling pathway	NA	8 (0.0024): Akt2, Akt3, Gcg, Ldha, Pck1, Pgam1, Plcb2, Prkaca	8 (0.0017): Gcg, Gnas, Ldha, Ldhb, Pck1, Prkaca, Pygl, Sirt1
mmu04923	Regulation of lipolysis in adipocytes	NA	4 (0.039): Akt2, Akt3, Gnai3, Prkaca	4 (0.0358): Adcy7, Gnas, Npy1r, Prkaca

The resulting IPP network of DEGs in the ileocolic junctions is shown in Fig 3B. Ten of the pathways of interest in arthritis were selected (Table in Fig 3B), highlighting the Ifng, Tnf, Jun, Il17a, Kdr, and Cxcl5 genes as having the most significant interaction and relevance.

The expression levels of the TNF-α, IL-23, IL-17a, and JAK3 proteins were determined by IHC in the ileocolic junctions and were compared between the groups treated and not with levofloxacin (Fig 3C). In both groups, TNF-α was the marker most expressed, followed by IL-23a and JAK3. Levofloxacin significantly decreased the expression of these three proteins (p<0.001). Interestingly, the expression of these markers was significantly higher in the ileal epithelium for the lamina propria and regions with inflammatory infiltrate. Contrasting these three cytokines, IL-17a was increased by the effect of levofloxacin in the ileocolic junction, a finding consistent with the DNA microarray.

### The levofloxacin-induced microbiota modifications decrease peripherical and axial arthritis severity, selectively decrease cytokine expression, and modify the metabolism

**Fig 4:**
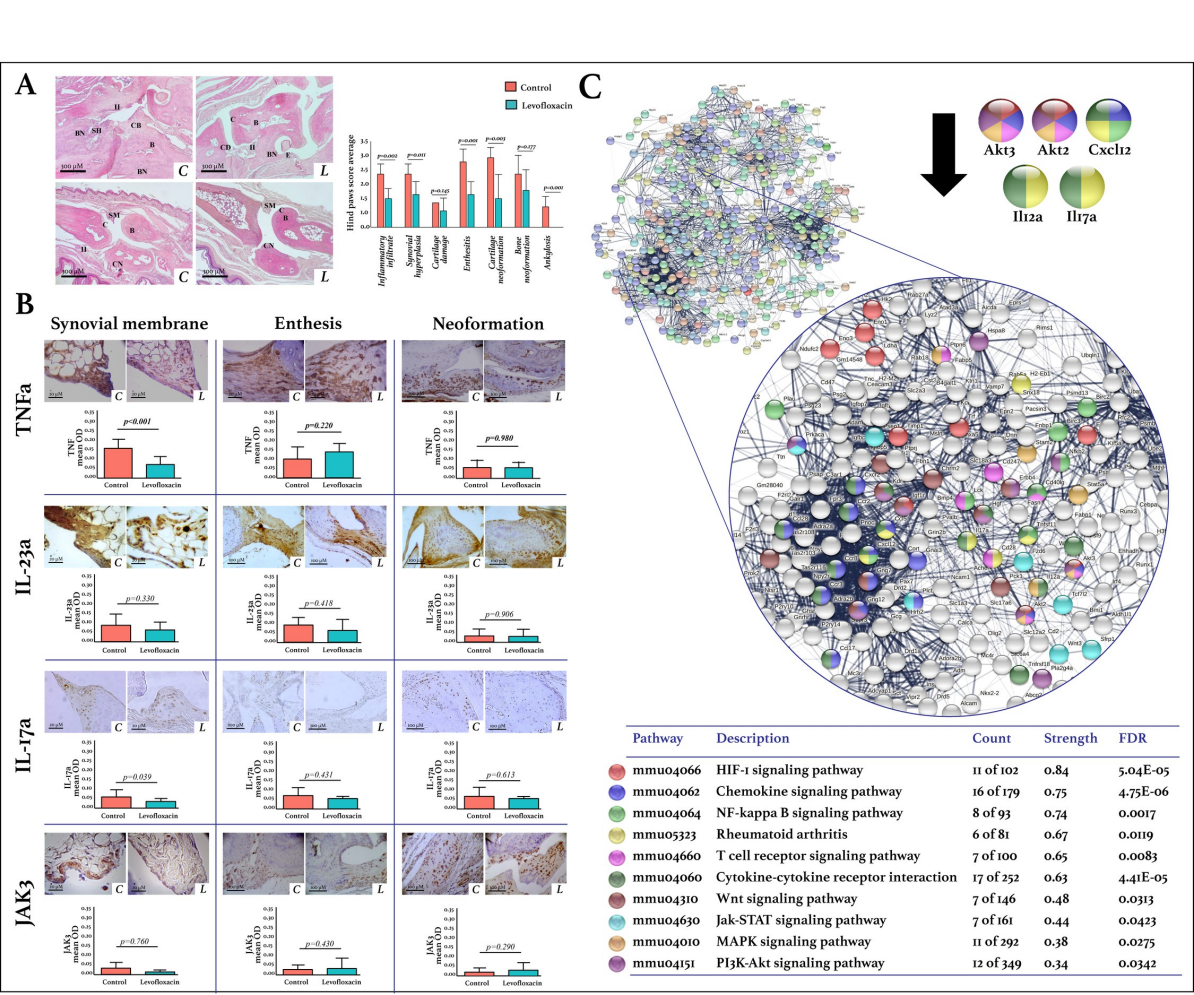
Effects of levofloxacin on the hind paws of DBA/1 mice. A. Histological findings in the hind paws by H&E staining. Inflammatory infiltrate (II), synovial membrane (SM) hyperplasia (SH), cartilage (C) damage (CD), enthesitis (E), cartilage neoformation (CN), bone (B) neoformation (BN), and ankylosis was evaluated using the semiquantitative scale: 0, absent; 1, mild; 2, moderate; or 3, severe. The mean score and standard deviation were calculated and graphed for each group (n = 6 mice per group). The student’s t-test was used to determine statistically significant differences when p<0.05. The images were acquired with 4X amplification using a digital camera (AmScope MU1803) and an optical microscope (AxioStar Plus, Carl Zeiss). B. Protein-protein interactions network of DEG. The lists of the differentially expressed genes (Z-score ≥ 1.5 SD) were analyzed on the STRING and Cytoscape platforms. The primary clusters of sub-networks were obtained using the Molecular Complex Detection (MCODE) complement (cutoff = 0.4). The network was obtained in STRING with the first three clusters of up- and down-expressed genes. The KEGG signaling pathways relevant to arthritis were selected (Table) and marked with different colors. Nodes with the most significant interaction in the pathways of interest are amplified. C. Expression of TNF-α, IL-23a, IL17-a, and JAK-3 in synovial membranes, enthesis, and neoformation areas of hind paws. Immunodetection was done using streptavidin-peroxidase conjugated and DAB as the chromogen. The images were acquired with 10X and 40X amplification using a digital camera (AmScope MU1803) and an optical microscope (AxioStar Plus, Carl Zeiss). The quantification was carried out with the ImageJ program and the IHC toolbox in at least 20 microscopic field images from each study subject. The DAB color was extracted from each image, and the maximum and mean gray values were obtained. Each image’s optical density (OD) was obtained with *log*_*10*_ (maximum gray value / mean gray value). The OD means, and standard deviations were calculated and graphed by the study group. The student’s t-test was used to determine statistically significant differences when p<0.05. C: Control. L: Levofloxacin. FDR: False discovery rate.

Levofloxacin treatment influenced not only intestinal inflammation but also altered the nature of the inflammatory process in peripheral joints. Histologically, in both the tarsal joints and the interphalangeal joints, levofloxacin-treated mice exhibited less inflammatory infiltrate, synovial hyperplasia, enthesitis, cartilage neoformation, and ankylosis compared with control mice (Fig 4A).

Transcriptomic analysis of the hind paws showed that levofloxacin induced the differential expression of 2,515 genes: 1,322 were up-expressed, and 1,193 were down-expressed. The datasets resulting from the microarray were registered in the GEO—NCBI database with the accession number: GSE216172. The primary clusters of protein subnetworks for up-expressed and down-expressed genes that resulted from STRING and Cytoscape analyses are displayed in S1 Fig.

In the hind paws of levofloxacin-treated mice, the DEGs were more strongly associated with the inflammatory KEGG pathways of cytokine-cytokine receptor interaction, chemokines, hypoxia-inducible factor (HIF)-1, and intestinal immune network for IgA production (Table 1). Regarding metabolism, pathways of lipid metabolism, peroxisome proliferator-activated receptor (PPAR), phospholipase D, and lipolysis in adipocytes were differentially expressed (Table 2). Moreover, DEGs in the hind paws were associated with carbohydrate metabolism, including glycolysis/gluconeogenesis, galactose metabolism, and oxidative phosphorylation. The IPP network resulting from DEGs in the hind paws of levofloxacin-treated mice is shown in Fig 4B. The most outstanding dysregulated mediators were the serine/threonine-protein kinases Akt2 and Akt3.

The effect of levofloxacin on the expression of TNF-α, IL-23a, IL-17a, and JAK3 in the synovial membranes, enthesis, and neoformation areas of the hind paws was evaluated by IHC (Fig 4C). In both groups, TNF-α was the most expressed marker in the hind paws, followed by IL-23, JAK3, and IL-17a. Levofloxacin induced a significant decrease in the expression of TNF-α (p<0.001), IL-17a (p = 0.034), and JAK3 (p = 0.039) in the synovial membranes; however, no differences were found in the entheses or the neoformation areas. No significant differences were found between the groups in the expression of IL-23 in any of the analyzed regions.

In the mice’s spines, levofloxacin induced the differential expression of 2,474 genes; 1,337 were up-expressed and 1,137 down-expressed. The datasets resulting from the microarray were registered in the GEO—NCBI database with the accession number: GSE216173. The primary clusters of protein subnetworks for up-expressed and down-expressed genes obtained using STRING and Cytoscape platforms are shown in S1 Fig.

**Fig 5:**
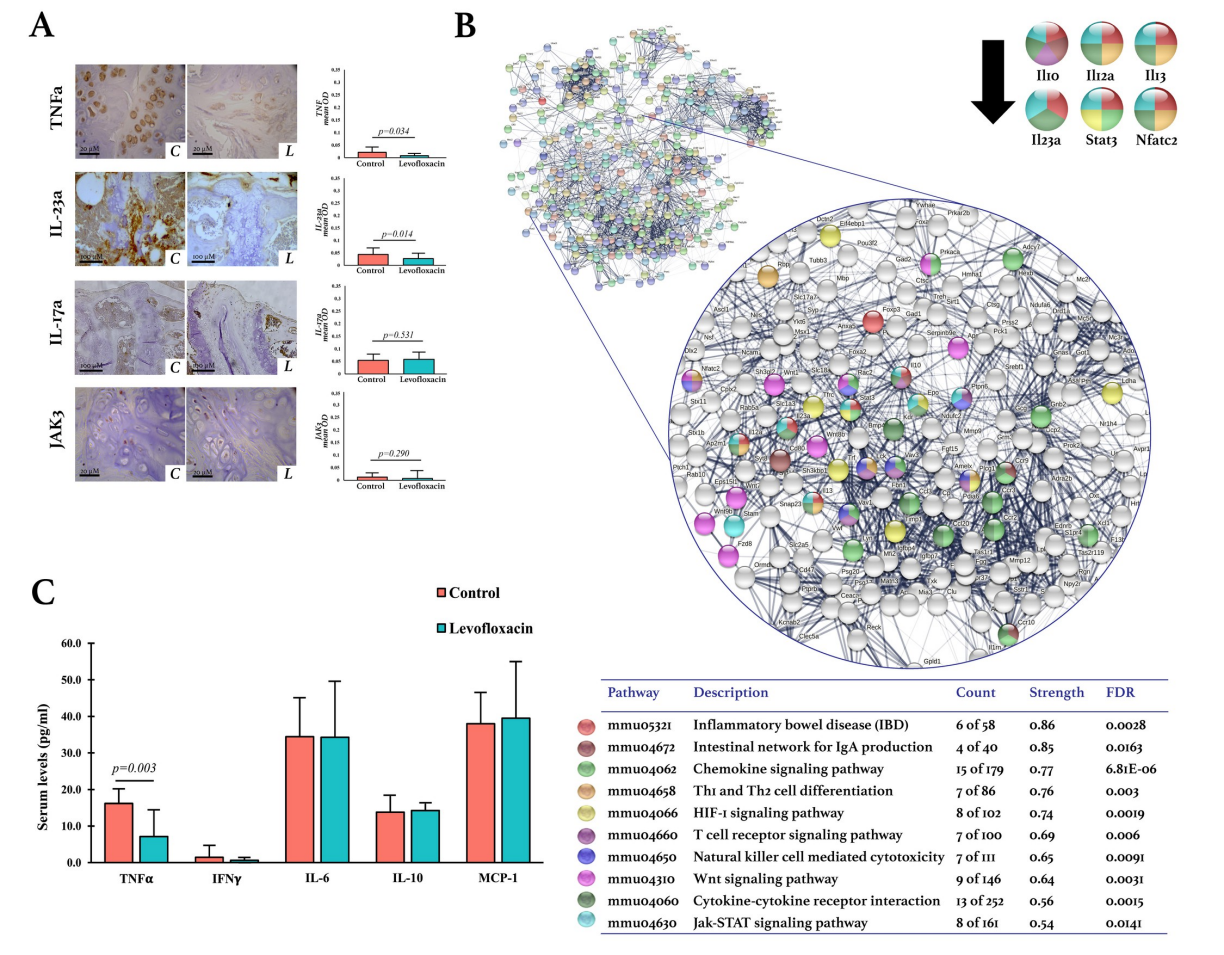
Effects of levofloxacin on the spines of DBA/1 mice. A. Protein-protein interactions network of differentially expressed genes. The lists of the differentially expressed genes (Z-score ≥ 1.5 SD) were analyzed on the STRING and Cytoscape platforms. The primary clusters of sub-networks were obtained using the Molecular Complex Detection (MCODE) complement (cutoff = 0.4). The network was obtained in STRING with the first three clusters of up- and down-expressed genes. The KEGG signaling pathways relevant to arthritis were selected (Table) and marked with different colors. Nodes with the most significant interaction in the pathways of interest are amplified. B. Expression of TNF-α, IL-23a, IL17-a, and JAK-3 in the spine. Immunodetection was done using streptavidin-peroxidase conjugated and DAB as the chromogen. The images were acquired with 10X and 40X amplification using a digital camera (AmScope MU1803) and an optical microscope (AxioStar Plus, Carl Zeiss). The quantification was carried out with the ImageJ program and the IHC toolbox in at least 20 microscopic field images from each study subject. The DAB color was extracted from each image, and the maximum and mean gray values were obtained. Each image’s optical density (OD) was obtained with *log*_*10*_ (maximum gray value / mean gray value). The OD means, and standard deviations were calculated and graphed by the study group. The student’s t-test was used to determine statistically significant differences when p<0.05. C: Control. L: Levofloxacin. FDR: False discovery rate. C. Effect of levofloxacin on serum cytokines levels in DBA/1 mice. TNF-α, IFNγ, IL-6, IL-10, and monocyte chemoattractant protein-1 (MCP-1) concentrations (pg/ml) were determined by flow cytometry in the serum of control and levofloxacin-treated mice. Bars show each cytokine’s means and standard deviations in the study groups. The student’s t-test was used to determine statistically significant differences when p<0.05.

The DEGs in the spines of levofloxacin-treated mice were associated with 17 signaling pathways of inflammation, including chemokine, Fc epsilon RI, and rheumatoid arthritis (Table 1). Regarding metabolism, the DEGs were associated with lipid metabolism pathways, including arachidonic and linoleic acid, PPAR, phospholipase D, and regulation of lipolysis in adipocytes (Table 2). The IPP network resulting from DEGs in the spines of levofloxacin-treated mice is shown in Fig 5A. In this network, relevant inflammatory mediators such as Il10, Il13, and Il12a stood out.

The effect of levofloxacin on the expression of TNF-α, IL-23, IL-17a, and JAK3 was determined by IHC in the mice’s spines. In both groups, IL-23 was the most expressed marker, followed by TNF-α, IL-17a, and JAK3. Levofloxacin induced a significant decrease in the expression of IL-23 (p = 0.014) and TNF-α (p = 0.034), while no differences were found between the groups for IL-17a and JAK3 expression (Fig 5B).

### The effects of levofloxacin-induced intestinal microbiota modifications on the inflammatory and metabolic processes are specific at the intestinal and joint levels and differ between peripheral and axial joints

Despite the overall improvement of the levofloxacin-treated mice, the specific changes in cytokines differ amongst the intestine and the peripheral and axial joints. Therefore, a more targeted comparison of gene and protein expression of inflammatory mediators was made. First, from the list of DEGs, the deregulation of the most representative inflammatory cytokines in these three sites was compared (Table 3). Excepting Il12a and Il17rc, which were down-expressed in all three tissues, the rest of the cytokines showed a heterogeneous behavior. For example, the Ifng, Jak3, and Tnf genes were only up-expressed in the ileocolic junction; Il10, Il13, and IL-23a genes were down-expressed only in the spine. Moreover, some cytokines had opposite effects between tissues, including Il17a, up-expressed in the ileocolic junction and down-expressed in the hind paws.

**Table 3:** Inflammatory cytokines differentially expressed by levofloxacin in the intestine, hind paws, and spine of DBA/1 mice.

Cytokine	Intestine	Hind paw	Spine
Ifng	-3.292	NS	NS
Il1rn	-1.859	-2.155	-2.292
Il1f5	NS	NS	1.938
Il1f6	NS	NS	-1.568
Il1f8	-1.621	NS	NS
Il4i1	NS	NS	1.674
Il8rb	NS	-1.704	NS
Il10	-1.915	NS	-1.739
Il12a	-1.669	-1.770	-2.498
Il13	NS	NS	-1.790
Il15	-1.852	NS	NS
Il17a	0.706	-1.021	NS
Il17rc	-2.312	-2.010	-2.923
Il18r1	1.974	NS	NS
Il21	1.662	NS	1.698
Il23a	NS	NS	-2.136
Il24	NS	-1.560	NS
Il27ra	NS	2.010	NS
Il34	NS	2.406	NS
Jak3	-1.511	NS	NS
Nfkb2	NS	NS	1.643
Nfkbia	2.011	NS	NS
Tnf	-2.095	NS	NS

Z-score values are shown for up-expressed (positive) and down-expressed (negative) values. Z-scores ≤ 1.5 SD were considered to have no significative effects (NS).

Subsequently, through bioinformatic analysis, the dysregulated KEGG pathways in levofloxacin-treated mice were compared between the three tissues. The pathways were classified as related to inflammation (Table 1) or metabolism (Table 2). Some inflammatory signaling pathways, including cytokine-cytokine receptor interaction, HIF-1, Wnt, vascular endothelial growth factor (VEGF), complement and coagulation cascades, Jak-STAT, Th1 and Th2, Th17, T cell receptor, and intestinal immune network for IgA production, were differentially expressed in all three tissues; however, the genes and the strength of their association were not the same (Table 1). On the other hand, some signaling pathways were only differentially expressed in one of the tissues. The IL-17, TNF-α, and mTOR signaling pathways were only found in the intestines, the NF-kappa B signaling pathway in the hind paws, and the B cell receptor signaling pathway in the spines.

The metabolism-related KEGG pathways differentially expressed in levofloxacin-treated mice also showed heterogeneity in genes and association strength between the ileocolic junctions, hid paws, and spines (Table 2). In all three tissues, DEGs were associated with pathways of lipid metabolism. However, only in the hind paws, pathways of carbohydrate metabolism and aerobic respiration were associated.

The effects of levofloxacin on tissue expression of TNF-α, IL-23, IL-17a, and JAK3 were also different between the three tissues (Figs 3C, 4C and 5B). TNF-α, IL-23, and JAK3 were decreased by levofloxacin in the intestine, while IL-17a was increased. TNF-α, IL-17a, and JAK3 expression were reduced by levofloxacin in the synovial membranes of the hind paws, an effect that was not found in entheses and the areas of neoformation. In contrast, IL-23 was decreased in the spinal joints, not the hind paws.

Finally, to assess the effect of levofloxacin-induced intestinal microbiota modifications on systemic inflammation, serum cytokine levels were evaluated by flow cytometry (Fig 5C). Levofloxacin significantly decreased the concentration of TNF-α, while the levels of IFNγ, IL-6, IL-10, and MCP-1 were not affected by the antibiotic.

## Discussion

The present study assessed the effects of levofloxacin in a DBA/1 mice model with spontaneous arthritis. Levofloxacin significantly modified the gut microbiota, reduced the abundance of pro-inflammatory bacteria, and increased the anti-inflammatory activity. Moreover, levofloxacin reduced intestinal inflammation, which was confirmed using a structured histological analysis and DNA microarray transcriptome and bioinformatic analyses. Reduction of intestinal inflammation was reaffirmed by the decrease in expression of relevant cytokines and mediators (TNF-α, IL-23, and JAK3) in biopsy samples examined with IHC. Levofloxacin also reduced the inflammatory processes in the hind paws and spine, as evidenced by genetic and histopathological analyses. The present study describes intestinal inflammation in a DBA/1 mouse with spontaneous arthritis, which, to our knowledge, has not been done.

Microbiome and dysbiosis have increasingly been recognized as potential triggers and/or drivers of the inflammatory processes in SpA [18–21]. Reactive arthritis (ReA) is the best example of acute bacterial infection resulting in chronic inflammatory disease, such as ankylosing spondylitis (AS). Follow-ups of patients with food poisoning caused by arthritogenic bacteria confirm that a proportion of infected individuals develop complete ReA; a subset of these patients evolve to AS. However, several rodent models fail to develop SpA if raised in germ-free conditions suggesting that the microbiota is critical for the development of arthritis [12].

DBA/1 mice with spontaneous arthritis were selected in the present study because they are similar to human SpA and provide insight into the effect of microbiota modification on arthritis. Development of spontaneous arthritis in the DBA/1 mice only requires the close confinement of several unrelated males; no external immunization is required [22]. Therefore, the model primarily relies on the genetic background of the strain. Studies exploring the effects of dysbiosis in DBA/1 mice are scarce. Current research includes the collagen-induced arthritis (CIA) model (rheumatoid arthritis model) [38] and a seizure model [39]. In the CIA model of DBA/1 mice, the gut microbiota undergoes several changes as arthritis sets in and progresses; bacterial diversity differs between mice susceptible or resistant to arthritis. The use of a broad-spectrum antimicrobial cocktail (ampicillin, neomycin, vancomycin, and metronidazole) in CIA models modifies the microbiota and reduces the arthritis severity, which was also seen in our study. A decrease in *Muribaculaceae* and *Bacteroidetes* and an increase in *Ruminococcaceae*, *Lachnospiraceae*, and *Desulfovibrinocaceae* have been reported in this model with antibiotic use [15,16,25].

The present study showed that in spontaneous arthritis in DBA/1 mice, levofloxacin-induced dysbiosis decreased the number of pro-inflammatory gut bacteria, including *Prevotellaceae*, *Blautia*, *Odoribacter*, *Rikenella*, *Bacteroides*, *Desulfovibrio*, *Streptococcus*, *Parabacterioides*, *Clostridia*, and *Oscillospirales*. Conversely, levofloxacin increased the number of *Muribacululm* bacteria, which are considered to have anti-inflammatory properties. *Prevotella* is associated with dysbiosis and the pathogenesis of arthritis. Studies in patients with AS have shown that *Prevotella* could be increased or decreased in the gut microbiota; however, its pro-inflammatory potential is widely recognized [40,41]. Moreover, in HLA-B27-positive transgenic rats, *Prevotella* dysregulates the key pathogenic cytokines, including IL-17, IL-23, and TNF-α [42,43]. *Blautia* [44], *Odoribacter* [45], *Rikinella* [43,46], *Bacteroides* [47,48], *Parabacteroides* [17,49], *Desulfovibrio* [50,51], *Streptococcus* [52–55], and *Clostridia* [56–59] are associated with the dysbiosis in patients with SpA and/or in animal models of the human disease. Most of these bacteria primarily have pro-inflammatory potential; *Desulfovibrio* and *Parabacteroides* [60,61] also have anti-inflammatory potential.

*Muribacululm*, which increased with levofloxacin administration in the present study, is an anaerobic bacteria, previously known as S24-7 family, that belongs to the phylum of Bacteroidetes [62]. *Muribacululm* has the potential to be protective against arthritis [15,16]. Specifically, in the DBA/1 mouse model of CIA, a decrease in the number of *Muribacululm* bacteria was associated with the onset and progression of arthritis [16,25]. *Muribaculaceae* produces short-chain fatty acids (butyrate and propionate) as products of vegetal fiber digestion; these fatty acids enhance the epithelial barrier function [63,64]. Furthermore, this bacterium has been consistently described as an anti-inflammatory agent in the murine intestine [65–72], where its anti-inflammatory action is partly due to the reduction of oxidative stress [73].

In the present study, we demonstrated that the effect of levofloxacin on the transcriptome and tissue expression of inflammatory mediators differs in the intestine, the hind paws, and the spines of DBA/1 mice with spontaneous arthritis. Although there was a significant decrease in the expression of genes, signaling pathways, and inflammatory proteins (Tables 1 and 3), the expressed mediators differed between the regions. In mice not treated with the antibiotic, TNF-α and JAK3 levels were higher in the intestine than in the joints, IL-17 levels were higher in the hind paws than in the intestine, and IL-23 levels were higher in the spine than in the hind paws and the intestine. Moreover, the cytokines were downregulated in the synovium but not in the entheses or bone neoformation areas in the hind paws.

Anti-TNF agents, the first-line drugs used in SpA, are effective in all articular and extra-articular manifestations of SpA. However, subsequent therapeutic agents have succeeded only in some domains. The strict efficacy profile of the targets (IL-12, IL-23, and IL-17) has helped us to better understand the complexity of SpA pathogenesis; specific cytokines play different roles in particular structures and organs, or at different stages of the disease. This could explain the poor response to the blockade of IL-12/23 in axial disease and IL-17 in intestinal inflammation or uveitis. The specific cytokine profiles in the hind paws and the spine that were detected in our study correspond to the nature of human SpA. Treatments that are beneficial for peripheral arthritis or the skin (cs-DMARDs, anti-IL23, ustekinumab, and abatacept) are not as beneficial for the spine, which remains the most refractory. If we further investigate specific cells producing specific cytokines in a particular tissue, it is evident that several tissue types produce cytokines, as noted in our IHC analysis.

In the gut, epithelial cells (enterocytes) express most of the local TNF-α, IL-17, IL-23, and JAK3; in the spine and hind paws, chondrocytes, osteocytes, and tenocytes produce them in abundance. In some cases, osteocytes from proliferating bone are enriched with these cytokines, compared to quiescent bone. This cytokine production at the cell level has a more direct role in the pathogenic process; the tissue cells confront the aggressive stimuli, and thus are the ones to respond. In the gut, enterocytes form the barrier to the bacterial structures and products; the synovial-entheseal-bone structures in the joints deal with mechanical stress and hypoxia. This also suggests that our therapies target the tissue cells. Tissular cells’ primary role as orchestrators of the inflammatory process, by controlling the release of fine-tuning immune mediators (and not merely the passive release of damage-associated molecular patterns), has been previously suggested by us under the logic of Polly Matzinger’s Danger model [74]. Our results in this experiment confirm this hypothesis to some extent.

Our study showed that levofloxacin alters cellular metabolism in the intestine and joints in addition to modifying the inflammatory process. Dysregulation of lipid metabolism, mainly of essential fatty acids, was observed in all three tissues. Fatty acids are potent organic compounds that not only provide energy in nutrient-deficient states, but are also involved in several essential signaling cascades. Different fatty acids and their derivatives affect various cells within the joint, including chondrocytes, osteoblasts, osteoclasts, and synoviocytes. Furthermore, an unbalanced fat composition increases the risk of developing multiple musculoskeletal diseases [75].

DNA microarrays confirmed the dysregulation of the Wnt signaling pathway, whose functions are closely related to bone homeostasis; its dysregulation has been demonstrated in SpA [76]. We found dysregulation of several genes involved in preosteoblast migration and bone proliferation restriction (Apc [77,78]), mechanical stimulation (Fzd8 [79]), non-canonical Wnt-osteogenesis (Fzd6 [80]), cell differentiation, and carcinogenesis (Nfatc2 [81–83]), and abnormal bone proliferation (Prkaca [84]). Wnt signaling is not limited to articular tissue. Wnt is the fundamental pathway involved in intestinal homeostasis; it is crucial to maintain an undifferentiated, multipotent subpopulation to generate the different cell strains that can repopulate the intestinal epithelium and preserve and renew the crypt’s structure [85]. In the present study, several Wnt-related genes, including Dkk1, Rac2, Wnt10a, and Wnt16, were dysregulated in the intestine of mice. Dkk1 is increased in the serum and intestinal mucosa of patients with Crohn’s disease, and their levels correlate with those of erythrocyte sedimentation rate and C-reactive protein. Infliximab reportedly reduces the concentrations of Dkk1 in the intestine and the serum [86]. Thus, the Wnt is a pathway linked to diverse pathological scenarios at the intestinal and systemic levels.

We recognize limitations in our present investigation that prevent us from reaching more conclusive results. Our experimental design consisted of administering the antibiotic for 20 weeks, the time described in this model to reach 100% disease incidence. However, some of the early mediators of the disease process linking the gut-joint axis are likely being left out of our analysis. On the other hand, although we have confirmed intestinal inflammation in the DBA/1 mice with spontaneous arthritis, our experimental design did not allow us to determine the order of pathological events, including intestinal dysbiosis, intestinal inflammation, and arthritis. Another key aspect that has been left out of the scope of this study is the exploration of the factors that influence the differential response to the antibiotic found between the axial and peripheral joints, which remains to be elucidated.

## Conclusions

Like many others, our results confirm that controlled microbiota changes could be a potential therapy for rheumatic diseases. This can be achieved either with diet changes, loading of specific beneficial bacteria, or using drugs that modify the bacterial proportions. The list of dietary and other interventions that help control dysbiosis-induced inflammation is beyond the scope of this manuscript but remains a promising field of study.

Although antibiotics’ effects on the microbiota as a whole are unpredictable, their use as adjuvants in diseases associated with dysbiosis and refractoriness to current therapies to selectively alter the relative abundance of inconvenient bacteria (i.e., pro-inflammatory) has great potential. More clinical trials with plausible and low-risk interventions are required; methodologies for analyzing the microbiota and its potential effects on the host are increasingly accessible.

## Supporting information

S1 Fig Primary clusters of protein subnetworks for up-expressed and down-expressed genes in the intestines, hind paws, and spines of DBA/1 mice with spontaneous arthritis treated with levofloxacin. The lists of the differentially expressed genes (Z-score ≥ 1.5 SD) were analyzed on the STRING database and Cytoscape platform. The primary clusters of sub-networks were obtained using the Molecular Complex Detection (MCODE) complement (cutoff = 0.4). The network of each cluster was obtained in STRING for up- and down-expressed genes. Tables shows the KEGG signaling pathways associated with each cluster.
(DOCX)

S1 Table Post hoc analysis with Mann-Whitney/Kruskal-Wallis, Zero-inflated Gaussian fit, and EdgeR methods. Statistical information obtained in the analysis of the microbiota through sequencing.
(XLSX)

S2 Table Venn diagram intersections. Statistical information obtained in the analysis of the microbiota through sequencing.
(XLSX)

S3 Table Metabolic pathways and LDA scores (MetaCyC Annotation) in Microbiota stool. Statistical information obtained in the analysis of the microbiota through sequencing.
(XLSX)

S1 Graphical abstract (TIF)
