# Supplementary material for: Levofloxacin induces differential effects in the transcriptome between the gut, peripheral and axial joints in the Spondyloarthritis DBA/1 mice: Improvement of intestinal dysbiosis and the overall inflammatory process
Source: PLoS One. 2023 Feb 2;18(2):e0281265. doi: 10.1371/journal.pone.0281265 (PMC9894406; doi:10.1371/journal.pone.0281265)
Supplement: S1 Fig — The lists of the differentially expressed genes (Z-score ≥ 1.5 SD) were analyzed on the STRING database and Cytoscape platform. The primary clusters of sub-networks were obtained using the Molecular Complex Detection (MCODE) complement (cutoff = 0.4). The network of each cluster was obtained in STRING for up- and down-expressed genes. Tables shows the KEGG signaling pathways associated with each cluster. (DOCX) [file pone.0281265.s001.docx]

**INTESTINE**

**UP-EXPRESSED**

**CLUSTER 1**


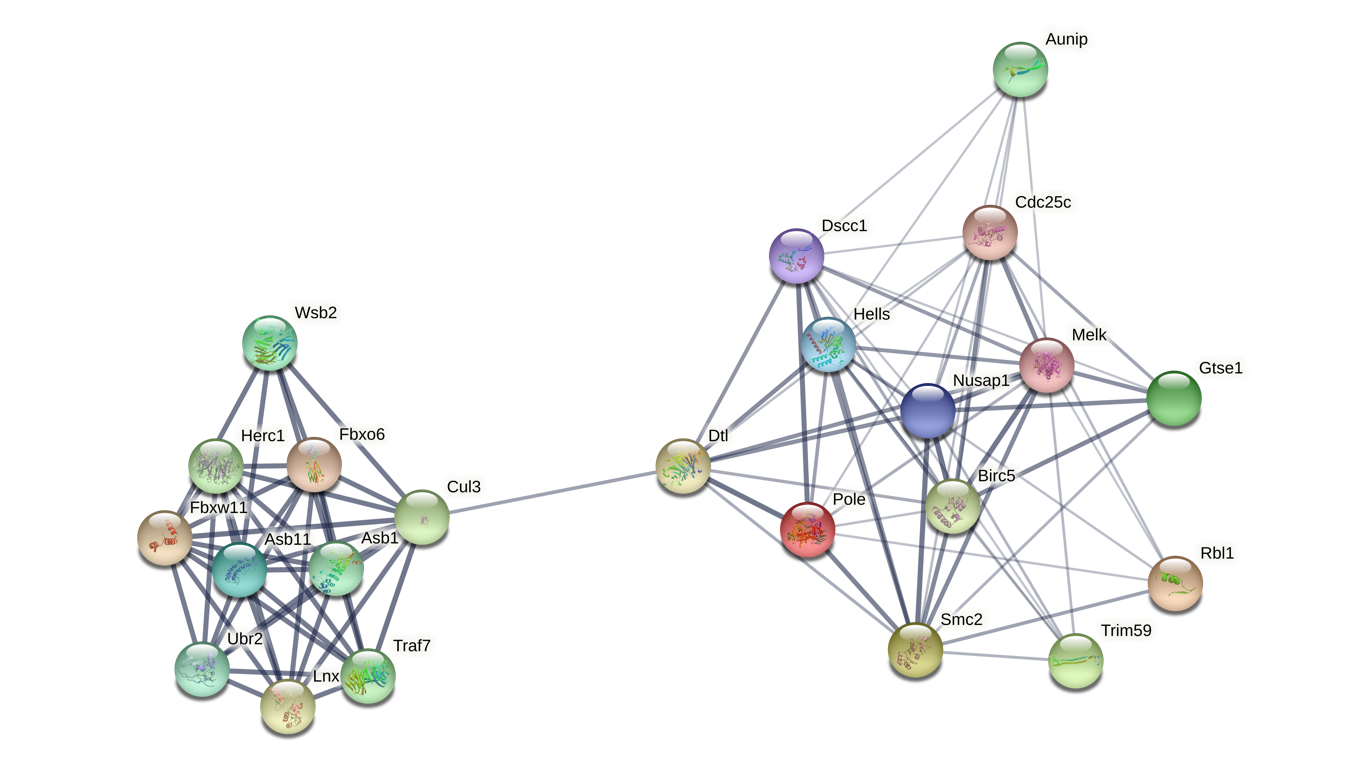


number of nodes: 23

number of edges: 100

average node degree: 8.7

avg. local clustering coefficient: 0.877

expected number of edges: 11

PPI enrichment p-value: < 1.0E^-16^

|  | **KEGG Pathways** |  |  |  |
| --- | --- | --- | --- | --- |
| ***pathway*** | ***description*** | ***count in network*** | ***strength*** | ***false discovery rate*** |
| [mmu04340](https://www.kegg.jp/kegg-bin/show_pathway?mmu04340) | Hedgehog signaling pathway | 2 of 44 | 1.64 | 0.0146 |
| [mmu04120](https://www.kegg.jp/kegg-bin/show_pathway?mmu04120) | Ubiquitin mediated proteolysis | 3 of 137 | 1.32 | 0.0113 |

**INTESTINE**

**UP-EXPRESSED**

**CLUSTER 2**

**
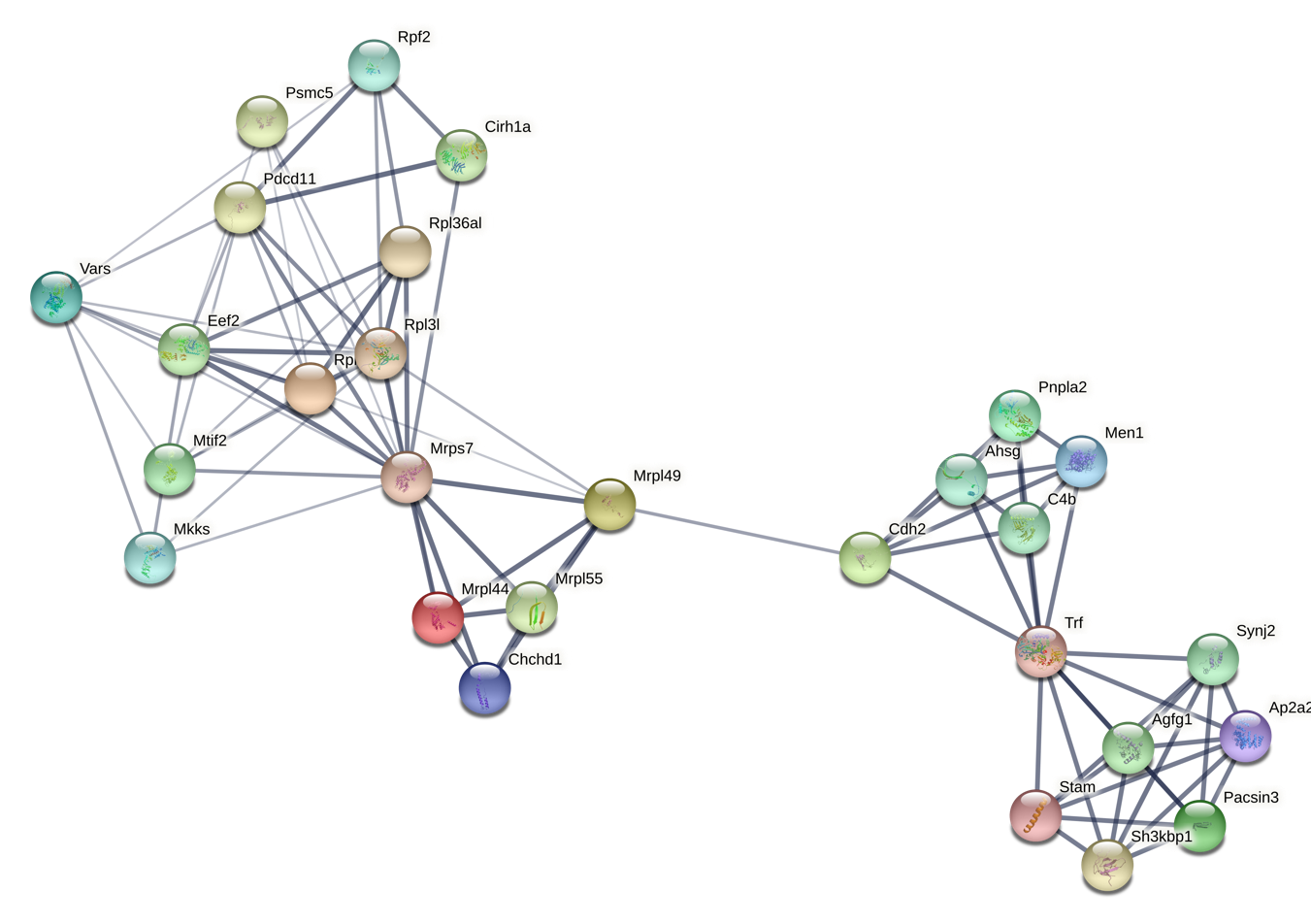
**

number of nodes: 28

number of edges: 90

average node degree: 6.43

avg. local clustering coefficient: 0.806

expected number of edges: 19

PPI enrichment p-value: < 1.0E^-16^

**NO KEGG PATHWASY ASSOCIATED**

**INTESTINE**

**UP-EXPRESSED**

**CLUSTER 3**


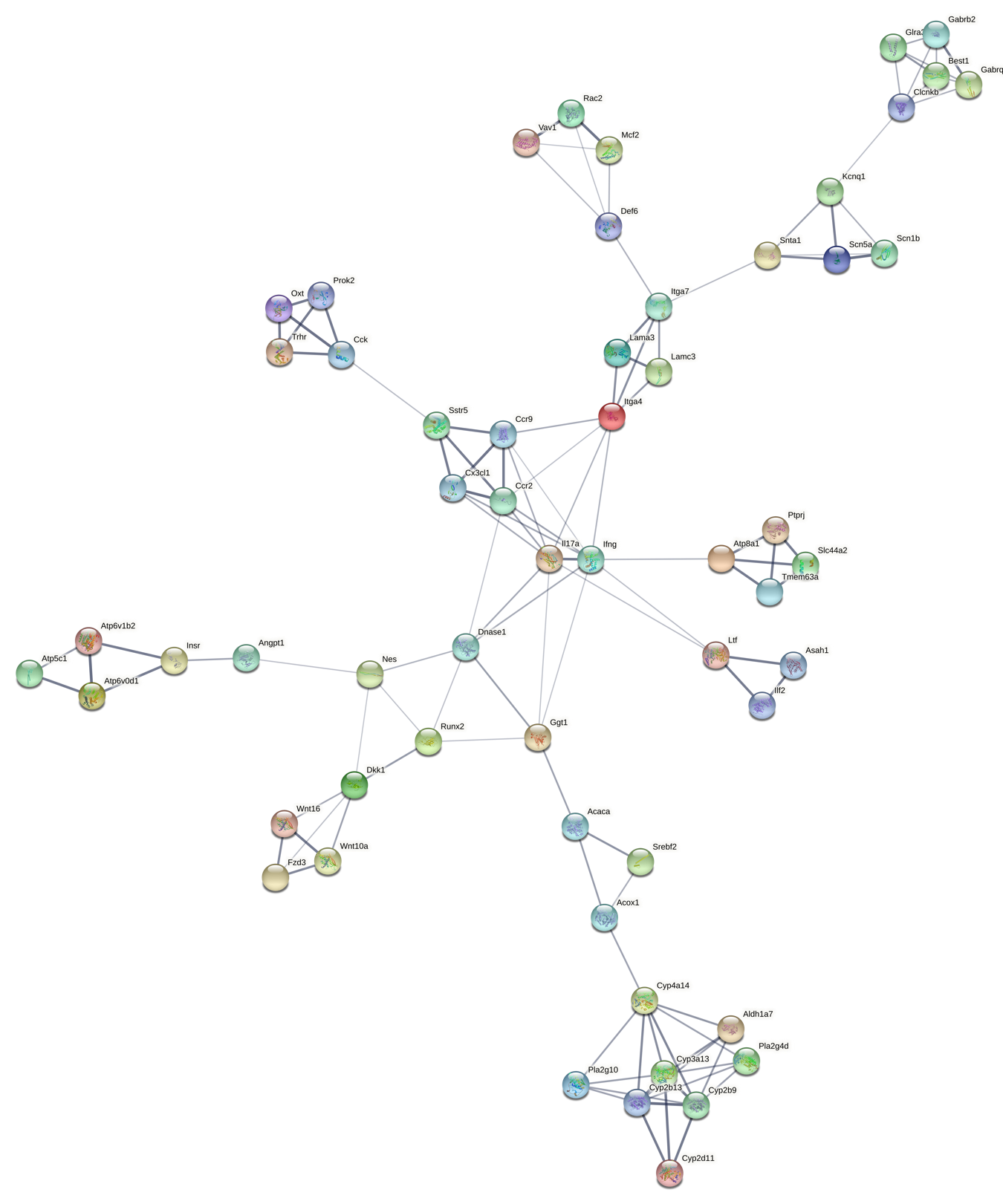


number of nodes: 58

number of edges: 118

average node degree: 4.07

avg. local clustering coefficient: 0.736

expected number of edges: 30

PPI enrichment p-value: < 1.0E^-16^

|  | **KEGG Pathways** |  |  |  |
| --- | --- | --- | --- | --- |
| ***pathway*** | ***description*** | ***count in network*** | ***strength*** | ***false discovery rate*** |
| [mmu00592](https://www.kegg.jp/kegg-bin/show_pathway?mmu00592) | alpha-Linolenic acid metabolism | 3 of 24 | 1.68 | 0.00096 |
| [mmu04966](https://www.kegg.jp/kegg-bin/show_pathway?mmu04966) | Collecting duct acid secretion | 3 of 27 | 1.63 | 0.00096 |
| [mmu00590](https://www.kegg.jp/kegg-bin/show_pathway?mmu00590) | Arachidonic acid metabolism | 6 of 88 | 1.41 | 2.40e-05 |
| [mmu00591](https://www.kegg.jp/kegg-bin/show_pathway?mmu00591) | Linoleic acid metabolism | 3 of 48 | 1.38 | 0.0033 |
| [mmu05323](https://www.kegg.jp/kegg-bin/show_pathway?mmu05323) | Rheumatoid arthritis | 5 of 81 | 1.37 | 0.00015 |
| [mmu00830](https://www.kegg.jp/kegg-bin/show_pathway?mmu00830) | Retinol metabolism | 5 of 89 | 1.33 | 0.00017 |
| [mmu05033](https://www.kegg.jp/kegg-bin/show_pathway?mmu05033) | Nicotine addiction | 2 of 40 | 1.28 | 0.0213 |
| [mmu04672](https://www.kegg.jp/kegg-bin/show_pathway?mmu04672) | Intestinal immune network for IgA production | 2 of 40 | 1.28 | 0.0213 |
| [mmu04512](https://www.kegg.jp/kegg-bin/show_pathway?mmu04512) | ECM-receptor interaction | 4 of 81 | 1.27 | 0.00096 |
| [mmu00140](https://www.kegg.jp/kegg-bin/show_pathway?mmu00140) | Steroid hormone biosynthesis | 4 of 83 | 1.26 | 0.00096 |
| [mmu05217](https://www.kegg.jp/kegg-bin/show_pathway?mmu05217) | Basal cell carcinoma | 3 of 63 | 1.26 | 0.0052 |
| [mmu04664](https://www.kegg.jp/kegg-bin/show_pathway?mmu04664) | Fc epsilon RI signaling pathway | 3 of 66 | 1.24 | 0.0056 |
| [mmu00565](https://www.kegg.jp/kegg-bin/show_pathway?mmu00565) | Ether lipid metabolism | 2 of 45 | 1.23 | 0.0239 |
| [mmu04520](https://www.kegg.jp/kegg-bin/show_pathway?mmu04520) | Adherens junction | 3 of 71 | 1.21 | 0.0064 |
| [mmu00071](https://www.kegg.jp/kegg-bin/show_pathway?mmu00071) | Fatty acid degradation | 2 of 50 | 1.18 | 0.0258 |
| [mmu01212](https://www.kegg.jp/kegg-bin/show_pathway?mmu01212) | Fatty acid metabolism | 2 of 51 | 1.17 | 0.0259 |
| [mmu04913](https://www.kegg.jp/kegg-bin/show_pathway?mmu04913) | Ovarian steroidogenesis | 2 of 57 | 1.13 | 0.0303 |
| [mmu05321](https://www.kegg.jp/kegg-bin/show_pathway?mmu05321) | Inflammatory bowel disease (IBD) | 2 of 58 | 1.12 | 0.0306 |
| [mmu04370](https://www.kegg.jp/kegg-bin/show_pathway?mmu04370) | VEGF signaling pathway | 2 of 58 | 1.12 | 0.0306 |
| [mmu04310](https://www.kegg.jp/kegg-bin/show_pathway?mmu04310) | Wnt signaling pathway | 5 of 146 | 1.11 | 0.00096 |
| [mmu04150](https://www.kegg.jp/kegg-bin/show_pathway?mmu04150) | mTOR signaling pathway | 5 of 152 | 1.1 | 0.00096 |
| [mmu05204](https://www.kegg.jp/kegg-bin/show_pathway?mmu05204) | Chemical carcinogenesis | 3 of 92 | 1.09 | 0.0117 |
| [mmu04721](https://www.kegg.jp/kegg-bin/show_pathway?mmu04721) | Synaptic vesicle cycle | 2 of 62 | 1.09 | 0.0333 |
| [mmu04510](https://www.kegg.jp/kegg-bin/show_pathway?mmu04510) | Focal adhesion | 6 of 195 | 1.07 | 0.00040 |
| [mmu05231](https://www.kegg.jp/kegg-bin/show_pathway?mmu05231) | Choline metabolism in cancer | 3 of 98 | 1.07 | 0.0131 |
| [mmu04916](https://www.kegg.jp/kegg-bin/show_pathway?mmu04916) | Melanogenesis | 3 of 98 | 1.07 | 0.0131 |
| [mmu05140](https://www.kegg.jp/kegg-bin/show_pathway?mmu05140) | Leishmaniasis | 2 of 65 | 1.07 | 0.0357 |
| [mmu04066](https://www.kegg.jp/kegg-bin/show_pathway?mmu04066) | HIF-1 signaling pathway | 3 of 102 | 1.05 | 0.0134 |
| [mmu05146](https://www.kegg.jp/kegg-bin/show_pathway?mmu05146) | Amoebiasis | 3 of 105 | 1.04 | 0.0140 |
| [mmu04662](https://www.kegg.jp/kegg-bin/show_pathway?mmu04662) | B cell receptor signaling pathway | 2 of 69 | 1.04 | 0.0391 |
| [mmu04062](https://www.kegg.jp/kegg-bin/show_pathway?mmu04062) | Chemokine signaling pathway | 5 of 179 | 1.03 | 0.0013 |
| [mmu05145](https://www.kegg.jp/kegg-bin/show_pathway?mmu05145) | Toxoplasmosis | 3 of 107 | 1.03 | 0.0143 |
| [mmu05412](https://www.kegg.jp/kegg-bin/show_pathway?mmu05412) | Arrhythmogenic right ventricular cardiomyopathy (ARVC) | 2 of 71 | 1.03 | 0.0405 |
| [mmu04650](https://www.kegg.jp/kegg-bin/show_pathway?mmu04650) | Natural killer cell mediated cytotoxicity | 3 of 111 | 1.01 | 0.0153 |
| [mmu04670](https://www.kegg.jp/kegg-bin/show_pathway?mmu04670) | Leukocyte transendothelial migration | 3 of 115 | 1.0 | 0.0163 |
| [mmu05410](https://www.kegg.jp/kegg-bin/show_pathway?mmu05410) | Hypertrophic cardiomyopathy (HCM) | 2 of 82 | 0.97 | 0.0500 |
| [mmu04270](https://www.kegg.jp/kegg-bin/show_pathway?mmu04270) | Vascular smooth muscle contraction | 3 of 125 | 0.96 | 0.0198 |
| [mmu00190](https://www.kegg.jp/kegg-bin/show_pathway?mmu00190) | Oxidative phosphorylation | 3 of 129 | 0.95 | 0.0210 |
| [mmu04726](https://www.kegg.jp/kegg-bin/show_pathway?mmu04726) | Serotonergic synapse | 3 of 130 | 0.94 | 0.0210 |
| [mmu04014](https://www.kegg.jp/kegg-bin/show_pathway?mmu04014) | Ras signaling pathway | 5 of 228 | 0.92 | 0.0033 |
| [mmu04550](https://www.kegg.jp/kegg-bin/show_pathway?mmu04550) | Signaling pathways regulating pluripotency of stem cells | 3 of 137 | 0.92 | 0.0221 |
| [mmu04261](https://www.kegg.jp/kegg-bin/show_pathway?mmu04261) | Adrenergic signaling in cardiomyocytes | 3 of 140 | 0.91 | 0.0228 |
| [mmu05165](https://www.kegg.jp/kegg-bin/show_pathway?mmu05165) | Human papillomavirus infection | 7 of 335 | 0.9 | 0.00072 |
| [mmu04024](https://www.kegg.jp/kegg-bin/show_pathway?mmu04024) | cAMP signaling pathway | 4 of 194 | 0.89 | 0.0111 |
| [mmu05224](https://www.kegg.jp/kegg-bin/show_pathway?mmu05224) | Breast cancer | 3 of 146 | 0.89 | 0.0243 |
| [mmu05226](https://www.kegg.jp/kegg-bin/show_pathway?mmu05226) | Gastric cancer | 3 of 148 | 0.89 | 0.0246 |
| [mmu04060](https://www.kegg.jp/kegg-bin/show_pathway?mmu04060) | Cytokine-cytokine receptor interaction | 5 of 252 | 0.88 | 0.0044 |
| [mmu04390](https://www.kegg.jp/kegg-bin/show_pathway?mmu04390) | Hippo signaling pathway | 3 of 153 | 0.87 | 0.0257 |
| [mmu04810](https://www.kegg.jp/kegg-bin/show_pathway?mmu04810) | Regulation of actin cytoskeleton | 4 of 208 | 0.86 | 0.0131 |
| [mmu04934](https://www.kegg.jp/kegg-bin/show_pathway?mmu04934) | Cushing's syndrome | 3 of 156 | 0.86 | 0.0259 |
| [mmu04080](https://www.kegg.jp/kegg-bin/show_pathway?mmu04080) | Neuroactive ligand-receptor interaction | 5 of 284 | 0.83 | 0.0064 |
| [mmu05225](https://www.kegg.jp/kegg-bin/show_pathway?mmu05225) | Hepatocellular carcinoma | 3 of 168 | 0.83 | 0.0302 |
| [mmu04151](https://www.kegg.jp/kegg-bin/show_pathway?mmu04151) | PI3K-Akt signaling pathway | 6 of 349 | 0.82 | 0.0033 |
| [mmu05205](https://www.kegg.jp/kegg-bin/show_pathway?mmu05205) | Proteoglycans in cancer | 3 of 199 | 0.76 | 0.0405 |
| [mmu04015](https://www.kegg.jp/kegg-bin/show_pathway?mmu04015) | Rap1 signaling pathway | 3 of 207 | 0.74 | 0.0440 |
| [mmu04010](https://www.kegg.jp/kegg-bin/show_pathway?mmu04010) | MAPK signaling pathway | 4 of 292 | 0.72 | 0.0249 |
| [mmu05200](https://www.kegg.jp/kegg-bin/show_pathway?mmu05200) | Pathways in cancer | 7 of 522 | 0.71 | 0.0037 |
| [mmu01100](https://www.kegg.jp/kegg-bin/show_pathway?mmu01100) | Metabolic pathways | 15 of 1296 | 0.64 | 6.82e-05 |

**INTESTINE**

**DOWN-EXPRESSED**

**CLUSTER 1**


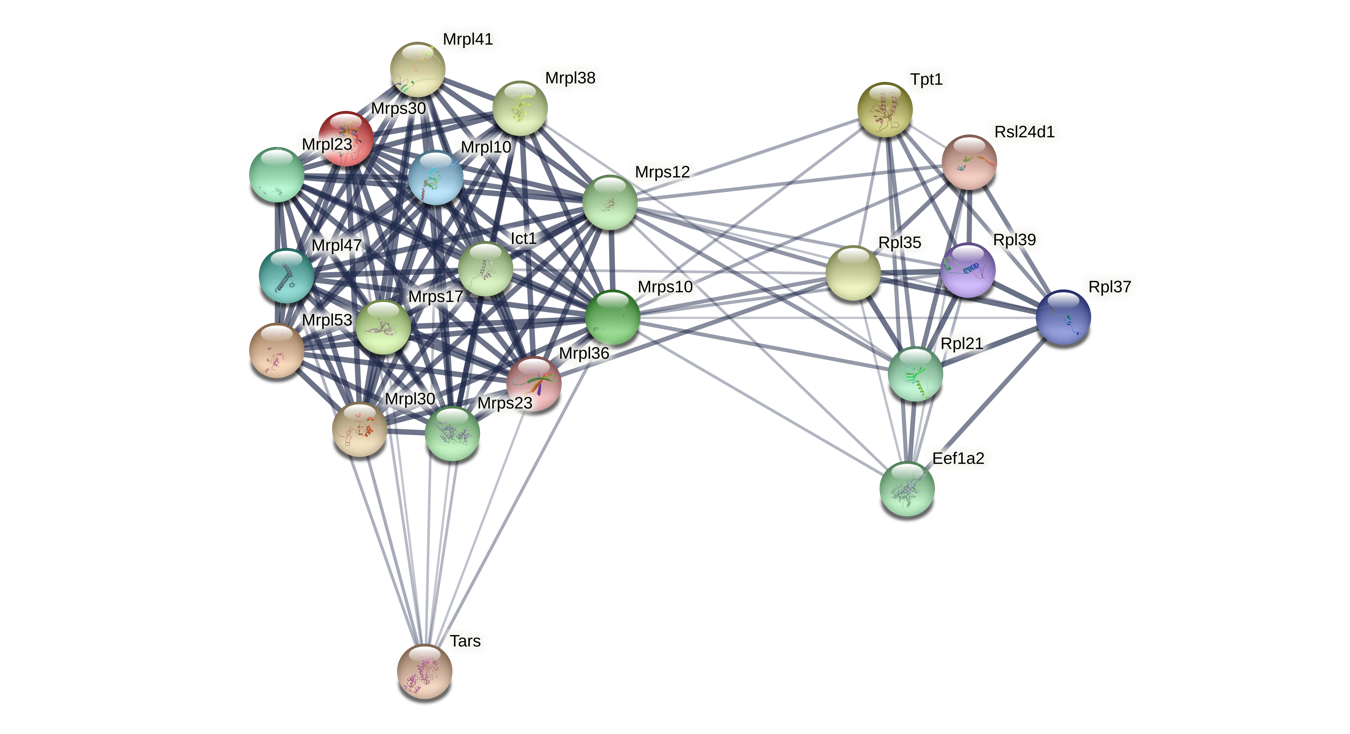


number of nodes: 22

number of edges: 138

average node degree: 12.5

avg. local clustering coefficient: 0.906

expected number of edges: 9

PPI enrichment p-value: < 1.0E^-16^

|  | **KEGG Pathways** |  |  |  |
| --- | --- | --- | --- | --- |
| ***pathway*** | ***description*** | ***count in network*** | ***strength*** | ***false discovery rate*** |
| [mmu03010](https://www.kegg.jp/kegg-bin/show_pathway?mmu03010) | Ribosome | 11 of 128 | 1.94 | 1.10e-18 |

**INTESTINE**

**DOWN-EXPRESSED**

**CLUSTER 2**

**
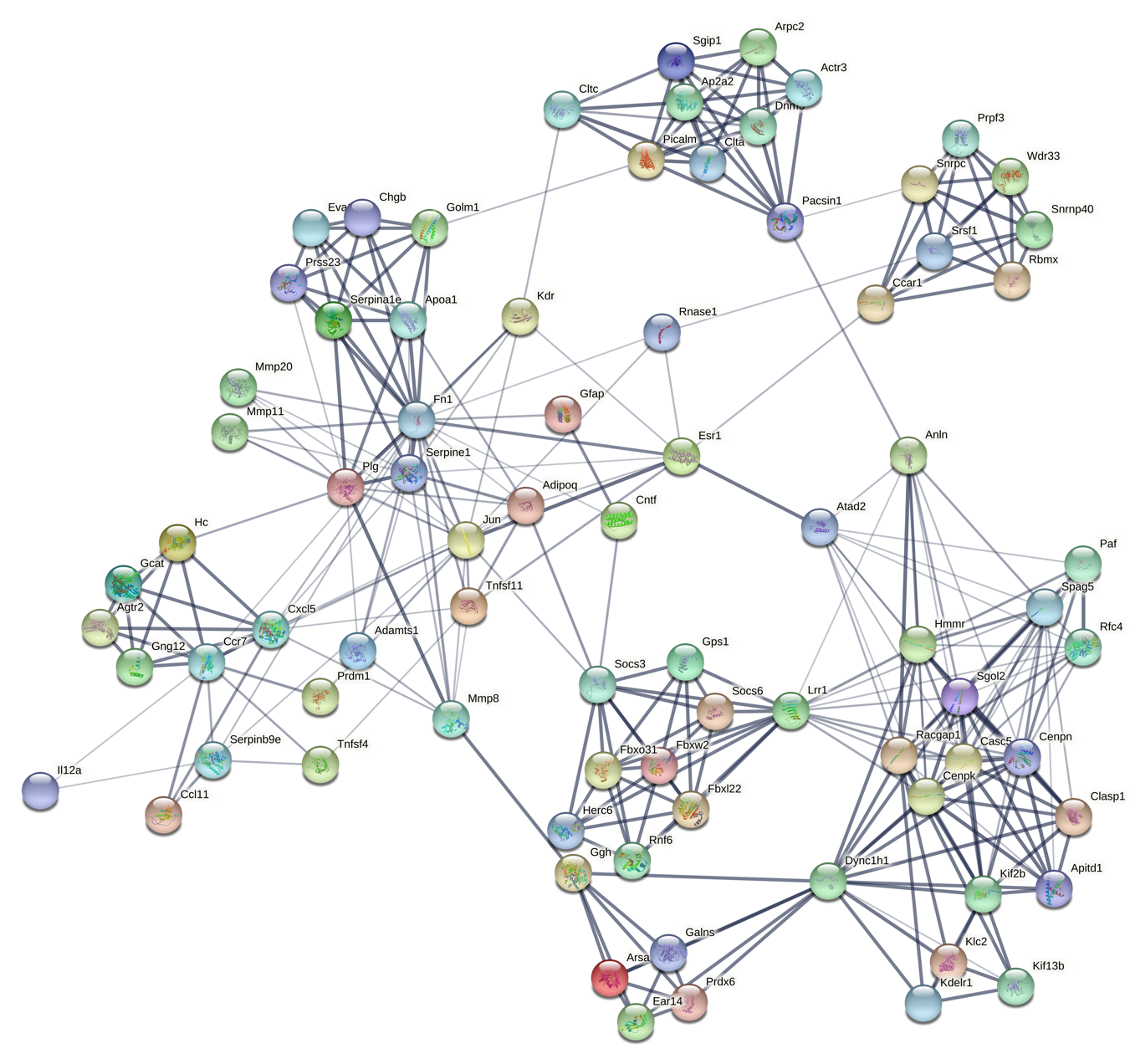
**

number of nodes: 80

number of edges: 324

average node degree: 8.1

avg. local clustering coefficient: 0.77

expected number of edges: 127

PPI enrichment p-value: < 1.0E^-16^

|  | **KEGG Pathways** |  |  |  |
| --- | --- | --- | --- | --- |
| ***pathway*** | ***description*** | ***count in network*** | ***strength*** | ***false discovery rate*** |
| [mmu04961](https://www.kegg.jp/kegg-bin/show_pathway?mmu04961) | Endocrine and other factor-regulated calcium reabsorption | 5 of 54 | 1.41 | 0.00030 |
| [mmu05100](https://www.kegg.jp/kegg-bin/show_pathway?mmu05100) | Bacterial invasion of epithelial cells | 5 of 74 | 1.27 | 0.00042 |
| [mmu04721](https://www.kegg.jp/kegg-bin/show_pathway?mmu04721) | Synaptic vesicle cycle | 4 of 62 | 1.25 | 0.0031 |
| [mmu05143](https://www.kegg.jp/kegg-bin/show_pathway?mmu05143) | African trypanosomiasis | 2 of 31 | 1.25 | 0.0441 |
| [mmu04917](https://www.kegg.jp/kegg-bin/show_pathway?mmu04917) | Prolactin signaling pathway | 4 of 72 | 1.19 | 0.0036 |

**INTESTINE**

**DOWN-EXPRESSED**

**CLUSTER 3**


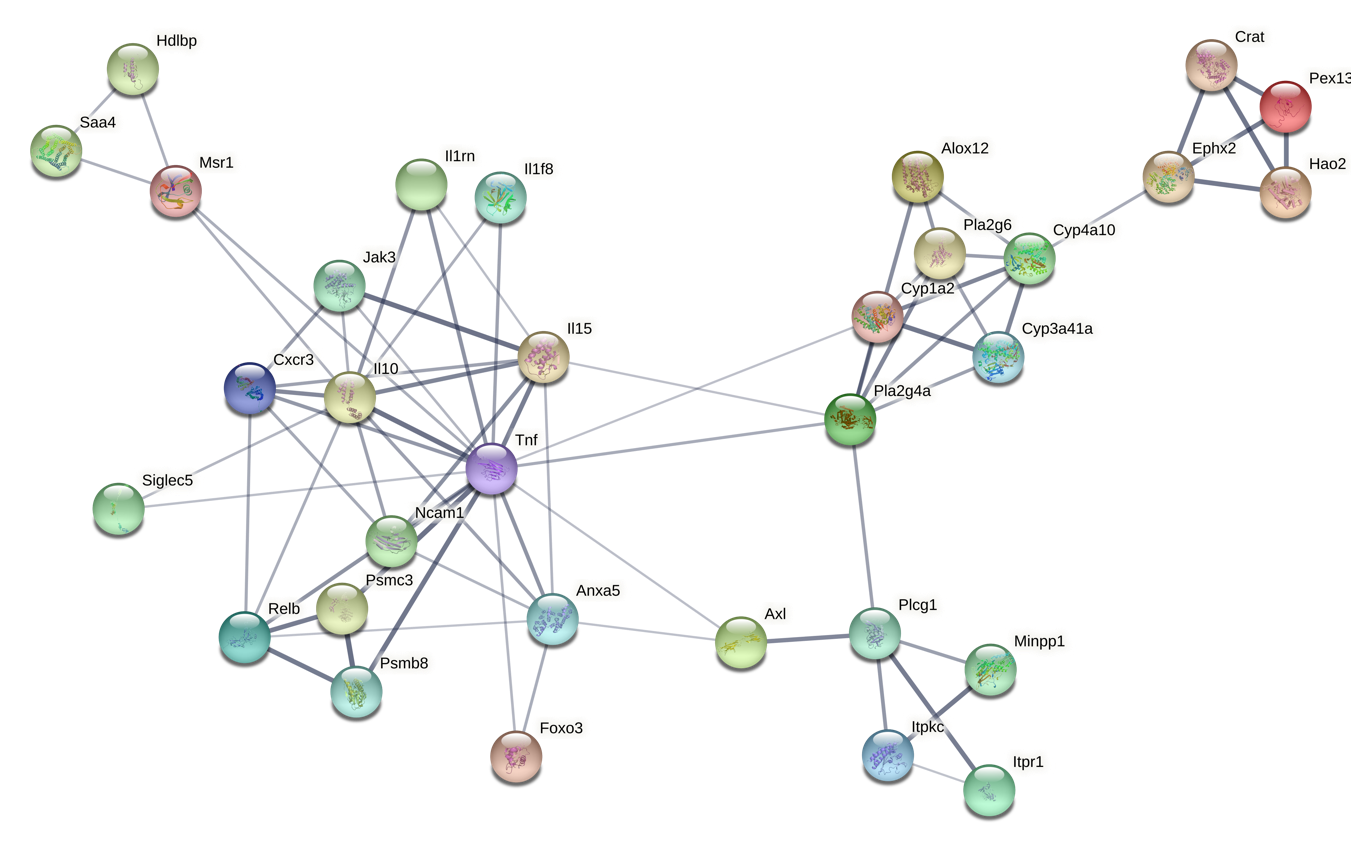


number of nodes: 32

number of edges: 73

average node degree: 4.56

avg. local clustering coefficient: 0.755

expected number of edges: 14

PPI enrichment p-value: < 1.0E^-16^

|  | **KEGG Pathways** |  |  |  |
| --- | --- | --- | --- | --- |
| ***pathway*** | ***description*** | ***count in network*** | ***strength*** | ***false discovery rate*** |
| [mmu05310](https://www.kegg.jp/kegg-bin/show_pathway?mmu05310) | Asthma | 2 of 23 | 1.78 | 0.0049 |
| [mmu00591](https://www.kegg.jp/kegg-bin/show_pathway?mmu00591) | Linoleic acid metabolism | 4 of 48 | 1.76 | 4.65E^-05^ |
| [mmu00592](https://www.kegg.jp/kegg-bin/show_pathway?mmu00592) | alpha-Linolenic acid metabolism | 2 of 24 | 1.76 | 0.0049 |
| [mmu01523](https://www.kegg.jp/kegg-bin/show_pathway?mmu01523) | Antifolate resistance | 2 of 30 | 1.66 | 0.0067 |
| [mmu05143](https://www.kegg.jp/kegg-bin/show_pathway?mmu05143) | African trypanosomiasis | 2 of 31 | 1.65 | 0.0068 |
| [mmu00590](https://www.kegg.jp/kegg-bin/show_pathway?mmu00590) | Arachidonic acid metabolism | 5 of 88 | 1.59 | 1.63E^-05^ |
| [mmu04750](https://www.kegg.jp/kegg-bin/show_pathway?mmu04750) | Inflammatory mediator regulation of TRP channels | 6 of 119 | 1.54 | 3.49E^-06^ |
| [mmu04672](https://www.kegg.jp/kegg-bin/show_pathway?mmu04672) | Intestinal immune network for IgA production | 2 of 40 | 1.54 | 0.0101 |
| [mmu04146](https://www.kegg.jp/kegg-bin/show_pathway?mmu04146) | Peroxisome | 4 of 84 | 1.52 | 0.00023 |
| [mmu05223](https://www.kegg.jp/kegg-bin/show_pathway?mmu05223) | Non-small cell lung cancer | 3 of 65 | 1.5 | 0.0024 |
| [mmu04664](https://www.kegg.jp/kegg-bin/show_pathway?mmu04664) | Fc epsilon RI signaling pathway | 3 of 66 | 1.5 | 0.0024 |
| [mmu05144](https://www.kegg.jp/kegg-bin/show_pathway?mmu05144) | Malaria | 2 of 45 | 1.49 | 0.0117 |
| [mmu03050](https://www.kegg.jp/kegg-bin/show_pathway?mmu03050) | Proteasome | 2 of 45 | 1.49 | 0.0117 |
| [mmu00565](https://www.kegg.jp/kegg-bin/show_pathway?mmu00565) | Ether lipid metabolism | 2 of 45 | 1.49 | 0.0117 |
| [mmu00562](https://www.kegg.jp/kegg-bin/show_pathway?mmu00562) | Inositol phosphate metabolism | 3 of 72 | 1.46 | 0.0026 |
| [mmu01521](https://www.kegg.jp/kegg-bin/show_pathway?mmu01521) | EGFR tyrosine kinase inhibitor resistance | 3 of 79 | 1.42 | 0.0031 |
| [mmu05330](https://www.kegg.jp/kegg-bin/show_pathway?mmu05330) | Allograft rejection | 2 of 52 | 1.42 | 0.0129 |
| [mmu04666](https://www.kegg.jp/kegg-bin/show_pathway?mmu04666) | Fc gamma R-mediated phagocytosis | 3 of 83 | 1.4 | 0.0032 |
| [mmu05321](https://www.kegg.jp/kegg-bin/show_pathway?mmu05321) | Inflammatory bowel disease (IBD) | 2 of 58 | 1.38 | 0.0150 |
| [mmu04370](https://www.kegg.jp/kegg-bin/show_pathway?mmu04370) | VEGF signaling pathway | 2 of 58 | 1.38 | 0.0150 |
| [mmu00830](https://www.kegg.jp/kegg-bin/show_pathway?mmu00830) | Retinol metabolism | 3 of 89 | 1.37 | 0.0036 |
| [mmu04730](https://www.kegg.jp/kegg-bin/show_pathway?mmu04730) | Long-term depression | 2 of 60 | 1.36 | 0.0151 |
| [mmu04064](https://www.kegg.jp/kegg-bin/show_pathway?mmu04064) | NF-kappa B signaling pathway | 3 of 93 | 1.35 | 0.0038 |
| [mmu04270](https://www.kegg.jp/kegg-bin/show_pathway?mmu04270) | Vascular smooth muscle contraction | 4 of 125 | 1.34 | 0.00075 |
| [mmu04070](https://www.kegg.jp/kegg-bin/show_pathway?mmu04070) | Phosphatidylinositol signaling system | 3 of 96 | 1.33 | 0.0039 |
| [mmu05140](https://www.kegg.jp/kegg-bin/show_pathway?mmu05140) | Leishmaniasis | 2 of 65 | 1.33 | 0.0171 |
| [mmu04660](https://www.kegg.jp/kegg-bin/show_pathway?mmu04660) | T cell receptor signaling pathway | 3 of 100 | 1.32 | 0.0041 |
| [mmu05133](https://www.kegg.jp/kegg-bin/show_pathway?mmu05133) | Pertussis | 2 of 74 | 1.27 | 0.0207 |
| [mmu05169](https://www.kegg.jp/kegg-bin/show_pathway?mmu05169) | Epstein-Barr virus infection | 5 of 205 | 1.23 | 0.00030 |
| [mmu05323](https://www.kegg.jp/kegg-bin/show_pathway?mmu05323) | Rheumatoid arthritis | 2 of 81 | 1.23 | 0.0240 |
| [mmu00140](https://www.kegg.jp/kegg-bin/show_pathway?mmu00140) | Steroid hormone biosynthesis | 2 of 83 | 1.22 | 0.0245 |
| [mmu04726](https://www.kegg.jp/kegg-bin/show_pathway?mmu04726) | Serotonergic synapse | 3 of 130 | 1.2 | 0.0066 |
| [mmu04912](https://www.kegg.jp/kegg-bin/show_pathway?mmu04912) | GnRH signaling pathway | 2 of 87 | 1.2 | 0.0256 |
| [mmu04658](https://www.kegg.jp/kegg-bin/show_pathway?mmu04658) | Th1 and Th2 cell differentiation | 2 of 86 | 1.2 | 0.0256 |
| [mmu05322](https://www.kegg.jp/kegg-bin/show_pathway?mmu05322) | Systemic lupus erythematosus | 2 of 92 | 1.18 | 0.0277 |
| [mmu05204](https://www.kegg.jp/kegg-bin/show_pathway?mmu05204) | Chemical carcinogenesis | 2 of 92 | 1.18 | 0.0277 |
| [mmu00564](https://www.kegg.jp/kegg-bin/show_pathway?mmu00564) | Glycerophospholipid metabolism | 2 of 95 | 1.16 | 0.0282 |
| [mmu05231](https://www.kegg.jp/kegg-bin/show_pathway?mmu05231) | Choline metabolism in cancer | 2 of 98 | 1.15 | 0.0286 |
| [mmu04933](https://www.kegg.jp/kegg-bin/show_pathway?mmu04933) | AGE-RAGE signaling pathway in diabetic complications | 2 of 100 | 1.14 | 0.0291 |
| [mmu04659](https://www.kegg.jp/kegg-bin/show_pathway?mmu04659) | Th17 cell differentiation | 2 of 100 | 1.14 | 0.0291 |
| [mmu05142](https://www.kegg.jp/kegg-bin/show_pathway?mmu05142) | Chagas disease (American trypanosomiasis) | 2 of 101 | 1.13 | 0.0291 |
| [mmu04217](https://www.kegg.jp/kegg-bin/show_pathway?mmu04217) | Necroptosis | 3 of 158 | 1.12 | 0.0100 |
| [mmu05146](https://www.kegg.jp/kegg-bin/show_pathway?mmu05146) | Amoebiasis | 2 of 105 | 1.12 | 0.0301 |
| [mmu04630](https://www.kegg.jp/kegg-bin/show_pathway?mmu04630) | Jak-STAT signaling pathway | 3 of 161 | 1.11 | 0.0101 |
| [mmu05145](https://www.kegg.jp/kegg-bin/show_pathway?mmu05145) | Toxoplasmosis | 2 of 107 | 1.11 | 0.0306 |
| [mmu04668](https://www.kegg.jp/kegg-bin/show_pathway?mmu04668) | TNF signaling pathway | 2 of 108 | 1.11 | 0.0306 |
| [mmu04650](https://www.kegg.jp/kegg-bin/show_pathway?mmu04650) | Natural killer cell mediated cytotoxicity | 2 of 111 | 1.09 | 0.0316 |
| [mmu04724](https://www.kegg.jp/kegg-bin/show_pathway?mmu04724) | Glutamatergic synapse | 2 of 113 | 1.09 | 0.0321 |
| [mmu04062](https://www.kegg.jp/kegg-bin/show_pathway?mmu04062) | Chemokine signaling pathway | 3 of 179 | 1.06 | 0.0117 |
| [mmu04020](https://www.kegg.jp/kegg-bin/show_pathway?mmu04020) | Calcium signaling pathway | 3 of 180 | 1.06 | 0.0117 |
| [mmu04722](https://www.kegg.jp/kegg-bin/show_pathway?mmu04722) | Neurotrophin signaling pathway | 2 of 119 | 1.06 | 0.0347 |
| [mmu04611](https://www.kegg.jp/kegg-bin/show_pathway?mmu04611) | Platelet activation | 2 of 122 | 1.05 | 0.0357 |
| [mmu04380](https://www.kegg.jp/kegg-bin/show_pathway?mmu04380) | Osteoclast differentiation | 2 of 122 | 1.05 | 0.0357 |
| [mmu04060](https://www.kegg.jp/kegg-bin/show_pathway?mmu04060) | Cytokine-cytokine receptor interaction | 4 of 252 | 1.04 | 0.0043 |
| [mmu05205](https://www.kegg.jp/kegg-bin/show_pathway?mmu05205) | Proteoglycans in cancer | 3 of 199 | 1.02 | 0.0139 |
| [mmu04068](https://www.kegg.jp/kegg-bin/show_pathway?mmu04068) | FoxO signaling pathway | 2 of 132 | 1.02 | 0.0400 |
| [mmu05166](https://www.kegg.jp/kegg-bin/show_pathway?mmu05166) | HTLV-I infection | 4 of 269 | 1.01 | 0.0049 |
| [mmu04210](https://www.kegg.jp/kegg-bin/show_pathway?mmu04210) | Apoptosis | 2 of 135 | 1.01 | 0.0410 |
| [mmu04072](https://www.kegg.jp/kegg-bin/show_pathway?mmu04072) | Phospholipase D signaling pathway | 2 of 145 | 0.98 | 0.0461 |
| [mmu04921](https://www.kegg.jp/kegg-bin/show_pathway?mmu04921) | Oxytocin signaling pathway | 2 of 149 | 0.97 | 0.0477 |
| [mmu04014](https://www.kegg.jp/kegg-bin/show_pathway?mmu04014) | Ras signaling pathway | 3 of 228 | 0.96 | 0.0176 |
| [mmu04010](https://www.kegg.jp/kegg-bin/show_pathway?mmu04010) | MAPK signaling pathway | 3 of 292 | 0.85 | 0.0282 |
| [mmu01100](https://www.kegg.jp/kegg-bin/show_pathway?mmu01100) | Metabolic pathways | 11 of 1296 | 0.77 | 4.65E^-05^ |

**SPINE**

**UP-EXPRESSED**

**CLUSTER 1**


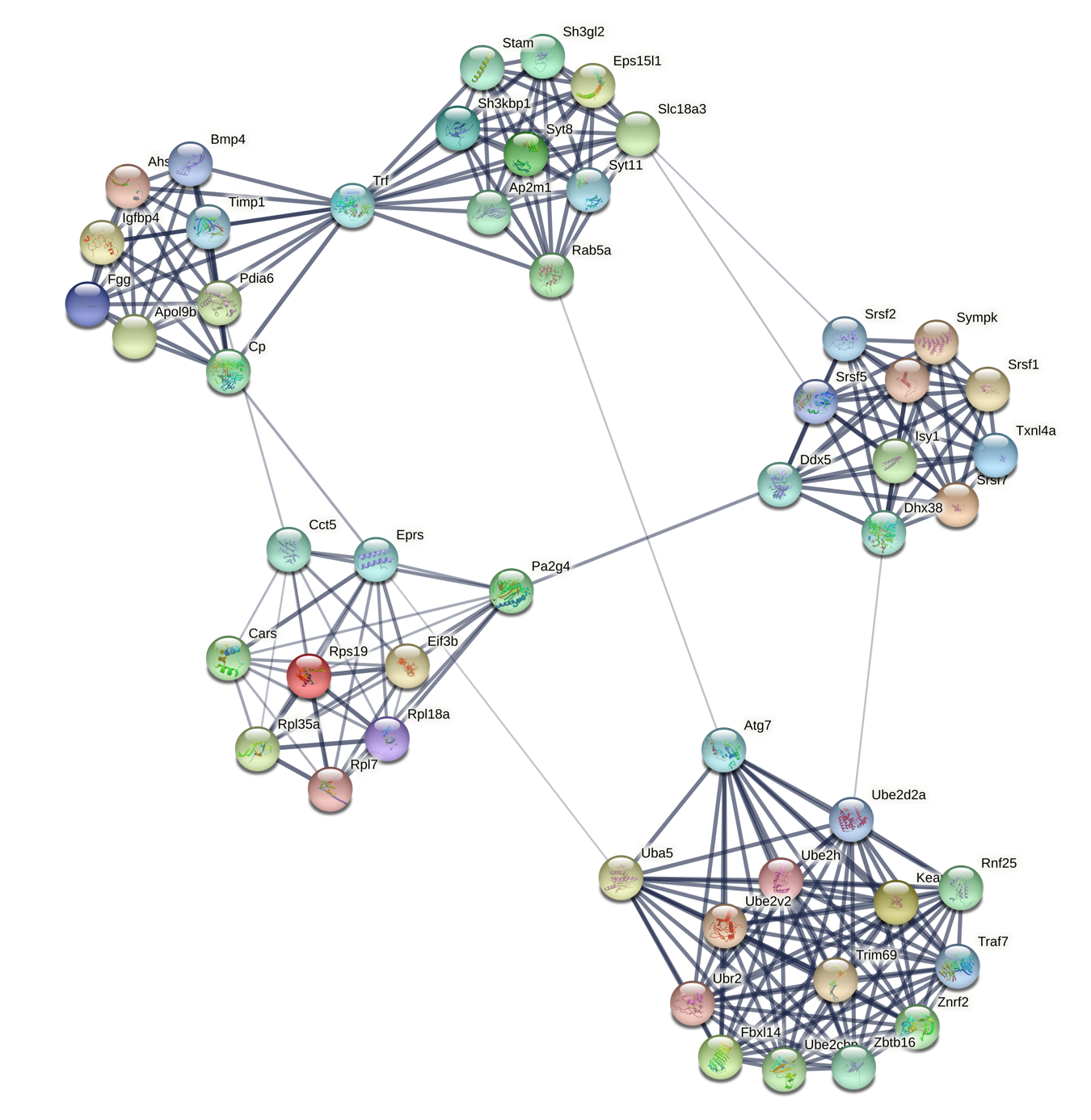


number of nodes: 51

number of edges: 260

average node degree: 10.2

avg. local clustering coefficient: 0.927

expected number of edges: 76

PPI enrichment p-value: < 1.0E-^16^

|  | **KEGG Pathways** |  |  |  |
| --- | --- | --- | --- | --- |
| ***pathway*** | ***description*** | ***count in network*** | ***strength*** | ***false discovery rate*** |
| [mmu04216](https://www.kegg.jp/kegg-bin/show_pathway?mmu04216) | Ferroptosis | 3 of 40 | 1.51 | 0.0019 |
| [mmu03040](https://www.kegg.jp/kegg-bin/show_pathway?mmu03040) | Spliceosome | 8 of 130 | 1.42 | 4.12e-08 |
| [mmu00860](https://www.kegg.jp/kegg-bin/show_pathway?mmu00860) | Porphyrin and chlorophyll metabolism | 2 of 39 | 1.35 | 0.0290 |
| [mmu00970](https://www.kegg.jp/kegg-bin/show_pathway?mmu00970) | Aminoacyl-tRNA biosynthesis | 2 of 44 | 1.29 | 0.0290 |
| [mmu04721](https://www.kegg.jp/kegg-bin/show_pathway?mmu04721) | Synaptic vesicle cycle | 2 of 62 | 1.14 | 0.0459 |
| [mmu03010](https://www.kegg.jp/kegg-bin/show_pathway?mmu03010) | Ribosome | 4 of 128 | 1.13 | 0.0026 |
| [mmu04144](https://www.kegg.jp/kegg-bin/show_pathway?mmu04144) | Endocytosis | 6 of 258 | 1.0 | 0.00068 |
| [mmu04120](https://www.kegg.jp/kegg-bin/show_pathway?mmu04120) | Ubiquitin mediated proteolysis | 3 of 137 | 0.98 | 0.0290 |
| [mmu05168](https://www.kegg.jp/kegg-bin/show_pathway?mmu05168) | Herpes simplex infection | 4 of 202 | 0.93 | 0.0112 |

**SPINE**

**UP-EXPRESSED**

**CLUSTER 2**


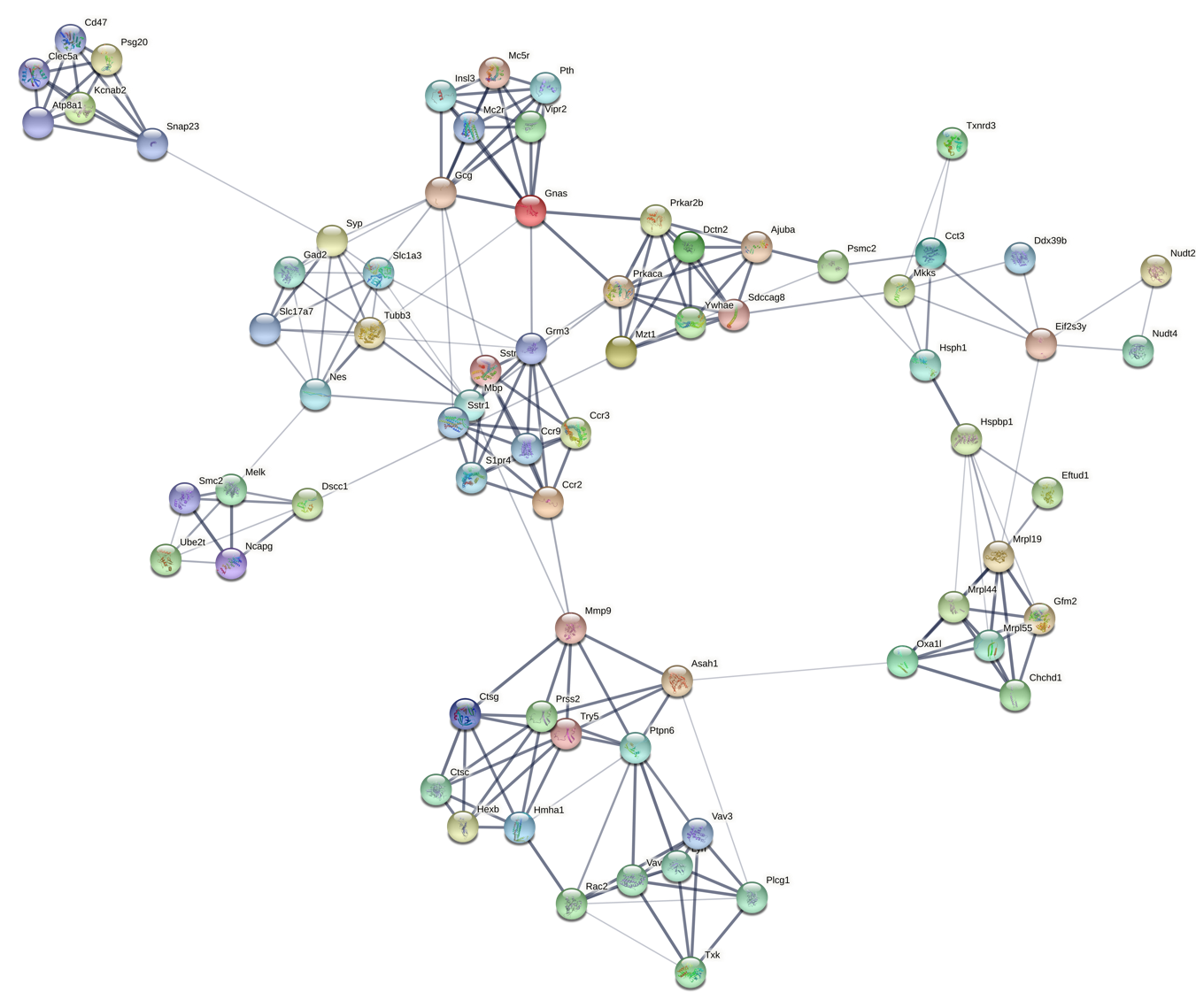


number of nodes: 71

number of edges: 213

average node degree: 6

avg. local clustering coefficient: 0.749

expected number of edges: 92

PPI enrichment p-value: < 1.0E^-16^

|  | **KEGG Pathways** |  |  |  |
| --- | --- | --- | --- | --- |
| ***pathway*** | ***description*** | ***count in network*** | ***strength*** | ***false discovery rate*** |
| [mmu04664](https://www.kegg.jp/kegg-bin/show_pathway?mmu04664) | Fc epsilon RI signaling pathway | 5 of 66 | 1.37 | 0.00010 |
| [mmu04662](https://www.kegg.jp/kegg-bin/show_pathway?mmu04662) | B cell receptor signaling pathway | 5 of 69 | 1.35 | 0.00010 |
| [mmu04962](https://www.kegg.jp/kegg-bin/show_pathway?mmu04962) | Vasopressin-regulated water reabsorption | 3 of 43 | 1.34 | 0.0061 |
| [mmu05030](https://www.kegg.jp/kegg-bin/show_pathway?mmu05030) | Cocaine addiction | 3 of 48 | 1.29 | 0.0074 |
| [mmu04666](https://www.kegg.jp/kegg-bin/show_pathway?mmu04666) | Fc gamma R-mediated phagocytosis | 5 of 83 | 1.27 | 0.00021 |
| [mmu04670](https://www.kegg.jp/kegg-bin/show_pathway?mmu04670) | Leukocyte transendothelial migration | 6 of 115 | 1.21 | 9.82e-05 |
| [mmu04024](https://www.kegg.jp/kegg-bin/show_pathway?mmu04024) | cAMP signaling pathway | 9 of 194 | 1.16 | 2.73e-06 |
| [mmu04650](https://www.kegg.jp/kegg-bin/show_pathway?mmu04650) | Natural killer cell mediated cytotoxicity | 5 of 111 | 1.15 | 0.00070 |
| [mmu04927](https://www.kegg.jp/kegg-bin/show_pathway?mmu04927) | Cortisol synthesis and secretion | 3 of 66 | 1.15 | 0.0130 |
| [mmu04062](https://www.kegg.jp/kegg-bin/show_pathway?mmu04062) | Chemokine signaling pathway | 8 of 179 | 1.14 | 7.84e-06 |
| [mmu04724](https://www.kegg.jp/kegg-bin/show_pathway?mmu04724) | Glutamatergic synapse | 5 of 113 | 1.14 | 0.00070 |
| [mmu04660](https://www.kegg.jp/kegg-bin/show_pathway?mmu04660) | T cell receptor signaling pathway | 4 of 100 | 1.09 | 0.0055 |
| [mmu04080](https://www.kegg.jp/kegg-bin/show_pathway?mmu04080) | Neuroactive ligand-receptor interaction | 10 of 284 | 1.04 | 2.73e-06 |
| [mmu04911](https://www.kegg.jp/kegg-bin/show_pathway?mmu04911) | Insulin secretion | 3 of 85 | 1.04 | 0.0246 |
| [mmu04540](https://www.kegg.jp/kegg-bin/show_pathway?mmu04540) | Gap junction | 3 of 85 | 1.04 | 0.0246 |
| [mmu04611](https://www.kegg.jp/kegg-bin/show_pathway?mmu04611) | Platelet activation | 4 of 122 | 1.01 | 0.0080 |
| [mmu01522](https://www.kegg.jp/kegg-bin/show_pathway?mmu01522) | Endocrine resistance | 3 of 92 | 1.01 | 0.0275 |
| [mmu04142](https://www.kegg.jp/kegg-bin/show_pathway?mmu04142) | Lysosome | 4 of 123 | 1.0 | 0.0080 |
| [mmu04926](https://www.kegg.jp/kegg-bin/show_pathway?mmu04926) | Relaxin signaling pathway | 4 of 130 | 0.98 | 0.0089 |
| [mmu04925](https://www.kegg.jp/kegg-bin/show_pathway?mmu04925) | Aldosterone synthesis and secretion | 3 of 97 | 0.98 | 0.0303 |
| [mmu04922](https://www.kegg.jp/kegg-bin/show_pathway?mmu04922) | Glucagon signaling pathway | 3 of 99 | 0.97 | 0.0303 |
| [mmu04972](https://www.kegg.jp/kegg-bin/show_pathway?mmu04972) | Pancreatic secretion | 3 of 101 | 0.96 | 0.0303 |
| [mmu05146](https://www.kegg.jp/kegg-bin/show_pathway?mmu05146) | Amoebiasis | 3 of 105 | 0.95 | 0.0303 |
| [mmu04750](https://www.kegg.jp/kegg-bin/show_pathway?mmu04750) | Inflammatory mediator regulation of TRP channels | 3 of 119 | 0.89 | 0.0399 |
| [mmu04071](https://www.kegg.jp/kegg-bin/show_pathway?mmu04071) | Sphingolipid signaling pathway | 3 of 120 | 0.89 | 0.0399 |
| [mmu05169](https://www.kegg.jp/kegg-bin/show_pathway?mmu05169) | Epstein-Barr virus infection | 5 of 205 | 0.88 | 0.0074 |
| [mmu04915](https://www.kegg.jp/kegg-bin/show_pathway?mmu04915) | Estrogen signaling pathway | 3 of 131 | 0.85 | 0.0476 |
| [mmu05205](https://www.kegg.jp/kegg-bin/show_pathway?mmu05205) | Proteoglycans in cancer | 4 of 199 | 0.8 | 0.0303 |
| [mmu05203](https://www.kegg.jp/kegg-bin/show_pathway?mmu05203) | Viral carcinogenesis | 4 of 199 | 0.8 | 0.0303 |
| [mmu05200](https://www.kegg.jp/kegg-bin/show_pathway?mmu05200) | Pathways in cancer | 6 of 522 | 0.55 | 0.0399 |

**SPINE**

**UP-EXPRESSED**

**CLUSTER 3**

**
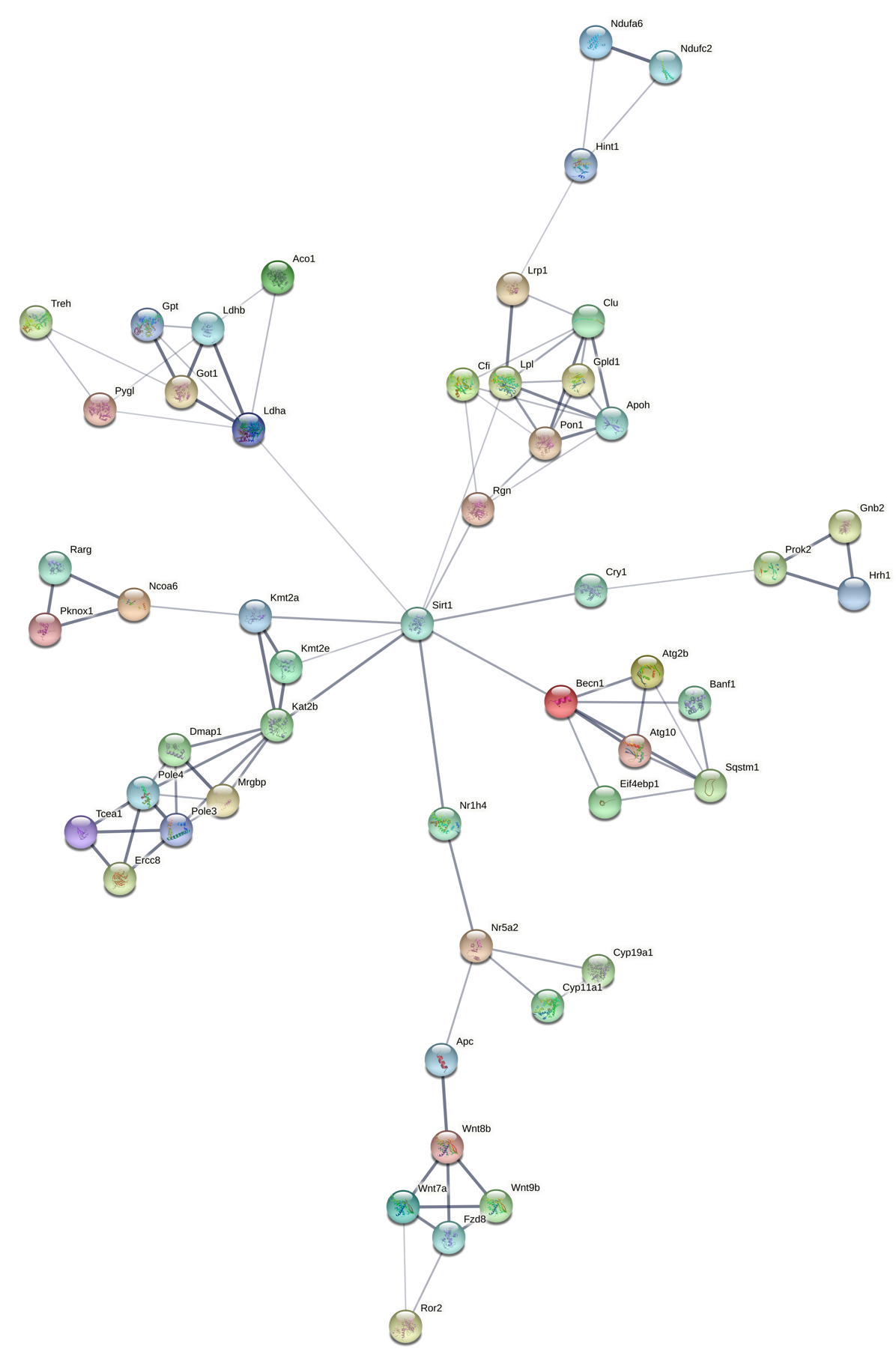
**

number of nodes: 51

number of edges: 93

average node degree: 3.65

avg. local clustering coefficient: 0.662

expected number of edges: 21

PPI enrichment p-value: < 1.0E^-16^

|  | **KEGG Pathways** |  |  |  |
| --- | --- | --- | --- | --- |
| ***pathway*** | ***description*** | ***count in network*** | ***strength*** | ***false discovery rate*** |
| [mmu01210](https://www.kegg.jp/kegg-bin/show_pathway?mmu01210) | 2-Oxocarboxylic acid metabolism | 3 of 19 | 1.83 | 0.00038 |
| [mmu00220](https://www.kegg.jp/kegg-bin/show_pathway?mmu00220) | Arginine biosynthesis | 2 of 19 | 1.66 | 0.0051 |
| [mmu04136](https://www.kegg.jp/kegg-bin/show_pathway?mmu04136) | Autophagy - other | 3 of 32 | 1.61 | 0.00060 |
| [mmu05217](https://www.kegg.jp/kegg-bin/show_pathway?mmu05217) | Basal cell carcinoma | 5 of 63 | 1.54 | 1.82e-05 |
| [mmu03420](https://www.kegg.jp/kegg-bin/show_pathway?mmu03420) | Nucleotide excision repair | 3 of 43 | 1.48 | 0.0011 |
| [mmu00500](https://www.kegg.jp/kegg-bin/show_pathway?mmu00500) | Starch and sucrose metabolism | 2 of 30 | 1.46 | 0.0104 |
| [mmu00270](https://www.kegg.jp/kegg-bin/show_pathway?mmu00270) | Cysteine and methionine metabolism | 3 of 46 | 1.45 | 0.0011 |
| [mmu00640](https://www.kegg.jp/kegg-bin/show_pathway?mmu00640) | Propanoate metabolism | 2 of 31 | 1.45 | 0.0106 |
| [mmu04979](https://www.kegg.jp/kegg-bin/show_pathway?mmu04979) | Cholesterol metabolism | 3 of 48 | 1.43 | 0.0012 |
| [mmu03410](https://www.kegg.jp/kegg-bin/show_pathway?mmu03410) | Base excision repair | 2 of 33 | 1.42 | 0.0115 |
| [mmu03030](https://www.kegg.jp/kegg-bin/show_pathway?mmu03030) | DNA replication | 2 of 35 | 1.39 | 0.0124 |
| [mmu00250](https://www.kegg.jp/kegg-bin/show_pathway?mmu00250) | Alanine, aspartate and glutamate metabolism | 2 of 36 | 1.38 | 0.0126 |
| [mmu00620](https://www.kegg.jp/kegg-bin/show_pathway?mmu00620) | Pyruvate metabolism | 2 of 38 | 1.36 | 0.0131 |
| [mmu04934](https://www.kegg.jp/kegg-bin/show_pathway?mmu04934) | Cushing's syndrome | 7 of 156 | 1.29 | 1.00e-05 |
| [mmu04916](https://www.kegg.jp/kegg-bin/show_pathway?mmu04916) | Melanogenesis | 4 of 98 | 1.25 | 0.00070 |
| [mmu04922](https://www.kegg.jp/kegg-bin/show_pathway?mmu04922) | Glucagon signaling pathway | 4 of 99 | 1.24 | 0.00070 |
| [mmu01230](https://www.kegg.jp/kegg-bin/show_pathway?mmu01230) | Biosynthesis of amino acids | 3 of 75 | 1.24 | 0.0039 |
| [mmu04550](https://www.kegg.jp/kegg-bin/show_pathway?mmu04550) | Signaling pathways regulating pluripotency of stem cells | 5 of 137 | 1.2 | 0.00038 |
| [mmu04913](https://www.kegg.jp/kegg-bin/show_pathway?mmu04913) | Ovarian steroidogenesis | 2 of 57 | 1.18 | 0.0256 |
| [mmu05224](https://www.kegg.jp/kegg-bin/show_pathway?mmu05224) | Breast cancer | 5 of 146 | 1.17 | 0.00040 |
| [mmu04310](https://www.kegg.jp/kegg-bin/show_pathway?mmu04310) | Wnt signaling pathway | 5 of 146 | 1.17 | 0.00040 |
| [mmu01200](https://www.kegg.jp/kegg-bin/show_pathway?mmu01200) | Carbon metabolism | 4 of 118 | 1.17 | 0.0011 |
| [mmu00310](https://www.kegg.jp/kegg-bin/show_pathway?mmu00310) | Lysine degradation | 2 of 58 | 1.17 | 0.0257 |
| [mmu05226](https://www.kegg.jp/kegg-bin/show_pathway?mmu05226) | Gastric cancer | 5 of 148 | 1.16 | 0.00040 |
| [mmu04390](https://www.kegg.jp/kegg-bin/show_pathway?mmu04390) | Hippo signaling pathway | 5 of 153 | 1.15 | 0.00040 |
| [mmu04150](https://www.kegg.jp/kegg-bin/show_pathway?mmu04150) | mTOR signaling pathway | 5 of 152 | 1.15 | 0.00040 |
| [mmu04137](https://www.kegg.jp/kegg-bin/show_pathway?mmu04137) | Mitophagy - animal | 2 of 63 | 1.14 | 0.0292 |
| [mmu00010](https://www.kegg.jp/kegg-bin/show_pathway?mmu00010) | Glycolysis / Gluconeogenesis | 2 of 65 | 1.12 | 0.0301 |
| [mmu05166](https://www.kegg.jp/kegg-bin/show_pathway?mmu05166) | HTLV-I infection | 8 of 269 | 1.11 | 1.20e-05 |
| [mmu05225](https://www.kegg.jp/kegg-bin/show_pathway?mmu05225) | Hepatocellular carcinoma | 5 of 168 | 1.11 | 0.00044 |
| [mmu05010](https://www.kegg.jp/kegg-bin/show_pathway?mmu05010) | Alzheimer's disease | 4 of 167 | 1.02 | 0.0034 |
| [mmu00140](https://www.kegg.jp/kegg-bin/show_pathway?mmu00140) | Steroid hormone biosynthesis | 2 of 83 | 1.02 | 0.0461 |
| [mmu04140](https://www.kegg.jp/kegg-bin/show_pathway?mmu04140) | Autophagy - animal | 3 of 129 | 1.0 | 0.0126 |
| [mmu04723](https://www.kegg.jp/kegg-bin/show_pathway?mmu04723) | Retrograde endocannabinoid signaling | 3 of 145 | 0.95 | 0.0160 |
| [mmu05205](https://www.kegg.jp/kegg-bin/show_pathway?mmu05205) | Proteoglycans in cancer | 4 of 199 | 0.94 | 0.0056 |
| [mmu05165](https://www.kegg.jp/kegg-bin/show_pathway?mmu05165) | Human papillomavirus infection | 6 of 335 | 0.89 | 0.00088 |
| [mmu04218](https://www.kegg.jp/kegg-bin/show_pathway?mmu04218) | Cellular senescence | 3 of 171 | 0.88 | 0.0243 |
| [mmu05200](https://www.kegg.jp/kegg-bin/show_pathway?mmu05200) | Pathways in cancer | 6 of 522 | 0.7 | 0.0057 |
| [mmu01100](https://www.kegg.jp/kegg-bin/show_pathway?mmu01100) | Metabolic pathways | 14 of 1296 | 0.67 | 2.74e-05 |

**SPINE**

**DOWN-EXPRESSED**

**CLUSTER 1**


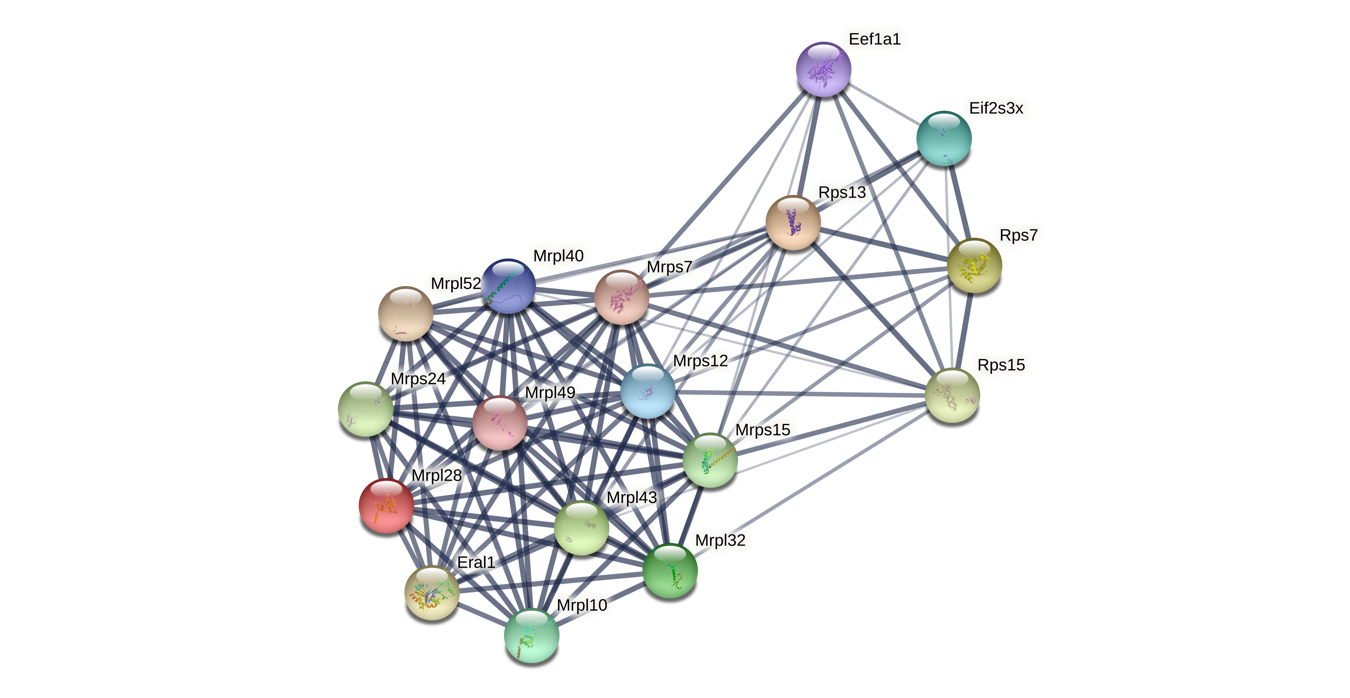
number of nodes: 17

number of edges: 100

average node degree: 11.8

avg. local clustering coefficient: 0.875

expected number of edges: 7

PPI enrichment p-value: < 1.0E^-16^

|  | **KEGG Pathways** |  |  |  |
| --- | --- | --- | --- | --- |
| ***pathway*** | ***description*** | ***count in network*** | ***strength*** | ***false discovery rate*** |
| [mmu03010](https://www.kegg.jp/kegg-bin/show_pathway?mmu03010) | Ribosome | 9 of 128 | 1.96 | 7.35e-16 |
| [mmu03013](https://www.kegg.jp/kegg-bin/show_pathway?mmu03013) | RNA transport | 2 of 164 | 1.2 | 0.0107 |

**SPINE**

**DOWN-EXPRESSED**

**CLUSTER 2**


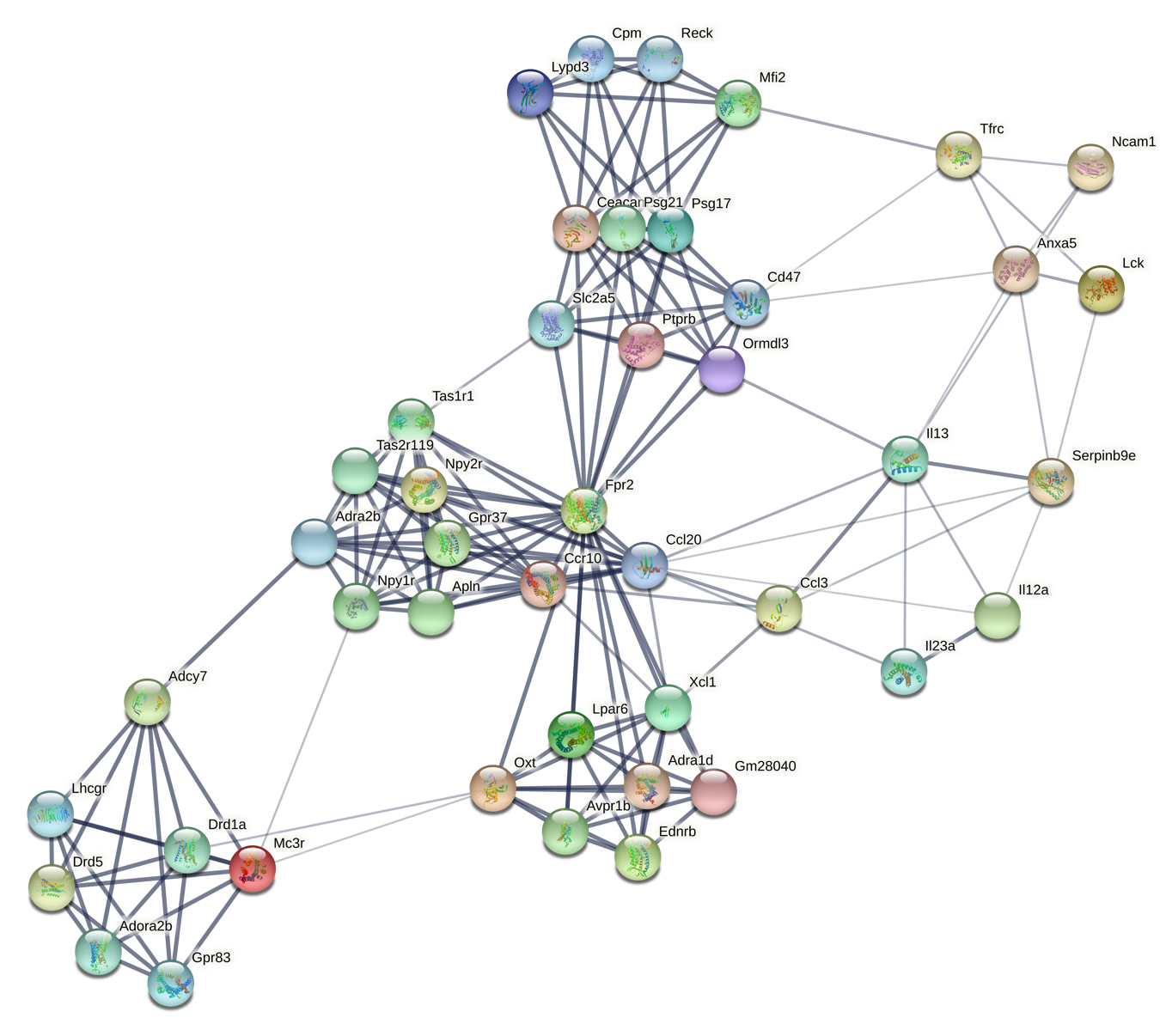


number of nodes: 44

number of edges: 174

average node degree: 7.91

avg. local clustering coefficient: 0.777

expected number of edges: 28

PPI enrichment p-value: < 1.0E^-16^

|  | **KEGG Pathways** |  |  |  |
| --- | --- | --- | --- | --- |
| ***pathway*** | ***description*** | ***count in network*** | ***strength*** | ***false discovery rate*** |
| [mmu05321](https://www.kegg.jp/kegg-bin/show_pathway?mmu05321) | Inflammatory bowel disease (IBD) | 3 of 58 | 1.41 | 0.0040 |
| [mmu04080](https://www.kegg.jp/kegg-bin/show_pathway?mmu04080) | Neuroactive ligand-receptor interaction | 14 of 284 | 1.39 | 3.81e-14 |
| [mmu04020](https://www.kegg.jp/kegg-bin/show_pathway?mmu04020) | Calcium signaling pathway | 8 of 180 | 1.35 | 1.58e-07 |
| [mmu05323](https://www.kegg.jp/kegg-bin/show_pathway?mmu05323) | Rheumatoid arthritis | 3 of 81 | 1.27 | 0.0072 |
| [mmu04923](https://www.kegg.jp/kegg-bin/show_pathway?mmu04923) | Regulation of lipolysis in adipocytes | 2 of 55 | 1.26 | 0.0404 |
| [mmu04913](https://www.kegg.jp/kegg-bin/show_pathway?mmu04913) | Ovarian steroidogenesis | 2 of 57 | 1.25 | 0.0404 |
| [mmu04658](https://www.kegg.jp/kegg-bin/show_pathway?mmu04658) | Th1 and Th2 cell differentiation | 3 of 86 | 1.24 | 0.0072 |
| [mmu04270](https://www.kegg.jp/kegg-bin/show_pathway?mmu04270) | Vascular smooth muscle contraction | 4 of 125 | 1.21 | 0.0025 |
| [mmu04062](https://www.kegg.jp/kegg-bin/show_pathway?mmu04062) | Chemokine signaling pathway | 5 of 179 | 1.15 | 0.00078 |
| [mmu04060](https://www.kegg.jp/kegg-bin/show_pathway?mmu04060) | Cytokine-cytokine receptor interaction | 7 of 252 | 1.14 | 2.43e-05 |
| [mmu04022](https://www.kegg.jp/kegg-bin/show_pathway?mmu04022) | cGMP-PKG signaling pathway | 4 of 164 | 1.09 | 0.0048 |
| [mmu04072](https://www.kegg.jp/kegg-bin/show_pathway?mmu04072) | Phospholipase D signaling pathway | 3 of 145 | 1.02 | 0.0261 |
| [mmu04024](https://www.kegg.jp/kegg-bin/show_pathway?mmu04024) | cAMP signaling pathway | 4 of 194 | 1.01 | 0.0072 |
| [mmu04630](https://www.kegg.jp/kegg-bin/show_pathway?mmu04630) | Jak-STAT signaling pathway | 3 of 161 | 0.97 | 0.0321 |
| [mmu05200](https://www.kegg.jp/kegg-bin/show_pathway?mmu05200) | Pathways in cancer | 6 of 522 | 0.76 | 0.0072 |

**SPINE**

**DOWN-EXPRESSED**

**CLUSTER 3**

**
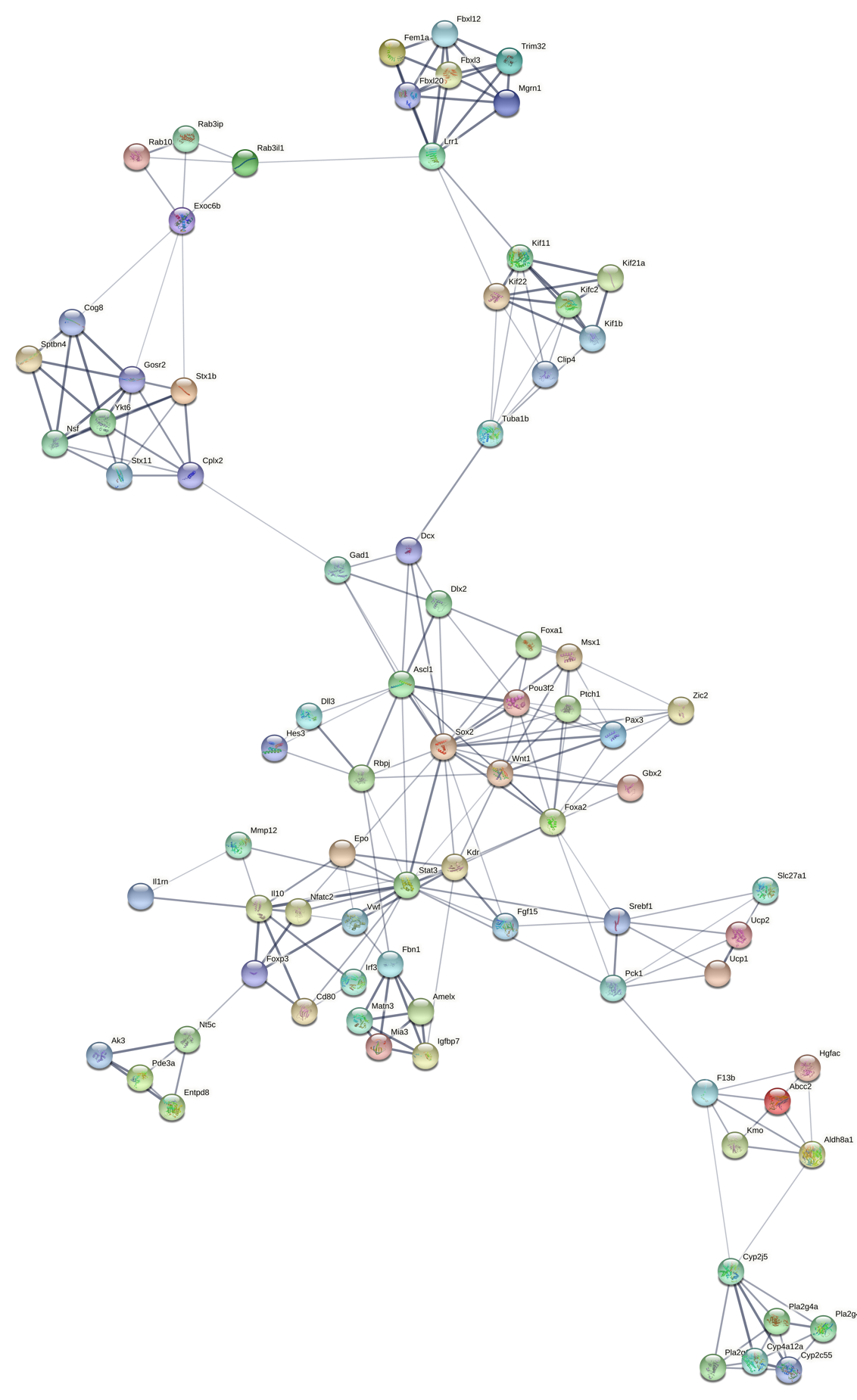
**

number of nodes: 80

number of edges: 226

average node degree: 5.65

avg. local clustering coefficient: 0.751

expected number of edges: 70

PPI enrichment p-value: < 1.0E^-16^

|  | **KEGG Pathways** |  |  |  |
| --- | --- | --- | --- | --- |
| ***pathway*** | ***description*** | ***count in network*** | ***strength*** | ***false discovery rate*** |
| [mmu00592](https://www.kegg.jp/kegg-bin/show_pathway?mmu00592) | alpha-Linolenic acid metabolism | 3 of 24 | 1.54 | 0.0032 |
| [mmu04130](https://www.kegg.jp/kegg-bin/show_pathway?mmu04130) | SNARE interactions in vesicular transport | 4 of 33 | 1.52 | 0.00048 |
| [mmu00591](https://www.kegg.jp/kegg-bin/show_pathway?mmu00591) | Linoleic acid metabolism | 5 of 48 | 1.46 | 0.00018 |
| [mmu04370](https://www.kegg.jp/kegg-bin/show_pathway?mmu04370) | VEGF signaling pathway | 4 of 58 | 1.28 | 0.0029 |
| [mmu00590](https://www.kegg.jp/kegg-bin/show_pathway?mmu00590) | Arachidonic acid metabolism | 6 of 88 | 1.27 | 0.00018 |
| [mmu00565](https://www.kegg.jp/kegg-bin/show_pathway?mmu00565) | Ether lipid metabolism | 3 of 45 | 1.26 | 0.0133 |
| [mmu04913](https://www.kegg.jp/kegg-bin/show_pathway?mmu04913) | Ovarian steroidogenesis | 3 of 57 | 1.16 | 0.0171 |
| [mmu05321](https://www.kegg.jp/kegg-bin/show_pathway?mmu05321) | Inflammatory bowel disease (IBD) | 3 of 58 | 1.15 | 0.0171 |
| [mmu04721](https://www.kegg.jp/kegg-bin/show_pathway?mmu04721) | Synaptic vesicle cycle | 3 of 62 | 1.13 | 0.0171 |
| [mmu03320](https://www.kegg.jp/kegg-bin/show_pathway?mmu03320) | PPAR signaling pathway | 4 of 85 | 1.11 | 0.0067 |
| [mmu04750](https://www.kegg.jp/kegg-bin/show_pathway?mmu04750) | Inflammatory mediator regulation of TRP channels | 5 of 119 | 1.06 | 0.0029 |
| [mmu04931](https://www.kegg.jp/kegg-bin/show_pathway?mmu04931) | Insulin resistance | 4 of 108 | 1.01 | 0.0133 |
| [mmu04658](https://www.kegg.jp/kegg-bin/show_pathway?mmu04658) | Th1 and Th2 cell differentiation | 3 of 86 | 0.98 | 0.0363 |
| [mmu04270](https://www.kegg.jp/kegg-bin/show_pathway?mmu04270) | Vascular smooth muscle contraction | 4 of 125 | 0.95 | 0.0171 |
| [mmu00564](https://www.kegg.jp/kegg-bin/show_pathway?mmu00564) | Glycerophospholipid metabolism | 3 of 95 | 0.94 | 0.0433 |
| [mmu04726](https://www.kegg.jp/kegg-bin/show_pathway?mmu04726) | Serotonergic synapse | 4 of 130 | 0.93 | 0.0171 |
| [mmu04659](https://www.kegg.jp/kegg-bin/show_pathway?mmu04659) | Th17 cell differentiation | 3 of 100 | 0.92 | 0.0472 |
| [mmu05169](https://www.kegg.jp/kegg-bin/show_pathway?mmu05169) | Epstein-Barr virus infection | 5 of 205 | 0.83 | 0.0151 |
| [mmu00230](https://www.kegg.jp/kegg-bin/show_pathway?mmu00230) | Purine metabolism | 4 of 175 | 0.8 | 0.0363 |
| [mmu04014](https://www.kegg.jp/kegg-bin/show_pathway?mmu04014) | Ras signaling pathway | 5 of 228 | 0.78 | 0.0171 |
| [mmu05205](https://www.kegg.jp/kegg-bin/show_pathway?mmu05205) | Proteoglycans in cancer | 4 of 199 | 0.74 | 0.0472 |

**HIND PAWS**

**UP-EXPRESSED**

**CLUSTER 1**

**
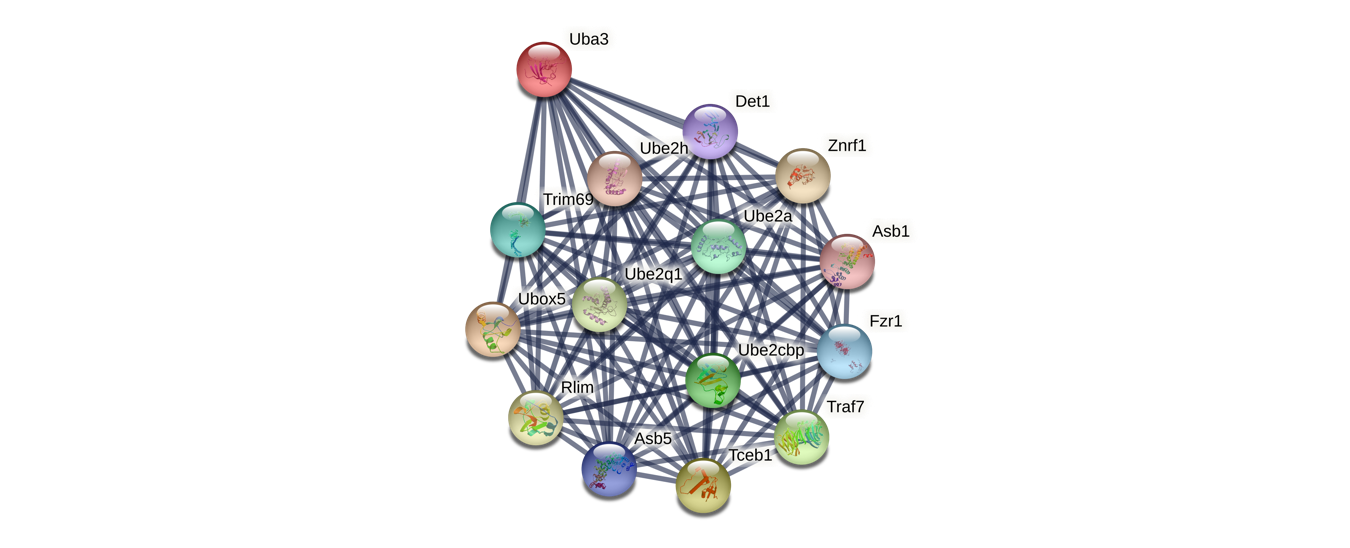
**

number of nodes: 15

number of edges: 105

average node degree: 14

avg. local clustering coefficient: 1

expected number of edges: 7

PPI enrichment p-value: < 1.0E^-16^

|  | **KEGG Pathways** |  |  |  |
| --- | --- | --- | --- | --- |
| ***pathway*** | ***description*** | ***count in network*** | ***strength*** | ***false discovery rate*** |
| [mmu04120](https://www.kegg.jp/kegg-bin/show_pathway?mmu04120) | Ubiquitin mediated proteolysis | 8 of 137 | 1.93 | 1.06e-13 |

**HIND PAWS**

**UP-EXPRESSED**

**CLUSTER 2**

**
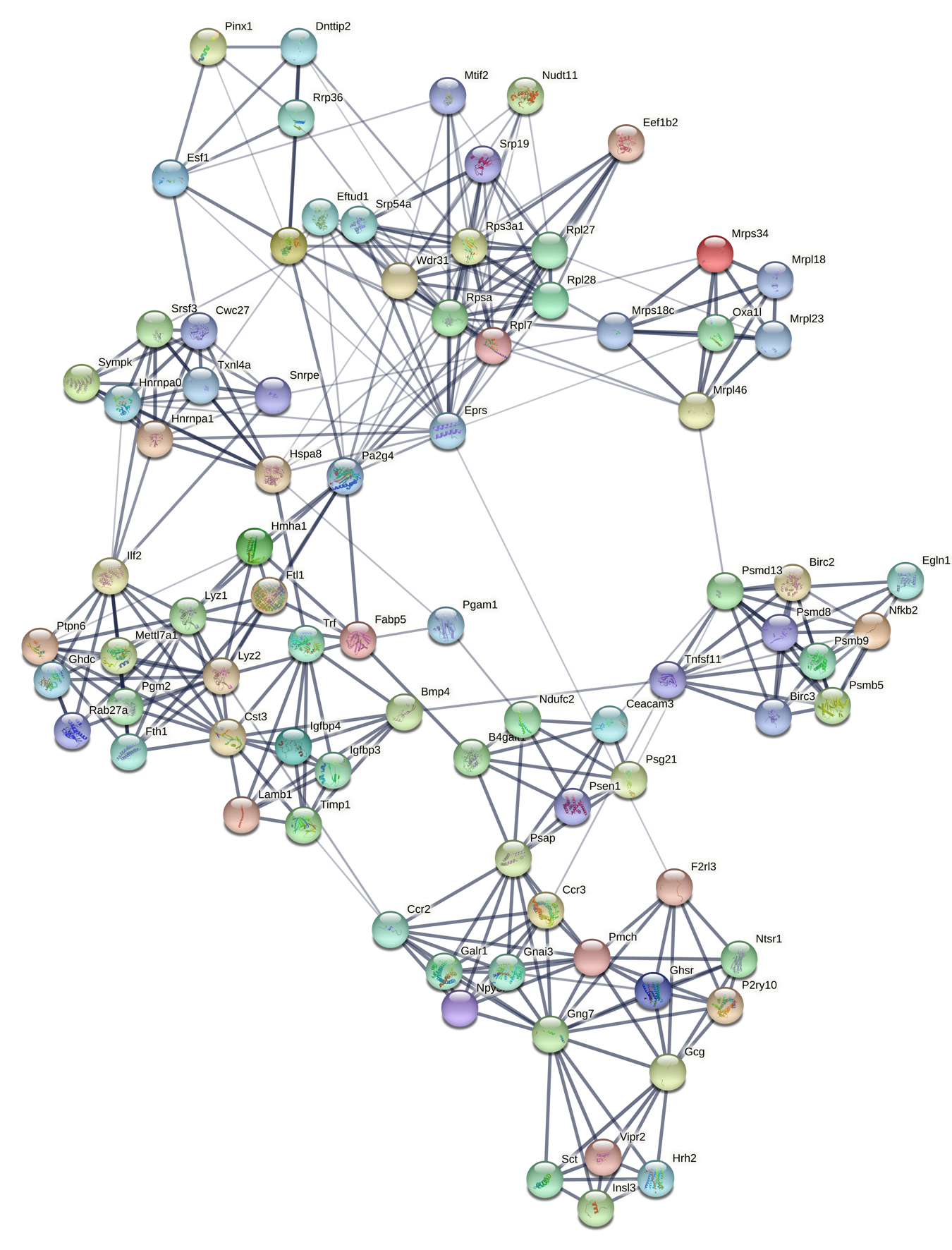
**

number of nodes: 84

number of edges: 351

average node degree: 8.36

avg. local clustering coefficient: 0.738

expected number of edges: 161

PPI enrichment p-value: < 1.0e-16

|  | **KEGG Pathways** |  |  |  |
| --- | --- | --- | --- | --- |
| ***pathway*** | ***description*** | ***count in network*** | ***strength*** | ***false discovery rate*** |
| [mmu03050](https://www.kegg.jp/kegg-bin/show_pathway?mmu03050) | Proteasome | 4 of 45 | 1.37 | 0.0016 |
| [mmu03060](https://www.kegg.jp/kegg-bin/show_pathway?mmu03060) | Protein export | 2 of 24 | 1.34 | 0.0476 |
| [mmu04216](https://www.kegg.jp/kegg-bin/show_pathway?mmu04216) | Ferroptosis | 3 of 40 | 1.29 | 0.0096 |
| [mmu04978](https://www.kegg.jp/kegg-bin/show_pathway?mmu04978) | Mineral absorption | 3 of 44 | 1.25 | 0.0110 |
| [mmu03010](https://www.kegg.jp/kegg-bin/show_pathway?mmu03010) | Ribosome | 7 of 128 | 1.16 | 0.00011 |
| [mmu05145](https://www.kegg.jp/kegg-bin/show_pathway?mmu05145) | Toxoplasmosis | 5 of 107 | 1.09 | 0.0022 |
| [mmu04064](https://www.kegg.jp/kegg-bin/show_pathway?mmu04064) | NF-kappa B signaling pathway | 4 of 93 | 1.05 | 0.0096 |
| [mmu04970](https://www.kegg.jp/kegg-bin/show_pathway?mmu04970) | Salivary secretion | 3 of 75 | 1.02 | 0.0428 |
| [mmu03040](https://www.kegg.jp/kegg-bin/show_pathway?mmu03040) | Spliceosome | 5 of 130 | 1.0 | 0.0043 |
| [mmu04080](https://www.kegg.jp/kegg-bin/show_pathway?mmu04080) | Neuroactive ligand-receptor interaction | 8 of 284 | 0.87 | 0.00100 |
| [mmu04217](https://www.kegg.jp/kegg-bin/show_pathway?mmu04217) | Necroptosis | 4 of 158 | 0.82 | 0.0428 |
| [mmu05200](https://www.kegg.jp/kegg-bin/show_pathway?mmu05200) | Pathways in cancer | 9 of 522 | 0.66 | 0.0043 |

**HIND PAWS**

**UP-EXPRESSED**

**CLUSTER 3**


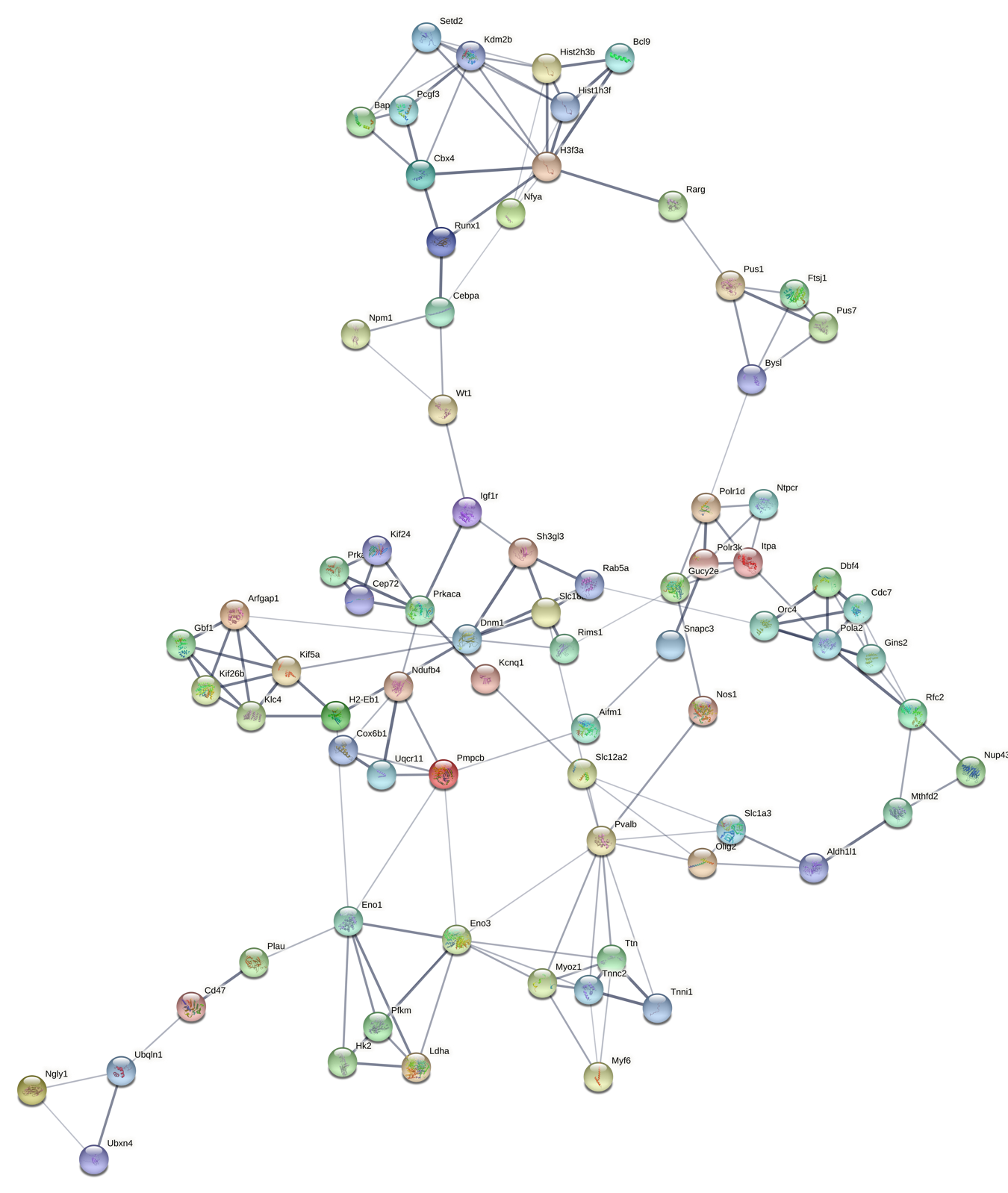


number of nodes: 76

number of edges: 159

average node degree: 4.18

avg. local clustering coefficient: 0.586

expected number of edges: 62

PPI enrichment p-value: < 1.0E^-16^

|  | **KEGG Pathways** |  |  |  |
| --- | --- | --- | --- | --- |
| ***pathway*** | ***description*** | ***count in network*** | ***strength*** | ***false discovery rate*** |
| [mmu00670](https://www.kegg.jp/kegg-bin/show_pathway?mmu00670) | One carbon pool by folate | 2 of 19 | 1.48 | 0.0315 |
| [mmu00010](https://www.kegg.jp/kegg-bin/show_pathway?mmu00010) | Glycolysis / Gluconeogenesis | 5 of 65 | 1.35 | 0.00069 |
| [mmu03020](https://www.kegg.jp/kegg-bin/show_pathway?mmu03020) | RNA polymerase | 2 of 28 | 1.32 | 0.0451 |
| [mmu04066](https://www.kegg.jp/kegg-bin/show_pathway?mmu04066) | HIF-1 signaling pathway | 5 of 102 | 1.15 | 0.0023 |
| [mmu04721](https://www.kegg.jp/kegg-bin/show_pathway?mmu04721) | Synaptic vesicle cycle | 3 of 62 | 1.15 | 0.0294 |
| [mmu05230](https://www.kegg.jp/kegg-bin/show_pathway?mmu05230) | Central carbon metabolism in cancer | 3 of 64 | 1.13 | 0.0294 |
| [mmu04970](https://www.kegg.jp/kegg-bin/show_pathway?mmu04970) | Salivary secretion | 3 of 75 | 1.06 | 0.0315 |
| [mmu01230](https://www.kegg.jp/kegg-bin/show_pathway?mmu01230) | Biosynthesis of amino acids | 3 of 75 | 1.06 | 0.0315 |
| [mmu03018](https://www.kegg.jp/kegg-bin/show_pathway?mmu03018) | RNA degradation | 3 of 80 | 1.04 | 0.0315 |
| [mmu05202](https://www.kegg.jp/kegg-bin/show_pathway?mmu05202) | Transcriptional misregulation in cancer | 6 of 167 | 1.02 | 0.0023 |
| [mmu00230](https://www.kegg.jp/kegg-bin/show_pathway?mmu00230) | Purine metabolism | 6 of 175 | 1.0 | 0.0023 |
| [mmu01200](https://www.kegg.jp/kegg-bin/show_pathway?mmu01200) | Carbon metabolism | 4 of 118 | 0.99 | 0.0191 |
| [mmu00240](https://www.kegg.jp/kegg-bin/show_pathway?mmu00240) | Pyrimidine metabolism | 3 of 98 | 0.95 | 0.0456 |
| [mmu05012](https://www.kegg.jp/kegg-bin/show_pathway?mmu05012) | Parkinson's disease | 4 of 138 | 0.92 | 0.0294 |
| [mmu04144](https://www.kegg.jp/kegg-bin/show_pathway?mmu04144) | Endocytosis | 7 of 258 | 0.9 | 0.0023 |
| [mmu04932](https://www.kegg.jp/kegg-bin/show_pathway?mmu04932) | Non-alcoholic fatty liver disease (NAFLD) | 4 of 146 | 0.9 | 0.0294 |
| [mmu05010](https://www.kegg.jp/kegg-bin/show_pathway?mmu05010) | Alzheimer's disease | 4 of 167 | 0.84 | 0.0315 |
| [mmu01100](https://www.kegg.jp/kegg-bin/show_pathway?mmu01100) | Metabolic pathways | 15 of 1296 | 0.53 | 0.0023 |

**HIND PAWS**

**DOWN-EXPRESSED**

**CLUSTER 1**

**
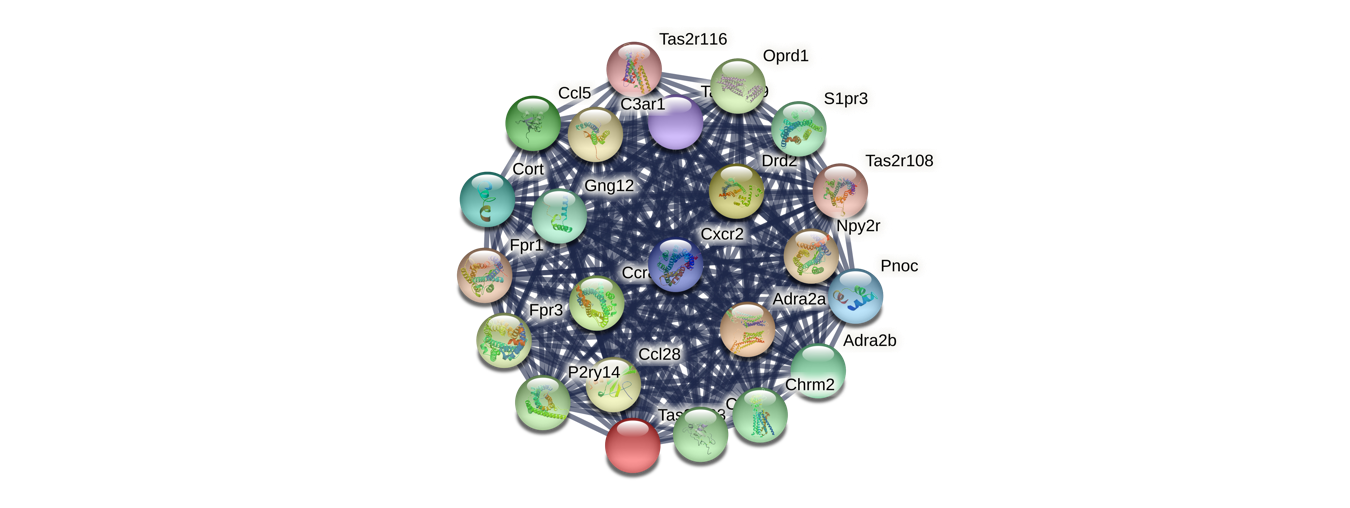
**

number of nodes: 23

number of edges: 253

average node degree: 22

avg. local clustering coefficient: 1

expected number of edges: 15

PPI enrichment p-value: < 1.0E^-16^

|  | **KEGG Pathways** |  |  |  |
| --- | --- | --- | --- | --- |
| ***pathway*** | ***description*** | ***count in network*** | ***strength*** | ***false discovery rate*** |
| [mmu05150](https://www.kegg.jp/kegg-bin/show_pathway?mmu05150) | Staphylococcus aureus infection | 3 of 50 | 1.76 | 0.00021 |
| [mmu04672](https://www.kegg.jp/kegg-bin/show_pathway?mmu04672) | Intestinal immune network for IgA production | 2 of 40 | 1.68 | 0.0059 |
| [mmu04742](https://www.kegg.jp/kegg-bin/show_pathway?mmu04742) | Taste transduction | 4 of 86 | 1.65 | 3.38e-05 |
| [mmu04080](https://www.kegg.jp/kegg-bin/show_pathway?mmu04080) | Neuroactive ligand-receptor interaction | 11 of 284 | 1.57 | 1.11e-13 |
| [mmu04062](https://www.kegg.jp/kegg-bin/show_pathway?mmu04062) | Chemokine signaling pathway | 6 of 179 | 1.51 | 6.74e-07 |
| [mmu05323](https://www.kegg.jp/kegg-bin/show_pathway?mmu05323) | Rheumatoid arthritis | 2 of 81 | 1.37 | 0.0197 |
| [mmu04060](https://www.kegg.jp/kegg-bin/show_pathway?mmu04060) | Cytokine-cytokine receptor interaction | 5 of 252 | 1.28 | 6.86e-05 |
| [mmu04022](https://www.kegg.jp/kegg-bin/show_pathway?mmu04022) | cGMP-PKG signaling pathway | 3 of 164 | 1.24 | 0.0053 |
| [mmu04725](https://www.kegg.jp/kegg-bin/show_pathway?mmu04725) | Cholinergic synapse | 2 of 112 | 1.23 | 0.0325 |
| [mmu04071](https://www.kegg.jp/kegg-bin/show_pathway?mmu04071) | Sphingolipid signaling pathway | 2 of 120 | 1.2 | 0.0334 |
| [mmu04728](https://www.kegg.jp/kegg-bin/show_pathway?mmu04728) | Dopaminergic synapse | 2 of 129 | 1.17 | 0.0348 |
| [mmu05034](https://www.kegg.jp/kegg-bin/show_pathway?mmu05034) | Alcoholism | 2 of 146 | 1.12 | 0.0404 |

**HIND PAWS**

**DOWN-EXPRESSED**

**CLUSTER 2**


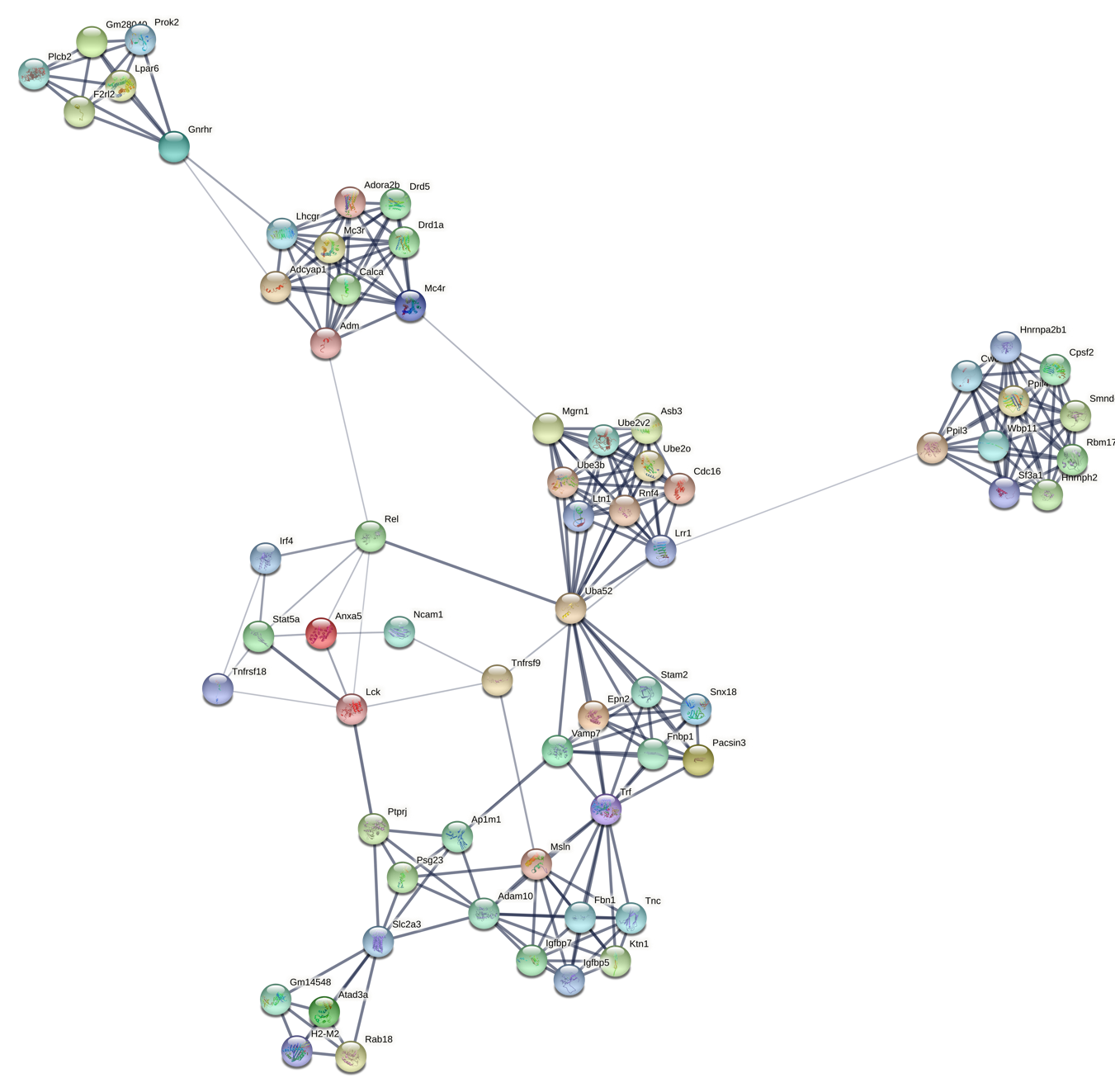


number of nodes: 65

number of edges: 243

average node degree: 7.48

avg. local clustering coefficient: 0.826

expected number of edges: 77

PPI enrichment p-value: < 1.0E^-16^

|  | **KEGG Pathways** |  |  |  |
| --- | --- | --- | --- | --- |
| ***pathway*** | ***description*** | ***count in network*** | ***strength*** | ***false discovery rate*** |
| [mmu03040](https://www.kegg.jp/kegg-bin/show_pathway?mmu03040) | Spliceosome | 5 of 130 | 1.12 | 0.0026 |
| [mmu04080](https://www.kegg.jp/kegg-bin/show_pathway?mmu04080) | Neuroactive ligand-receptor interaction | 9 of 284 | 1.03 | 2.03e-05 |
| [mmu04120](https://www.kegg.jp/kegg-bin/show_pathway?mmu04120) | Ubiquitin mediated proteolysis | 4 of 137 | 1.0 | 0.0216 |
| [mmu04020](https://www.kegg.jp/kegg-bin/show_pathway?mmu04020) | Calcium signaling pathway | 5 of 180 | 0.97 | 0.0078 |

**HIND PAWS**

**DOWN-EXPRESSED**

**CLUSTER 3**


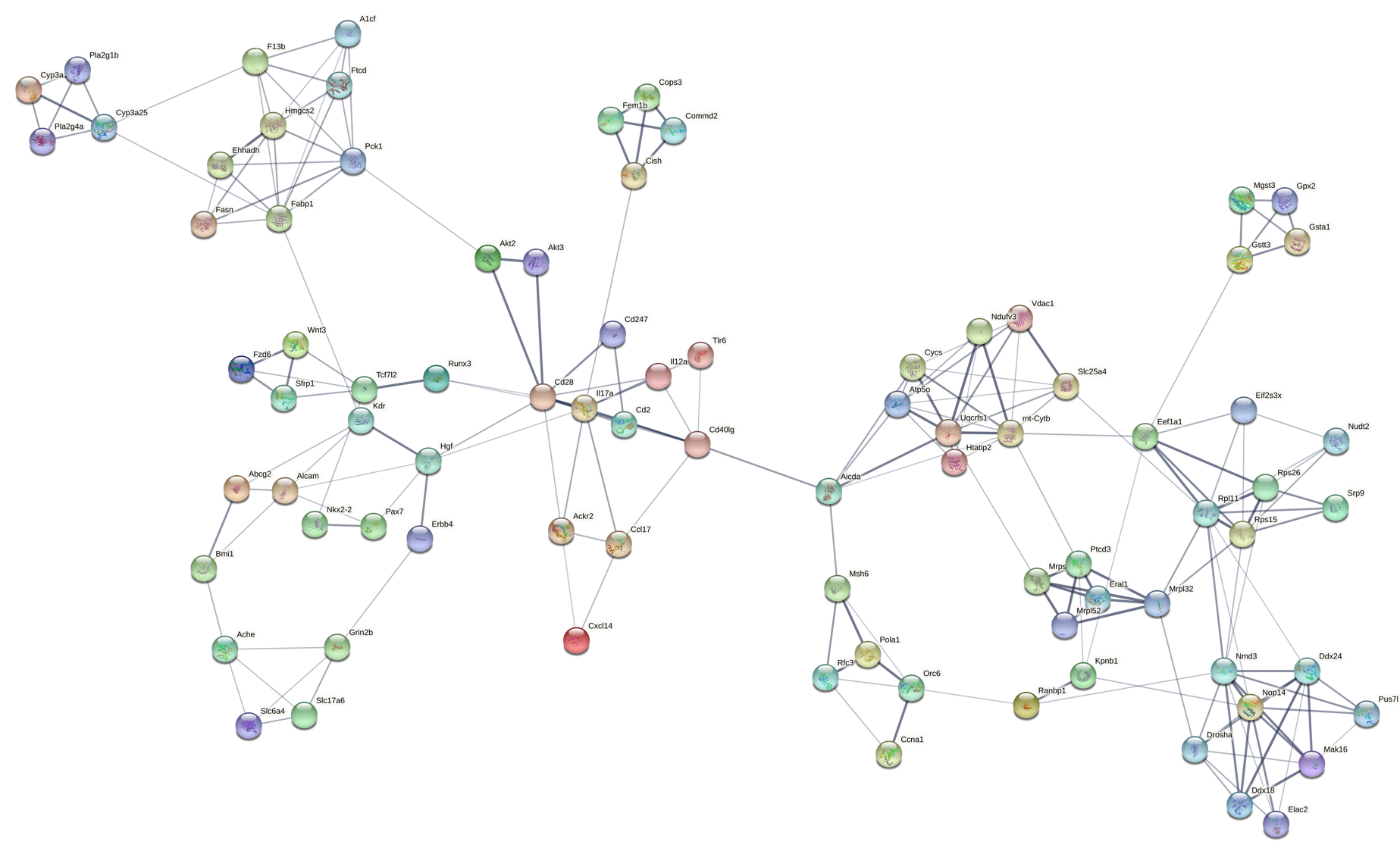


number of nodes: 85

number of edges: 202

average node degree: 4.75

avg. local clustering coefficient: 0.672

expected number of edges: 89

PPI enrichment p-value: < 1.0E^-16^

|  | **KEGG Pathways** |  |  |  |
| --- | --- | --- | --- | --- |
| ***pathway*** | ***description*** | ***count in network*** | ***strength*** | ***false discovery rate*** |
| [mmu01524](https://www.kegg.jp/kegg-bin/show_pathway?mmu01524) | Platinum drug resistance | 7 of 76 | 1.38 | 5.41e-06 |
| [mmu03430](https://www.kegg.jp/kegg-bin/show_pathway?mmu03430) | Mismatch repair | 2 of 22 | 1.37 | 0.0130 |
| [mmu00591](https://www.kegg.jp/kegg-bin/show_pathway?mmu00591) | Linoleic acid metabolism | 4 of 48 | 1.33 | 0.00074 |
| [mmu00592](https://www.kegg.jp/kegg-bin/show_pathway?mmu00592) | alpha-Linolenic acid metabolism | 2 of 24 | 1.33 | 0.0146 |
| [mmu04672](https://www.kegg.jp/kegg-bin/show_pathway?mmu04672) | Intestinal immune network for IgA production | 3 of 40 | 1.29 | 0.0039 |
| [mmu00650](https://www.kegg.jp/kegg-bin/show_pathway?mmu00650) | Butanoate metabolism | 2 of 27 | 1.28 | 0.0163 |
| [mmu04370](https://www.kegg.jp/kegg-bin/show_pathway?mmu04370) | VEGF signaling pathway | 4 of 58 | 1.25 | 0.00100 |
| [mmu05144](https://www.kegg.jp/kegg-bin/show_pathway?mmu05144) | Malaria | 3 of 45 | 1.24 | 0.0048 |
| [mmu00480](https://www.kegg.jp/kegg-bin/show_pathway?mmu00480) | Glutathione metabolism | 4 of 61 | 1.23 | 0.0011 |
| [mmu05221](https://www.kegg.jp/kegg-bin/show_pathway?mmu05221) | Acute myeloid leukemia | 4 of 69 | 1.18 | 0.0015 |
| [mmu05330](https://www.kegg.jp/kegg-bin/show_pathway?mmu05330) | Allograft rejection | 3 of 52 | 1.18 | 0.0068 |
| [mmu05340](https://www.kegg.jp/kegg-bin/show_pathway?mmu05340) | Primary immunodeficiency | 2 of 34 | 1.18 | 0.0217 |
| [mmu05210](https://www.kegg.jp/kegg-bin/show_pathway?mmu05210) | Colorectal cancer | 5 of 88 | 1.17 | 0.00067 |
| [mmu03030](https://www.kegg.jp/kegg-bin/show_pathway?mmu03030) | DNA replication | 2 of 35 | 1.17 | 0.0226 |
| [mmu05204](https://www.kegg.jp/kegg-bin/show_pathway?mmu05204) | Chemical carcinogenesis | 5 of 92 | 1.15 | 0.00067 |
| [mmu05225](https://www.kegg.jp/kegg-bin/show_pathway?mmu05225) | Hepatocellular carcinoma | 9 of 168 | 1.14 | 5.41e-06 |
| [mmu05134](https://www.kegg.jp/kegg-bin/show_pathway?mmu05134) | Legionellosis | 3 of 57 | 1.14 | 0.0083 |
| [mmu04975](https://www.kegg.jp/kegg-bin/show_pathway?mmu04975) | Fat digestion and absorption | 2 of 38 | 1.14 | 0.0250 |
| [mmu05213](https://www.kegg.jp/kegg-bin/show_pathway?mmu05213) | Endometrial cancer | 3 of 58 | 1.13 | 0.0085 |
| [mmu05012](https://www.kegg.jp/kegg-bin/show_pathway?mmu05012) | Parkinson's disease | 7 of 138 | 1.12 | 5.73e-05 |
| [mmu01521](https://www.kegg.jp/kegg-bin/show_pathway?mmu01521) | EGFR tyrosine kinase inhibitor resistance | 4 of 79 | 1.12 | 0.0023 |
| [mmu05142](https://www.kegg.jp/kegg-bin/show_pathway?mmu05142) | Chagas disease (American trypanosomiasis) | 5 of 101 | 1.11 | 0.00077 |
| [mmu04660](https://www.kegg.jp/kegg-bin/show_pathway?mmu04660) | T cell receptor signaling pathway | 5 of 100 | 1.11 | 0.00077 |
| [mmu05033](https://www.kegg.jp/kegg-bin/show_pathway?mmu05033) | Nicotine addiction | 2 of 40 | 1.11 | 0.0271 |
| [mmu04973](https://www.kegg.jp/kegg-bin/show_pathway?mmu04973) | Carbohydrate digestion and absorption | 2 of 40 | 1.11 | 0.0271 |
| [mmu03320](https://www.kegg.jp/kegg-bin/show_pathway?mmu03320) | PPAR signaling pathway | 4 of 85 | 1.09 | 0.0028 |
| [mmu05217](https://www.kegg.jp/kegg-bin/show_pathway?mmu05217) | Basal cell carcinoma | 3 of 63 | 1.09 | 0.0095 |
| [mmu05145](https://www.kegg.jp/kegg-bin/show_pathway?mmu05145) | Toxoplasmosis | 5 of 107 | 1.08 | 0.00081 |
| [mmu00980](https://www.kegg.jp/kegg-bin/show_pathway?mmu00980) | Metabolism of xenobiotics by cytochrome P450 | 3 of 65 | 1.08 | 0.0101 |
| [mmu04664](https://www.kegg.jp/kegg-bin/show_pathway?mmu04664) | Fc epsilon RI signaling pathway | 3 of 66 | 1.07 | 0.0103 |
| [mmu05211](https://www.kegg.jp/kegg-bin/show_pathway?mmu05211) | Renal cell carcinoma | 3 of 67 | 1.06 | 0.0104 |
| [mmu00982](https://www.kegg.jp/kegg-bin/show_pathway?mmu00982) | Drug metabolism - cytochrome P450 | 3 of 67 | 1.06 | 0.0104 |
| [mmu00565](https://www.kegg.jp/kegg-bin/show_pathway?mmu00565) | Ether lipid metabolism | 2 of 45 | 1.06 | 0.0321 |
| [mmu05016](https://www.kegg.jp/kegg-bin/show_pathway?mmu05016) | Huntington's disease | 8 of 187 | 1.05 | 5.73e-05 |
| [mmu05418](https://www.kegg.jp/kegg-bin/show_pathway?mmu05418) | Fluid shear stress and atherosclerosis | 6 of 140 | 1.05 | 0.00061 |
| [mmu05218](https://www.kegg.jp/kegg-bin/show_pathway?mmu05218) | Melanoma | 3 of 71 | 1.04 | 0.0112 |
| [mmu04920](https://www.kegg.jp/kegg-bin/show_pathway?mmu04920) | Adipocytokine signaling pathway | 3 of 71 | 1.04 | 0.0112 |
| [mmu04932](https://www.kegg.jp/kegg-bin/show_pathway?mmu04932) | Non-alcoholic fatty liver disease (NAFLD) | 6 of 146 | 1.03 | 0.00067 |
| [mmu04917](https://www.kegg.jp/kegg-bin/show_pathway?mmu04917) | Prolactin signaling pathway | 3 of 72 | 1.03 | 0.0112 |
| [mmu05205](https://www.kegg.jp/kegg-bin/show_pathway?mmu05205) | Proteoglycans in cancer | 8 of 199 | 1.02 | 5.73e-05 |
| [mmu05226](https://www.kegg.jp/kegg-bin/show_pathway?mmu05226) | Gastric cancer | 6 of 148 | 1.02 | 0.00067 |
| [mmu04152](https://www.kegg.jp/kegg-bin/show_pathway?mmu04152) | AMPK signaling pathway | 5 of 124 | 1.02 | 0.0012 |
| [mmu04620](https://www.kegg.jp/kegg-bin/show_pathway?mmu04620) | Toll-like receptor signaling pathway | 4 of 98 | 1.02 | 0.0039 |
| [mmu05416](https://www.kegg.jp/kegg-bin/show_pathway?mmu05416) | Viral myocarditis | 3 of 76 | 1.01 | 0.0125 |
| [mmu01212](https://www.kegg.jp/kegg-bin/show_pathway?mmu01212) | Fatty acid metabolism | 2 of 51 | 1.01 | 0.0382 |
| [mmu05014](https://www.kegg.jp/kegg-bin/show_pathway?mmu05014) | Amyotrophic lateral sclerosis (ALS) | 2 of 52 | 1.0 | 0.0392 |
| [mmu04550](https://www.kegg.jp/kegg-bin/show_pathway?mmu04550) | Signaling pathways regulating pluripotency of stem cells | 5 of 137 | 0.98 | 0.0018 |
| [mmu05010](https://www.kegg.jp/kegg-bin/show_pathway?mmu05010) | Alzheimer's disease | 6 of 167 | 0.97 | 0.00077 |
| [mmu04666](https://www.kegg.jp/kegg-bin/show_pathway?mmu04666) | Fc gamma R-mediated phagocytosis | 3 of 83 | 0.97 | 0.0146 |
| [mmu04012](https://www.kegg.jp/kegg-bin/show_pathway?mmu04012) | ErbB signaling pathway | 3 of 83 | 0.97 | 0.0146 |
| [mmu04923](https://www.kegg.jp/kegg-bin/show_pathway?mmu04923) | Regulation of lipolysis in adipocytes | 2 of 55 | 0.97 | 0.0425 |
| [mmu00280](https://www.kegg.jp/kegg-bin/show_pathway?mmu00280) | Valine, leucine and isoleucine degradation | 2 of 55 | 0.97 | 0.0425 |
| [mmu04658](https://www.kegg.jp/kegg-bin/show_pathway?mmu04658) | Th1 and Th2 cell differentiation | 3 of 86 | 0.96 | 0.0153 |
| [mmu00983](https://www.kegg.jp/kegg-bin/show_pathway?mmu00983) | Drug metabolism - other enzymes | 3 of 86 | 0.96 | 0.0153 |
| [mmu05224](https://www.kegg.jp/kegg-bin/show_pathway?mmu05224) | Breast cancer | 5 of 146 | 0.95 | 0.0023 |
| [mmu00590](https://www.kegg.jp/kegg-bin/show_pathway?mmu00590) | Arachidonic acid metabolism | 3 of 88 | 0.95 | 0.0158 |
| [mmu05321](https://www.kegg.jp/kegg-bin/show_pathway?mmu05321) | Inflammatory bowel disease (IBD) | 2 of 58 | 0.95 | 0.0457 |
| [mmu04914](https://www.kegg.jp/kegg-bin/show_pathway?mmu04914) | Progesterone-mediated oocyte maturation | 3 of 90 | 0.94 | 0.0163 |
| [mmu04940](https://www.kegg.jp/kegg-bin/show_pathway?mmu04940) | Type I diabetes mellitus | 2 of 59 | 0.94 | 0.0463 |
| [mmu05322](https://www.kegg.jp/kegg-bin/show_pathway?mmu05322) | Systemic lupus erythematosus | 3 of 92 | 0.93 | 0.0168 |
| [mmu05222](https://www.kegg.jp/kegg-bin/show_pathway?mmu05222) | Small cell lung cancer | 3 of 92 | 0.93 | 0.0168 |
| [mmu04213](https://www.kegg.jp/kegg-bin/show_pathway?mmu04213) | Longevity regulating pathway - multiple species | 2 of 62 | 0.92 | 0.0492 |
| [mmu00190](https://www.kegg.jp/kegg-bin/show_pathway?mmu00190) | Oxidative phosphorylation | 4 of 129 | 0.91 | 0.0086 |
| [mmu00564](https://www.kegg.jp/kegg-bin/show_pathway?mmu00564) | Glycerophospholipid metabolism | 3 of 95 | 0.91 | 0.0178 |
| [mmu04014](https://www.kegg.jp/kegg-bin/show_pathway?mmu04014) | Ras signaling pathway | 7 of 228 | 0.9 | 0.00067 |
| [mmu05164](https://www.kegg.jp/kegg-bin/show_pathway?mmu05164) | Influenza A | 5 of 165 | 0.9 | 0.0034 |
| [mmu03013](https://www.kegg.jp/kegg-bin/show_pathway?mmu03013) | RNA transport | 5 of 164 | 0.9 | 0.0034 |
| [mmu05162](https://www.kegg.jp/kegg-bin/show_pathway?mmu05162) | Measles | 4 of 132 | 0.9 | 0.0091 |
| [mmu05215](https://www.kegg.jp/kegg-bin/show_pathway?mmu05215) | Prostate cancer | 3 of 97 | 0.9 | 0.0185 |
| [mmu05231](https://www.kegg.jp/kegg-bin/show_pathway?mmu05231) | Choline metabolism in cancer | 3 of 98 | 0.9 | 0.0188 |
| [mmu04922](https://www.kegg.jp/kegg-bin/show_pathway?mmu04922) | Glucagon signaling pathway | 3 of 99 | 0.9 | 0.0188 |
| [mmu04916](https://www.kegg.jp/kegg-bin/show_pathway?mmu04916) | Melanogenesis | 3 of 98 | 0.9 | 0.0188 |
| [mmu04910](https://www.kegg.jp/kegg-bin/show_pathway?mmu04910) | Insulin signaling pathway | 4 of 135 | 0.89 | 0.0095 |
| [mmu05152](https://www.kegg.jp/kegg-bin/show_pathway?mmu05152) | Tuberculosis | 5 of 172 | 0.88 | 0.0039 |
| [mmu04218](https://www.kegg.jp/kegg-bin/show_pathway?mmu04218) | Cellular senescence | 5 of 171 | 0.88 | 0.0039 |
| [mmu04060](https://www.kegg.jp/kegg-bin/show_pathway?mmu04060) | Cytokine-cytokine receptor interaction | 7 of 252 | 0.86 | 0.00077 |
| [mmu05161](https://www.kegg.jp/kegg-bin/show_pathway?mmu05161) | Hepatitis B | 4 of 143 | 0.86 | 0.0104 |
| [mmu04931](https://www.kegg.jp/kegg-bin/show_pathway?mmu04931) | Insulin resistance | 3 of 108 | 0.86 | 0.0226 |
| [mmu04310](https://www.kegg.jp/kegg-bin/show_pathway?mmu04310) | Wnt signaling pathway | 4 of 146 | 0.85 | 0.0105 |
| [mmu04725](https://www.kegg.jp/kegg-bin/show_pathway?mmu04725) | Cholinergic synapse | 3 of 112 | 0.84 | 0.0245 |
| [mmu04724](https://www.kegg.jp/kegg-bin/show_pathway?mmu04724) | Glutamatergic synapse | 3 of 113 | 0.84 | 0.0248 |
| [mmu05166](https://www.kegg.jp/kegg-bin/show_pathway?mmu05166) | HTLV-I infection | 7 of 269 | 0.83 | 0.00100 |
| [mmu04150](https://www.kegg.jp/kegg-bin/show_pathway?mmu04150) | mTOR signaling pathway | 4 of 152 | 0.83 | 0.0112 |
| [mmu04514](https://www.kegg.jp/kegg-bin/show_pathway?mmu04514) | Cell adhesion molecules (CAMs) | 4 of 158 | 0.82 | 0.0125 |
| [mmu04630](https://www.kegg.jp/kegg-bin/show_pathway?mmu04630) | Jak-STAT signaling pathway | 4 of 161 | 0.81 | 0.0130 |
| [mmu04015](https://www.kegg.jp/kegg-bin/show_pathway?mmu04015) | Rap1 signaling pathway | 5 of 207 | 0.8 | 0.0072 |
| [mmu04022](https://www.kegg.jp/kegg-bin/show_pathway?mmu04022) | cGMP-PKG signaling pathway | 4 of 164 | 0.8 | 0.0134 |
| [mmu04611](https://www.kegg.jp/kegg-bin/show_pathway?mmu04611) | Platelet activation | 3 of 122 | 0.8 | 0.0286 |
| [mmu05200](https://www.kegg.jp/kegg-bin/show_pathway?mmu05200) | Pathways in cancer | 12 of 522 | 0.78 | 5.73e-05 |
| [mmu04728](https://www.kegg.jp/kegg-bin/show_pathway?mmu04728) | Dopaminergic synapse | 3 of 129 | 0.78 | 0.0321 |
| [mmu03010](https://www.kegg.jp/kegg-bin/show_pathway?mmu03010) | Ribosome | 3 of 128 | 0.78 | 0.0321 |
| [mmu04068](https://www.kegg.jp/kegg-bin/show_pathway?mmu04068) | FoxO signaling pathway | 3 of 132 | 0.77 | 0.0336 |
| [mmu04062](https://www.kegg.jp/kegg-bin/show_pathway?mmu04062) | Chemokine signaling pathway | 4 of 179 | 0.76 | 0.0162 |
| [mmu04210](https://www.kegg.jp/kegg-bin/show_pathway?mmu04210) | Apoptosis | 3 of 135 | 0.76 | 0.0353 |
| [mmu04010](https://www.kegg.jp/kegg-bin/show_pathway?mmu04010) | MAPK signaling pathway | 6 of 292 | 0.73 | 0.0057 |
| [mmu04510](https://www.kegg.jp/kegg-bin/show_pathway?mmu04510) | Focal adhesion | 4 of 195 | 0.73 | 0.0190 |
| [mmu04072](https://www.kegg.jp/kegg-bin/show_pathway?mmu04072) | Phospholipase D signaling pathway | 3 of 145 | 0.73 | 0.0411 |
| [mmu04390](https://www.kegg.jp/kegg-bin/show_pathway?mmu04390) | Hippo signaling pathway | 3 of 153 | 0.71 | 0.0457 |
| [mmu04934](https://www.kegg.jp/kegg-bin/show_pathway?mmu04934) | Cushing's syndrome | 3 of 156 | 0.7 | 0.0466 |
| [mmu04217](https://www.kegg.jp/kegg-bin/show_pathway?mmu04217) | Necroptosis | 3 of 158 | 0.69 | 0.0476 |
| [mmu05165](https://www.kegg.jp/kegg-bin/show_pathway?mmu05165) | Human papillomavirus infection | 6 of 335 | 0.67 | 0.0095 |
| [mmu04714](https://www.kegg.jp/kegg-bin/show_pathway?mmu04714) | Thermogenesis | 4 of 223 | 0.67 | 0.0271 |
| [mmu04151](https://www.kegg.jp/kegg-bin/show_pathway?mmu04151) | PI3K-Akt signaling pathway | 6 of 349 | 0.65 | 0.0104 |
| [mmu01100](https://www.kegg.jp/kegg-bin/show_pathway?mmu01100) | Metabolic pathways | 15 of 1296 | 0.48 | 0.0012 |
