# Supplementary figures and images for: Levofloxacin induces differential effects in the transcriptome between the gut, peripheral and axial joints in the Spondyloarthritis DBA/1 mice: Improvement of intestinal dysbiosis and the overall inflammatory process
Source: PLoS One. 2023 Feb 2;18(2):e0281265. doi: 10.1371/journal.pone.0281265 (PMC9894406; doi:10.1371/journal.pone.0281265)

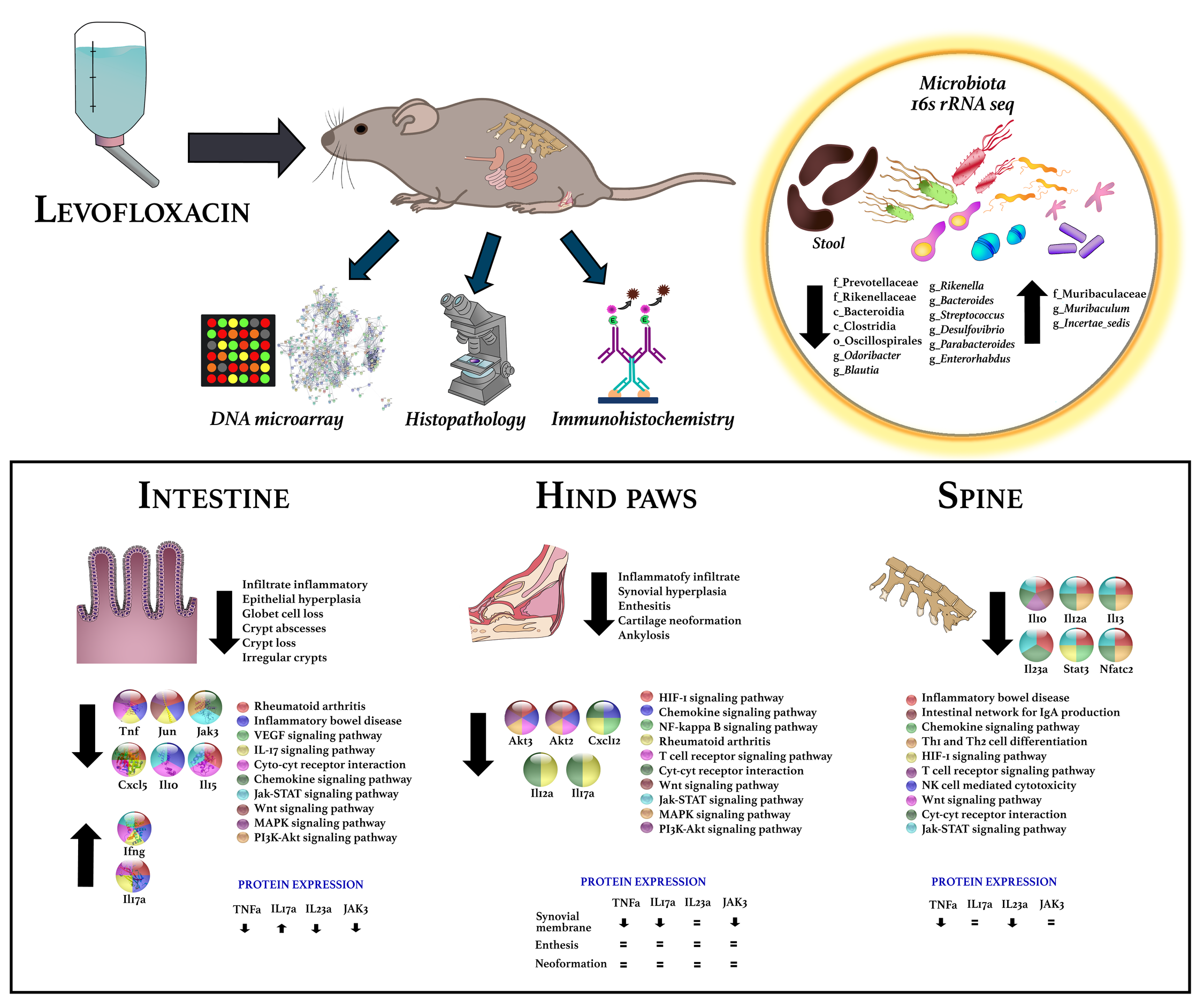

Supplement: S1 Graphical abstract — (TIF) [file pone.0281265.s005.tif]
